# Predictive Amphiphile Design Enables Control of Self‐Assembled Nanoparticle‐Cell Membrane Interactions

**DOI:** 10.1002/smll.75376

**Published:** 2026-08-22

**Authors:** Mahsa Nami, Tiffany Guitton‐Spassky, Farhad Sharif, Natalie Hanheiser, Riensqia Saudale, Jannik Gabriel, Aarti, Alireza Mirzaalipour, MohammadJavad Hafezi, Boris Schade, Abhishek Kumar Singh, Rainer Haag, Roland R. Netz, Hesam Makki

**Affiliations:** ^1^ Department of Polymer and Color Engineering Amirkabir University of Technology Tehran Iran; ^2^ Institut Für Chemie und Biochemie Organische Chemie Freie Universität Berlin Berlin Germany; ^3^ Department of Chemical Engineering Soft Matter Rheology and Technology KU Leuven Leuven Belgium; ^4^ Institut Für Chemie und Biochemie Forschungszentrum Für Elektronenmikroskopie Freie Universität Berlin Berlin Germany; ^5^ School of Biosciences Chanakya University Bengaluru India; ^6^ Department of Physics Freie Universitätät Berlin Berlin Germany; ^7^ Department of Chemistry and Materials Innovation Factory University of Liverpool Liverpool UK; ^8^ Department of Chemical Engineering University of Bath Bath UK

**Keywords:** amphiphilic molecule, drug delivery, molecular modelling, nanocarriers, self‐assembly

## Abstract

Despite decades of development, the clinical translation of amphiphilic self‐assembled nanoparticles remains limited by the lack of a predictive molecular design framework, owing to the complex, dynamic behavior of nanoparticles and cell membranes. Here, we introduce a building‐block design strategy focused on amphiphile monomers rather than assembled nanoparticles, a critical distinction because nanoparticle morphology is not a fixed design parameter. This molecular framework links amphiphile chemical structure to programmable cellular interactions. Through predictive variation of headgroup and tail chemistries, we demonstrate that amphiphile design dictates nanoparticle fate at cell membranes. Fully fluorinated nanoparticles remain intact and adsorb onto the membrane surface, hydrocarbon nanoparticles rapidly dissociate and fuse, whereas semi‐fluorinated nanoparticles exhibit tunable kinetic delays, enabling programmable responses such as stability at rigid interfaces but dissociation at fluid membranes. Crucially, we identify the free energy of transfer (Δ*G*_transfer_)–the energetic difference between extracting an amphiphile from the nanoparticle and from the cell membrane–as a unified thermodynamic descriptor that accurately predicts behavior across simulations and experiments without requiring full‐system modeling. Collectively, this predictive, chemistry‐driven framework shifts the design paradigm from nanoparticle morphology to amphiphile chemistry, enabling the rational engineering of self‐assembled nanocarriers for targeted drug delivery and responsive drug release applications.

## Graphical abstract

The interactions of self‐assembled nanocarriers with cell membranes are programmable through amphiphile design. We identify how head and tail chemistry govern distinct interaction mechanisms and introduce ΔG_transfer_, a unified thermodynamic descriptor that predicts nanoparticle behaviour from simple molecular simulations.

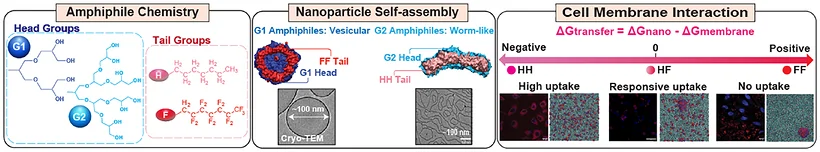

## 1 Introduction

While nanoparticle (NP) interactions with cell membranes have been widely examined through parameters such as size [1, 2], shape [3, 4], surface charge [5, 6], and surface functionalization [7, 8], these studies have mainly considered static, rigid NPs with predefined geometries [1, 2, 3, 4, 5, 6, 7, 8, 9, 10, 11]. In contrast, the biological fate of dynamic self‐assembled NPs, whose physical and geometrical features can adapt or reorganize upon interaction with the biological environment, is inherently governed by the molecular chemistry of their amphiphilic building blocks—Specifically, the structure of the hydrophobic tails and hydrophilic headgroups – [12, 13, 14] rather than by geometric and physical descriptors alone. Yet, despite extensive studies on static, rigid NPs with fixed geometries [1, 2, 3, 4, 5, 6, 7, 8, 9, 10, 11], systematic investigations establishing molecularly driven design frameworks that link amphiphile chemistry to programmable nano‐bio interaction behaviors remain absent, representing a critical gap in predictive nanomaterial design.

In amphiphilic systems, the molecular architecture of both the hydrophilic head group and the hydrophobic tail critically determine how these amphiphiles self‐assemble into NPs and how the resulting structures interact with cellular membranes [15, 16]. While polyethylene glycol (PEG) remains the most widely used headgroup for imparting stealth properties [17, 18, 19], polyglycerol has emerged as a superior alternative, offering enhanced protein resistance and biocompatibility and, due to the multiple hydroxyl groups present in PG, versatile post‐functionalization [20, 21, 22]. On the hydrophobic side, tail composition influences NP stability [12, 23], and interactions in biological environments [24]. Fluorinated amphiphiles, in particular, have received increasing attention due to the dual hydrophobic and lipophobic character of perfluorocarbons, which arises from their conformational rigidity and low polarizability [23, 25]. This unique omniphobic behavior promotes strong phase segregation, yielding highly stable, well‐defined self‐assembled structures that resist premature dissociation and display distinctive modes of membrane interaction [14, 26, 27]. Such properties make fluorinated tails an attractive handle for tuning NP behavior in complex biological media [28, 29].

The dynamism of the biological environment adds another layer of complexity to self‐assembled NP–cell membrane interactions. Cellular membranes are heterogeneous and asymmetric, with lipid distributions that vary across cell types, which respond differently to physiological or pathological conditions [30, 31, 32]. This structural and compositional variability, particularly the complex role of cholesterol in modulating membrane rigidity, organization, and fluidity [33, 34, 35, 36], can significantly influence the energetics and entropy of amphiphile insertion. For instance, while cholesterol may enhance surface roughness and promote adsorption on or into cell membranes [37], it also suppresses local lipid fluctuations, thereby increasing the energetic cost of spontaneous incorporation of amphiphiles [38].

Together, the coupled dynamic effects of NP and cell membrane demand molecular level methodologies capable of resolving amphiphile rearrangement, membrane restructuring, and insertion energetics [39, 40, 41]. Currently, amphiphile development for biomedical applications operates on an empirical, trial‐and‐error basis due to the lack of quantitative principles predicting how molecular architecture translates to the biological fate of self‐assembled NPs. This leaves synthetic efforts unguided and unable to rationally program desired nano‐bio interactions, necessitating the identification of key thermodynamic and kinetic descriptors to establish structure‐function relationships for engineering NPs with programmable biological behavior.

In this study, we design a library of six amphiphiles featuring dendritic oligoglycerol‐based headgroups (G1 and G2) and diverse tail chemistries i.e., fully fluorinated (FF), semi‐fluorinated (HF), and hydrocarbon (HH), alongside five representative cell membranes, varying lipid composition and organization and cholesterol content. Through a combination of molecular simulations, experimental investigations, and thermodynamic modeling, we (i) demonstrate that NP and cell interaction behavior is strongly determined by the underlying amphiphile design, and (ii) establish clear structure–function relationships, elucidating the distinct contributions of headgroup and tail chemistry in tuning NP‐cell interaction behavior, which can be modulated according to membrane composition and architecture. Importantly, we introduce a novel and computationally accessible metric derived from independent NP and membrane MD simulations that enables the prediction of NP‐cell interaction regimes without the need for full NP‐membrane system modeling. Furthermore, we identify cholesterol‐dependent kinetic thresholds for amphiphile insertion and headgroup‐mediated control over membrane penetration, providing design principles for programmable responses, i.e., assemblies that remain intact at rigid interfaces yet dissociate at fluid membranes, or engage without disrupting the membrane bilayer

## 2 Results and Discussion

### 2.1 Rational Design and Model Systems

To establish predictive structure‐function relationships governing NP‐cell membrane interactions, we designed a library of six amphiphilic molecules with systematically varied molecular architecture. Each amphiphile consists of a dendritic oligoglycerol headgroup of first (G1) or second generation (G2), combined with one of three binary hydrophobic tails: hydrocarbon‐hydrocarbon (HH), hydrocarbon‐fluorocarbon (HF), or fluorocarbon‐fluorocarbon (FF) segments (Figure 1A). This combinatorial design enables the decoupling of the steric and hydrophilic contributions of the headgroup and hydrophobic effects from tail chemistry on supramolecular assembly and biological behavior.

**FIGURE 1:**
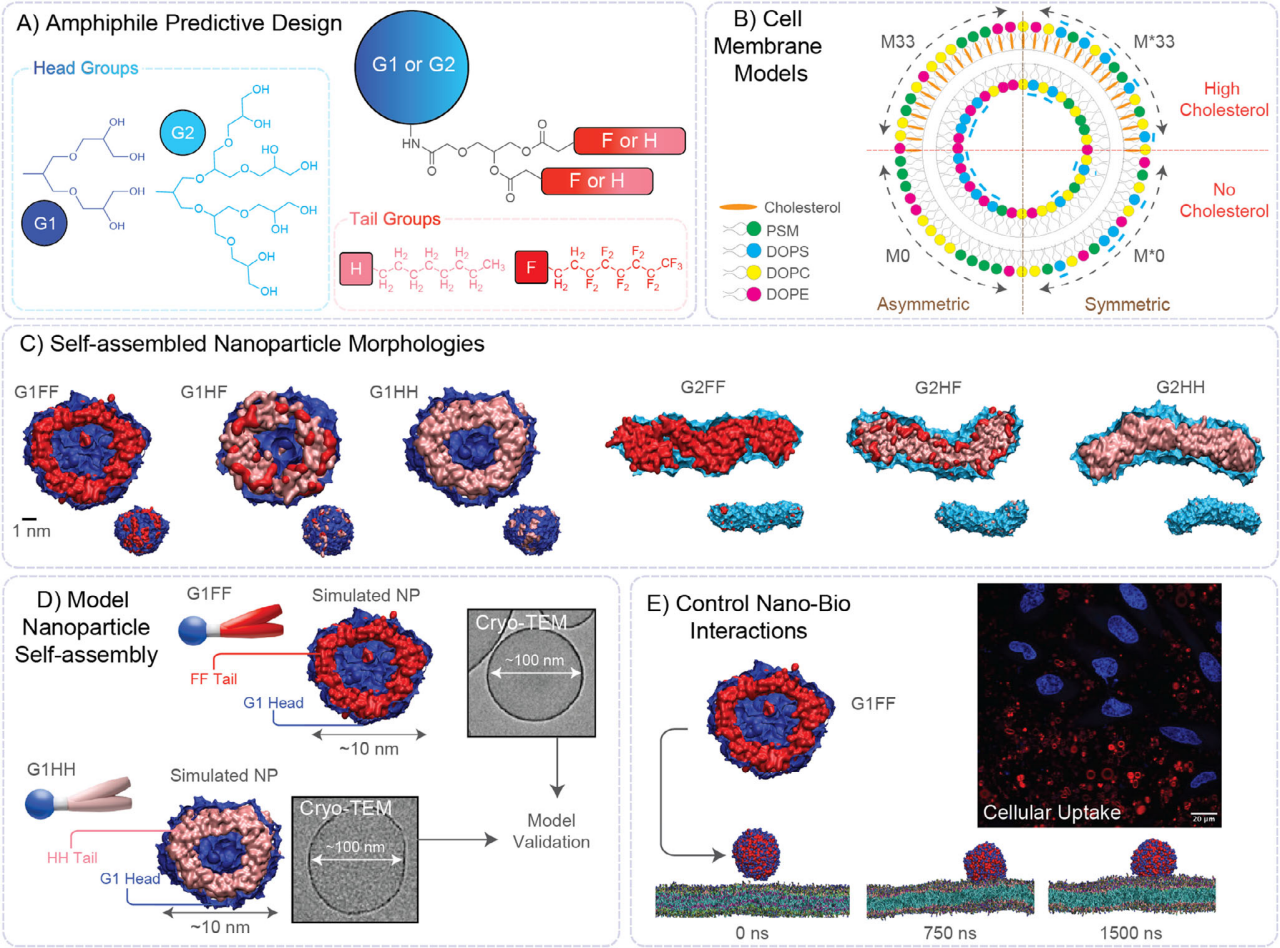
Integrated molecular design and combined experimental–computational framework for NP–membrane interactions. (A) Molecular structures of amphiphiles varying in headgroup generation (G1, G2) and tail composition (HH, HF, FF). (B) Membrane model structures mimicking mammalian erythrocyte composition: asymmetric M‐type (M0, M33) and symmetric M^*^‐type (M^*^0, M^*^33) membranes with 0 or 33 mol.% cholesterol. (C) Simulation snapshots of self‐assembled NP morphologies: Cross‐sectional and full CG representations of first‐generation NPs (G1HH, G1HF, G1FF) and second‐generation NPs (G2HH, G2HF, G2FF). (D) Snapshot of equilibrated self‐assembled NP morphologies from CG‐MD simulations, showing G1FF and G1HH vesicles (water is excluded), with experimental cryo‐TEM images. (E) Representative simulation snapshot of G1FF and experimental confocal microscopy image demonstrating NP–membrane interactions with negligible cellular uptake.

Model membranes were constructed to mimic mammalian erythrocyte compositions (Figure 1B; Table S5), following previous studies [42]. M‐type membranes (M0, M33) replicate normal mammalian plasma membrane asymmetry, with sphingomyelin and DOPC enriched in the outer leaflet characteristic of erythrocytes. M^*^‐type membranes (M^*^0, M^*^33) model cancer‐associated lipid redistribution, where elevated PS/PE externalization reduces membrane asymmetry—a hallmark of tumor cell surfaces that influences therapeutic targeting [43, 44, 45]. Cholesterol content was fixed at 0 or physiological representative 33 mol.% for mammalian cells [46, 47], to modulate membrane rigidity and lipid packing. A pure DOPC membrane was also included as a maximally fluid reference system, consistent with standard modelling practices [36, 38, 48]. This selection of membranes allows us to systematically probe how lipid asymmetry, membrane composition, and cholesterol content influence NP–membrane interactions.

Coarse‐grained molecular dynamics simulations (details in Method) were used to model (i) the self‐assembly of the amphiphiles into NPs in water and (ii) the subsequent NP interactions with the model membranes. In CG‐MD simulations, small groups of atoms are represented by single “beads”, which reduces computational cost and allows simulation of larger systems and longer timescales. We employed the Martini 3 force field [49, 50], which is commonly used for biomolecular and soft‐matter systems [51, 52, 53]. For self‐assembly studies, amphiphile molecules were randomly dispersed in water and simulated under internal normal human body conditions (310 K and 1 bar [54]; more details in Method) until stable NPs formed.

Our results reveal that headgroup generation, rather than tail chemistry, primarily determines the resulting aggregate morphology, consistent with previous works [13, 55]. As illustrated in Figure 1C, G1 amphiphiles spontaneously formed bilayered vesicles with internal aqueous compartments, whereas the bulkier G2 headgroups induced higher interfacial curvature, favoring elongated worm‐like micelles. The divergence in self‐assembled morphology—vesicular versus cylindrical—arises from a variation of the critical packing parameter (*P* = *v*/*a*_0_ *l*_c_), where the increased headgroup area (*a*_0_) of G2 relative to G1 shifts the *P* below 0.5, thermodynamically favoring high‐curvature structures [56].

Amphiphiles G1HH and G1FF were synthesized according to procedures described in Sections S11 and S12 and characterized (NMR included in Section S14). A semi‐fluorinated amphiphile, G1HF, was additionally synthesized following the route of Rashmi et al. [13] (Section S11), to directly test the intermediate interaction regime predicted for HF‐tailed systems (Section 2.3). The amphiphiles were then formulated in aqueous solution and characterized using dynamic light scattering (DLS, Section S12). For more precise determination of the self‐assembly, cryogenic transmission electron microscopy (cryo‐TEM) analysis of representative amphiphiles (G1HH, G1FF, and G1HF) confirmed the predicted vesicular morphologies for G1‐based amphiphiles with HH, FF, and HF tails (G1HH and G1FF shown in Figure 1D; G1HF in Figure S21; experimental details in the Method section and additional cryo‐TEM images in Section S12). The focus of this work is NP–membrane interaction behavior, for which G1HH, G1FF and G1HF serve as experimentally validated mechanistic benchmarks representing the two extremes of tail chemistry. The morphological predictions for the G2 systems are supported by prior experimental validation [13], where cryo‐TEM imaging of structurally related G2‐based amphiphiles confirmed worm‐like and branched network morphologies for G2HF and G2FF systems, respectively, in direct agreement with CG‐MD predictions.

With both self‐assembled NP and membrane models established (see Section S6 for membrane morphological details), NP–membrane interactions were investigated through complementary approaches: (i) CG‐MD simulations with NPs initially positioned at a distance of approximately 1 nm from the membrane surface (Figure 1E) under internal human body condition (310 K and 1 bar; more details in Supporting Information and Sections S9) and (ii) experimental confocal microscopy of NP‐cell interactions (Section 2.3 and Figure 4). The analysis of these simulations, presented in the following sections, reveals how head and tail chemistry and membrane composition collectively dictate the fundamental interaction regimes—from stable surface adsorption to complete NP dissociation and amphiphile integration into the membrane—and how these molecular‐level behaviors correlate with experimentally observed cellular uptake and cytotoxicity patterns (Section 2.3). Through integrated analysis of simulation trajectories, experimental cellular uptake and toxicity analysis, thermodynamic calculations, and kinetic analysis, the following sections detail how NP‐membrane interactions depend on (i) amphiphile chemical structure, (ii) NP characteristics, and (iii) membrane composition.

### 2.2 Structure‐Driven NP‐Membrane Interaction Behavior

Simulation snapshots at 750 and 1500 ns in Figures 2 and 3 reveal three distinct NP‐membrane interaction regimes: FF‐tailed NPs (left column) remained stably adsorbed on the outer leaflet; HH NPs (right columns) dissociated rapidly into the membranes, particularly in cholesterol‐free systems (M0, M^*^0, DOPC); and HF‐tailed systems (middle column) displayed an intermediate behavior of partial, slow dissociation. Interestingly, these trends were consistent across both vesicular (G1) and wormlike (G2) architectures, despite their markedly different supramolecular structures. This finding challenges the established understanding that NP shape and curvature are the only dominant factors controlling membrane association, insertion, and disruption [57, 58, 59].

**FIGURE 2:**
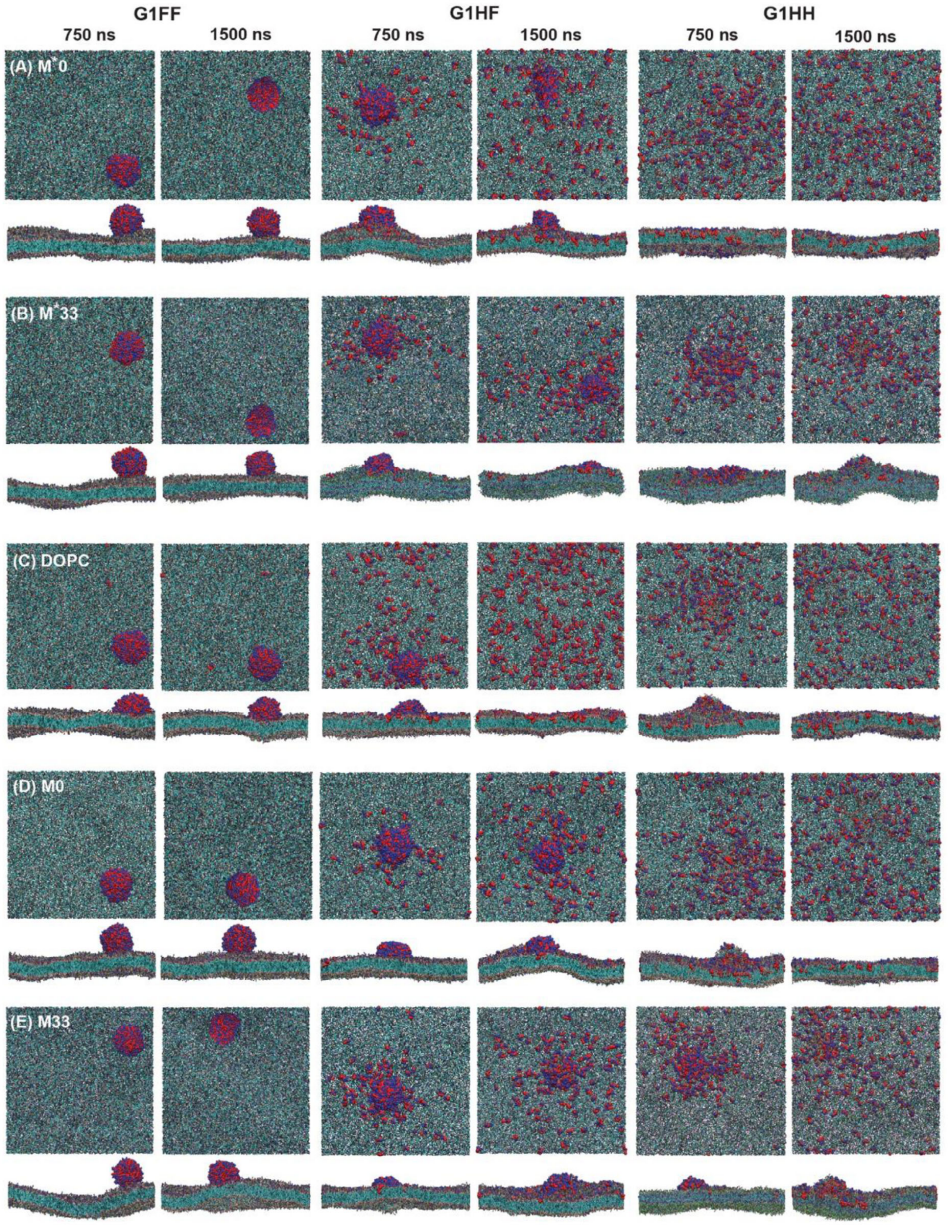
Temporal evolution of G1 NP‐membrane interactions. Top and side views of snapshots taken at 750 and 1500 ns for G1 nanoparticles interacting with (A) M^*^0, (B) M^*^33, (C) DOPC, (D) M0, and (E) M33 membranes. Each panel displays G1FF (left), G1HF (middle), and G1HH (right), illustrating the interaction modes, including surface adsorption (G1FF), partial and complete dissociation (G1HF and G1HH, respectively). To facilitate direct comparison of nanoparticle insertion and distribution across different membranes, hydrophilic heads and hydrophobic tails (including both H and F types) are consistently shown in blue and red, respectively.

**FIGURE 3:**
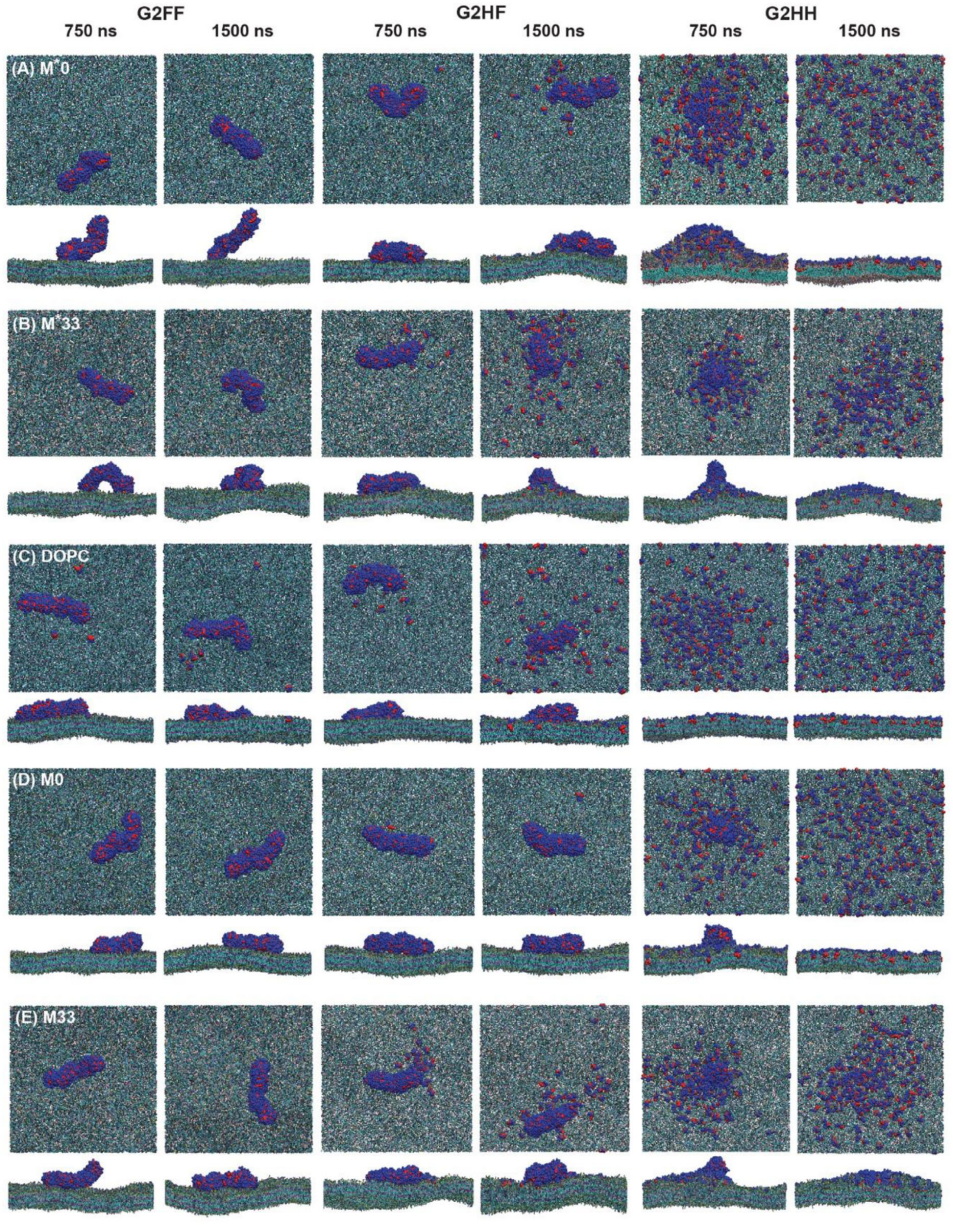
Temporal evolution of G2 NP‐membrane interactions. Top and side views of snapshots taken at 750 and 1500 ns for G2 nanoparticles interacting with (A) M^*^0, (B) M^*^33, (C) DOPC, (D) M0, and (E) M33 membranes. Each panel displays G2FF (left), G2HF (middle), and G2HH (right), illustrating the interaction modes, including surface adsorption (G2FF), partial and complete dissociation (G2HF and G2HH, respectively). To facilitate direct comparison of nanoparticle insertion and distribution across different membranes, hydrophilic heads and hydrophobic tails (including both H and F types) are consistently shown in blue and red, respectively.

Mechanistically, NP tail chemistry governs core stability and membrane compatibility. FF NPs resist dissociation due to strong fluorocarbon‐fluorocarbon cohesion, whereas HH NPs integrate readily into lipid bilayers due to their chemical similarity to the hydrophobic lipid tails, with dissociation rates enhanced in cholesterol‐free membranes. HF NPs display partial, composition‐ and headgroup‐dependent dissociation, with bulkier G2 headgroups reducing amphiphile disassembly. Importantly, two general principles emerge from these simulations: (i) global membrane properties dominate over leaflet asymmetry, as membrane symmetry (M vs. M^*^) has negligible effect on NP‐membrane interactions, and (ii) cholesterol content critically modulates interaction kinetics, particularly for HH and HF systems. This cholesterol‐dependent modulation arises from competing effects: enhanced membrane ordering increases kinetic barriers for insertion while simultaneously strengthening amphiphile‐membrane affinity once integration occurs — a duality quantitatively resolved through free energy analysis in Section 2.4. These mechanistic insights are corroborated by cellular uptake measurements (Section 2.3), which show no observable FF NP internalization but substantial HH NP molecular uptake.

### 2.3 Cellular Uptake Measurement

As a proof of concept to validate the simulation predictions, we visualized cellular uptake in HeLa cells using three amphiphile systems, G1FF, G1HH, and G1HF, spanning the full range of predicted membrane‐interaction behaviors from stable surface adsorption (G1FF) to rapid internalization (G1HH) (Figure 4), with G1HF representing the intermediate regime (Figure S29). G1FF and G1HH were selected as the mechanistic end‐members to probe the influence of tail chemistry — the most critical structural parameter identified in our simulations — on NP fate, and a representative semi‐fluorinated system (G1HF) was subsequently synthesized to test the predicted intermediate behavior directly, thereby establishing structure‐function relationships without the need for exhaustive experimental screening of all six amphiphile variants.

**FIGURE 4:**
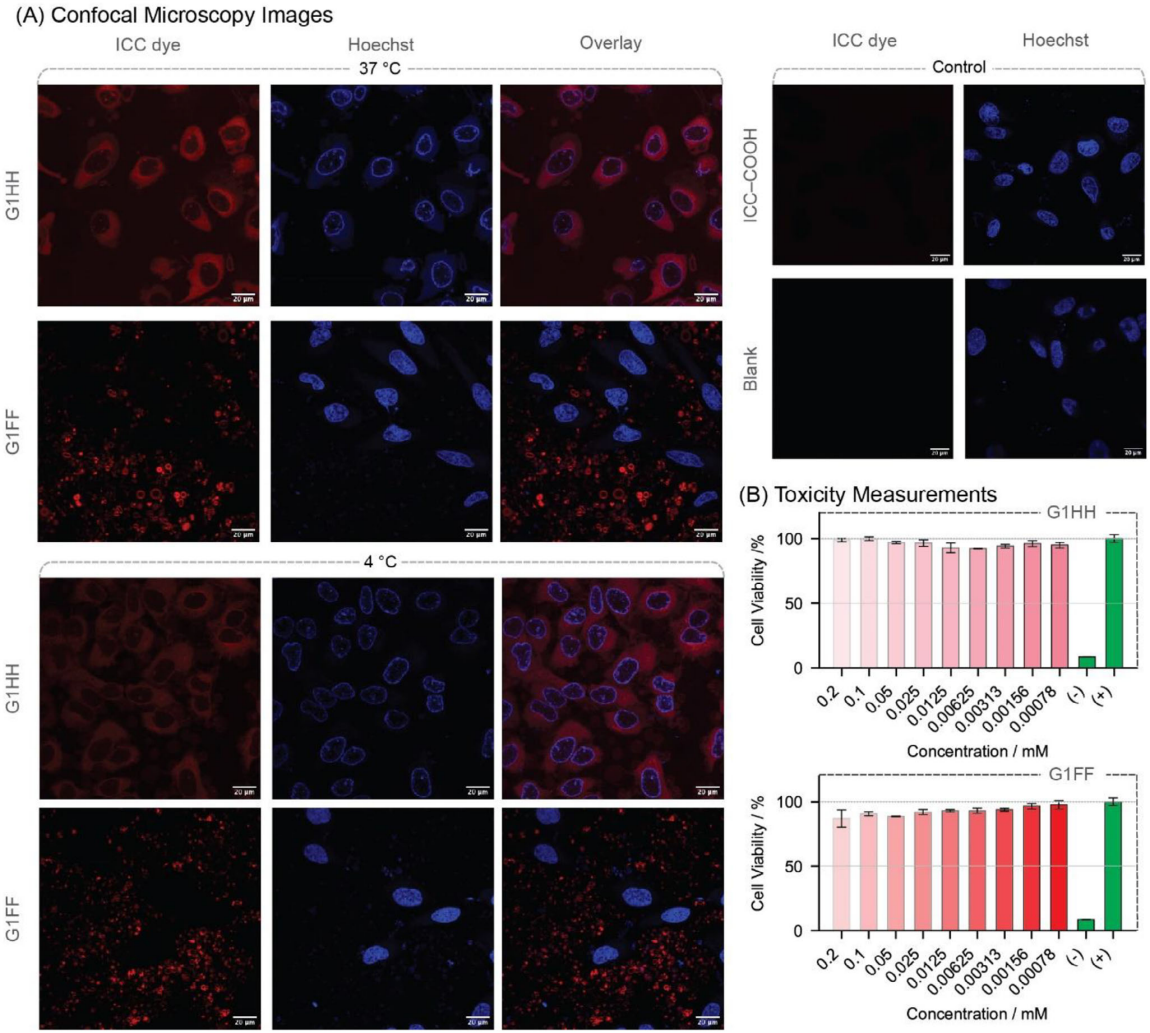
Experimental validation of structure‐dependent cellular uptake. (A) Confocal images of HeLa cells after 4 h incubation with 2 mol.% ICC‐labeled G1FF or G1HH. Red: ICC‐labeled amphiphiles; blue: Hoechst‐stained nuclei. Scale bar: 20 µm. (B) Cell viability (PrestoBlue assay) of HeLa cells after 24 h exposure to G1FF or G1HH. Untreated cells = 100% viability; 0.2% SDS = 0% viability. Mean ± SD, *n* = 3.

The amphiphiles were fluorescently labeled with a hydrophilic indocarbocyanine dye (ICC), enabling visualization in confocal microscopy as red fluorescence. Cell nuclei were counterstained with Hoechst dye, which appears blue (see Methods and Section S13 for synthetic and characterization details). Three amphiphile concentrations were tested: 0.4, 0.2, and 0.05 mm. To minimize the influence of dye labelling on self‐assembly behavior, only 2 mol.% of the amphiphiles were fluorescently labeled. Control samples included unbound ICC‐COOH at concentrations corresponding to 2 mol.% labeling (0.008, 0.004, and 0.001 mm), as well as a dye‐free and amphiphile‐free negative control in which only nuclear staining was applied. All confocal images are included in Figures S27 and S28 and the high concentration images are shown in Figure 4A.

Samples were incubated either at 37°C or 4°C. Incubation at 4°C suppresses active uptake mechanisms such as endocytosis, allowing only passive processes (e.g., diffusion), whereas at 37°C both passive and active transport may occur. Previous studies [60] have shown that fluorinated assemblies, which are generally more stable [29, 61], tend to be internalized via endocytosis, while hydrogenated assemblies more readily merge with the cell membrane and are internalized predominantly by passive diffusion.

Qualitative analysis of the confocal images indicates distinct cellular interaction profiles across the three amphiphile systems (G1FF, G1HH, and G1HF) consistent with the simulation results. We hypothesize that the high structural stability of the fluorinated G1FF vesicles, as predicted computationally, inhibits their direct fusion with the cellular membrane. The intermolecular interactions maintaining the integrity of fluorinated vesicles appear stronger than their affinity for the membrane, in contrast to the hydrogenated G1HH assemblies. Large, intact vesicles were observed for G1FF and were consistently located away from the cells, supporting their high stability even in cellular proximity. Notably, this distribution persisted at 37°C, where endocytosis could have occurred. This may be due to the large vesicle size observed in the confocal images; additionally, the high overall fluorescence intensity at the tested concentrations may obscure any low‐level uptake of the fluorinated amphiphile.

At the opposite extreme, G1HH exhibited clear intracellular fluorescence with no large vesicles detected, consistent with efficient cellular uptake and supporting the hypothesis that hydrogenated assemblies readily interact with cells rather than persisting extracellularly as intact vesicles, thus ultimately being internalized by the cells.

Positioned between these two limiting behaviors, G1HF displayed a concentration‐dependent interaction profile that directly follows the predicted intermediate behavior of the semi‐fluorinated series: at high concentration (0.40 mm) G1HF remained largely as extracellular aggregates without measurable internalization, resembling the surface‐associated behavior of G1FF, whereas at low concentration (0.05 mm), where monomeric species predominate, intracellular fluorescence indicated uptake of individual amphiphiles, resembling G1HH; at 0.20 mm both modes coexisted (Figure S29). This can be interpreted as experimental evidence for the intermediate interaction regime predicted for the HF series in Section 2.2, positioned between the surface‐adsorbing FF and the readily internalized HH systems.

Together, the simulation and experimental results demonstrate that hydrophobic tail chemistry functions as a direct and tunable design parameter governing NP stability and interactions with cellular membranes. Modulating the fluorination of hydrophobic tails enables control over NP dissociation behavior and membrane affinity. Furthermore, both G1HH and G1FF exhibited no detectable cytotoxicity toward HeLa cells up to concentrations of 0.2 mm (Figure 4B), and, to assess biocompatibility beyond the cancer‐cell model, all three amphiphiles (G1HH, G1FF, and G1HF) were additionally evaluated against the normal, fibroblast‐like L929 cell line, where they showed good biocompatibility across the tested concentration range (Figure S22), indicating their suitability for further exploration in biomedical applications. These validation experiments were carried out on the HeLa cell lines used here as representative model systems. A systematic comparison of nanoparticle uptake and biocompatibility across cell types constitutes a natural direction for future work.

### 2.4 Thermodynamic Origins of NP‐Membrane Interaction Behavior

To quantitatively rationalize the interaction modes discussed in Section 2.2, free energy profiles governing amphiphile partitioning between aqueous‐NP and aqueous‐membrane environments were computed using steered molecular dynamics (SMD) simulations with umbrella sampling. To this end, two amphiphile‐pulling calculations were performed: (i) pulling of one amphiphile out from a NP in water (Figure 5A‐left), and (ii) pulling of one amphiphile out from a membrane in water (Figure 5A‐right). In case (i), the dissociation free energy (ΔG_nano_) reflects NP cohesive stability in water, while the membrane extraction free energy (ΔG_membrane_), case (ii), quantifies amphiphile‐bilayer affinity. From these, the transfer free energy (ΔG_transfer_ = ΔG_nano_—ΔG_membrane_) was defined as a new predictive metric quantifying the thermodynamic drive for amphiphiles to leave their NP and integrate into the cell membrane bilayer. Note that positive ΔG_nano_ reflcts the energy cost to extract an amphiphile from NP, while positive ΔG_membrane_ reflects the cost to extract it from the membrane; therefore, negative ΔG_transfer_ indicates that membrane insertion is thermodynamically favored over remaining in the NP upon NP‐Cell interaction. Crucially, this metric is calculable without direct NP‐membrane simulations, enabling more straightforward prediction of NP‐cell interaction behavior.

**FIGURE 5:**
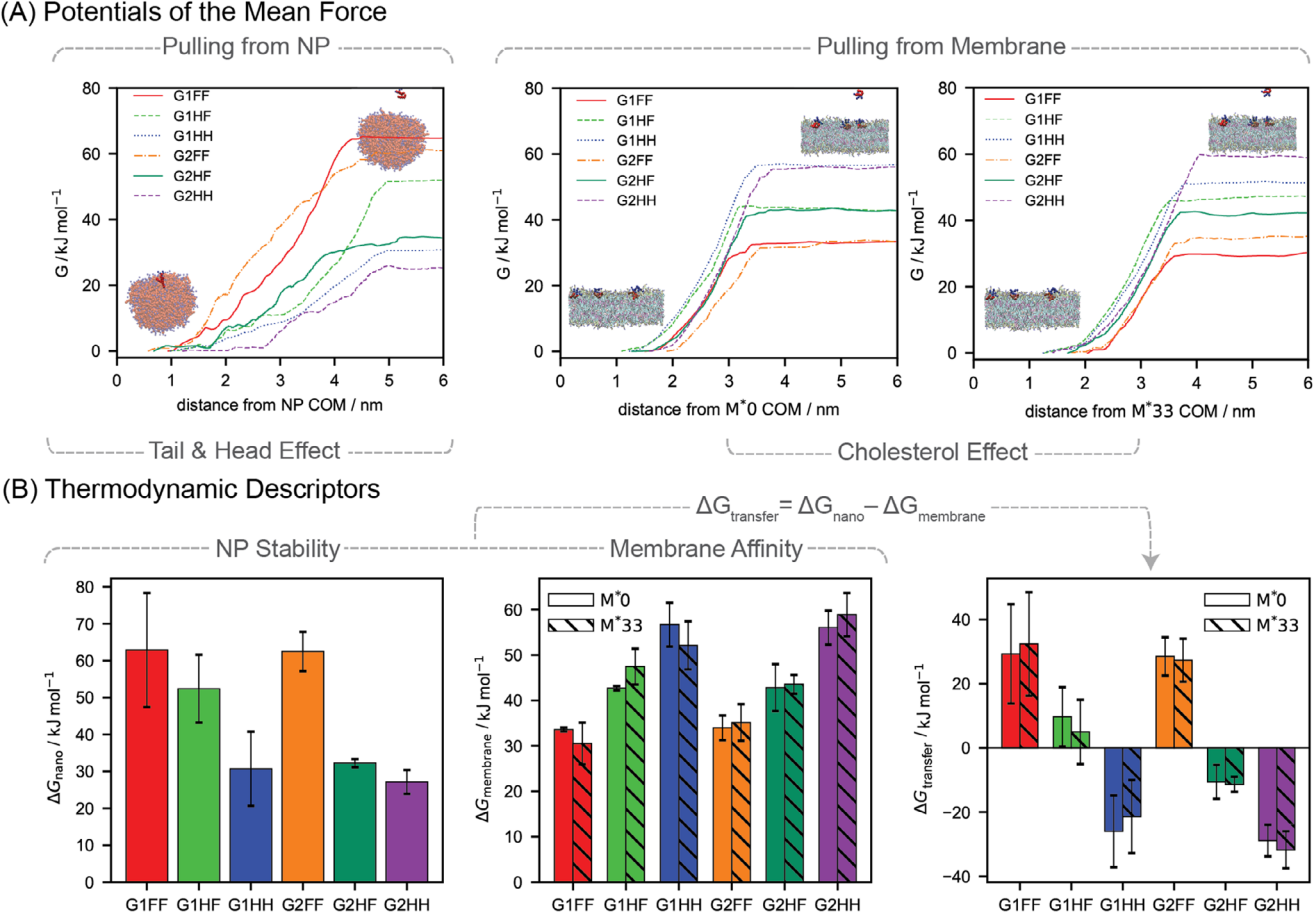
Thermodynamic framework for predicting NP‐membrane interactions. (A) Schematic illustration of steered molecular dynamics results for extracting amphiphiles from NPs (left) and membranes (right). (B) Nanoparticle dissociation free energy (ΔG_nano_) quantifying cohesive stability for all six nanoparticle types (left); membrane extraction free energy (ΔG_membrane_) quantifying bilayer affinity for amphiphiles in M^*^0 and M^*^33 membranes (middle) and transfer free energy (ΔG_transfer_) predicting spontaneous nanoparticle‐membrane association tendency across all systems (right, convergence analysis in Section S8). Plain bars represent cholesterol‐free membranes, while hatched bars represent cholesterol‐containing membranes in ΔG_membrane_ and ΔG_transfer_ diagrams.

Figure 5B‐left shows that FF‐tailed NPs exhibit the highest stability in water (ΔG_nano_), followed by HF and HH systems. This trend aligns with the significantly lower CMC measured for G1FF (0.16 ± 0.06 mm) compared to G1HH (0.96 ± 0.23 mm) (details in Section S12.1), confirming the strong cohesive stabilization imparted by fluorocarbon tails. Regarding the headgroup, G1 vesicles exhibit larger ΔG_nano_ than G2 worm‐like NPs across all tail types, consistent with (i) the more hydrophilic nature of G2 headgroup and (ii) G1's vesicular bilayered architecture providing deeper hydrophobic burial compared to G2's cylindrical geometry. Note that the larger standard deviations for G1 systems (compared to G2) in Figure 5B‐NP arise from the broader distribution of amphiphile positions within the vesicular bilayer, consistent with point (ii) above, as the bilayered architecture provides deeper hydrophobic burial, resulting in greater variation in the measured dissociation free energies.

Membrane extraction energies (ΔG_membrane_) reveal complementary insights according to Figure 5B‐middle. First, HH amphiphiles display higher ΔG_membrane_ than HF or FF ones, indicating that hydrocarbon tails form stronger interactions with membrane lipids. Second, unlike G1 amphiphiles which exhibit comparable ΔG_membrane_ values in both cholesterol‐free and cholesterol‐rich membranes, G2 amphiphiles display pronounced selectivity between these membrane environments. This generation‐dependent selectivity—absent in G1 but prominent in G2 – arises from the interplay between bulkier G2 headgroups and membrane ordering. In cholesterol‐rich membranes, the G2 headgroups preferentially remain partially exposed to the aqueous phase while maintaining hydrophobic tail insertion within the membrane core. This configuration minimizes local perturbations in the ordered lipid environment, preserving tight packing within cholesterol‐rich domains. considerably, FF amphiphiles show uniformly low ΔG_membrane_ across all membranes, confirming their poor lipid compatibility regardless of cholesterol content.

The transfer free energy (ΔG_transfer_) unifies these observations into quantitative predictions (Figure 5B‐right). HH systems exhibit strongly negative ΔG_transfer_ (−21 to −31 kJ/mol), predicting spontaneous membrane integration, which is in agreement with the extensive dissociation observed in simulations (Figures 2 and 3) and the extensive cellular uptake found experimentally (Figure 4). HF systems display negative or mildly positive ΔG_transfer_ (−11 to 5 kJ/mol) with considerably smaller absolute values compared to HH ones, consistent with their slower dissociation rates and membrane‐selective behavior where partial dissociation depends on membrane fluidity and headgroup steric constraints. FF systems possess positive ΔG_transfer_ (+27 to +32 kJ/mol), thermodynamically unfavorable membrane association—accounting for their persistent surface‐bound state (from simulation trajectories) and negligible cellular uptake (from cellular uptake experiments).

### 2.5 NP‐Membrane Interaction Kinetics

While thermodynamics determines equilibrium outcomes, biological relevance requires understanding temporal progression. Therefore, we quantified amphiphile (from NP to membrane) transfer dynamics by monitoring F_amphiphile_, i.e., the fraction of amphiphile beads in contact with lipid beads (within 0.6 nm), during the 1.5 µs simulations (Figure 6A). Furthermore, we calculated the transfer rate constants (k) from linear fits to the initial rising phase of contact‐time curves (Table 1). For HF systems, the initial rising phase spans nearly the entire simulation duration due to their slow dissociation kinetics. In contrast, for HH systems, which exhibit rapid membrane integration, the initial rising phase is confined to shorter time windows: approximately 200–300 ns for G1HH in M0/M^*^0, 400–500 ns for G2HH in M0/M^*^0, 300–400 ns for G1HH in M33/M^*^33, and 800–900 ns for G2HH in M33/M^*^33.

**FIGURE 6:**
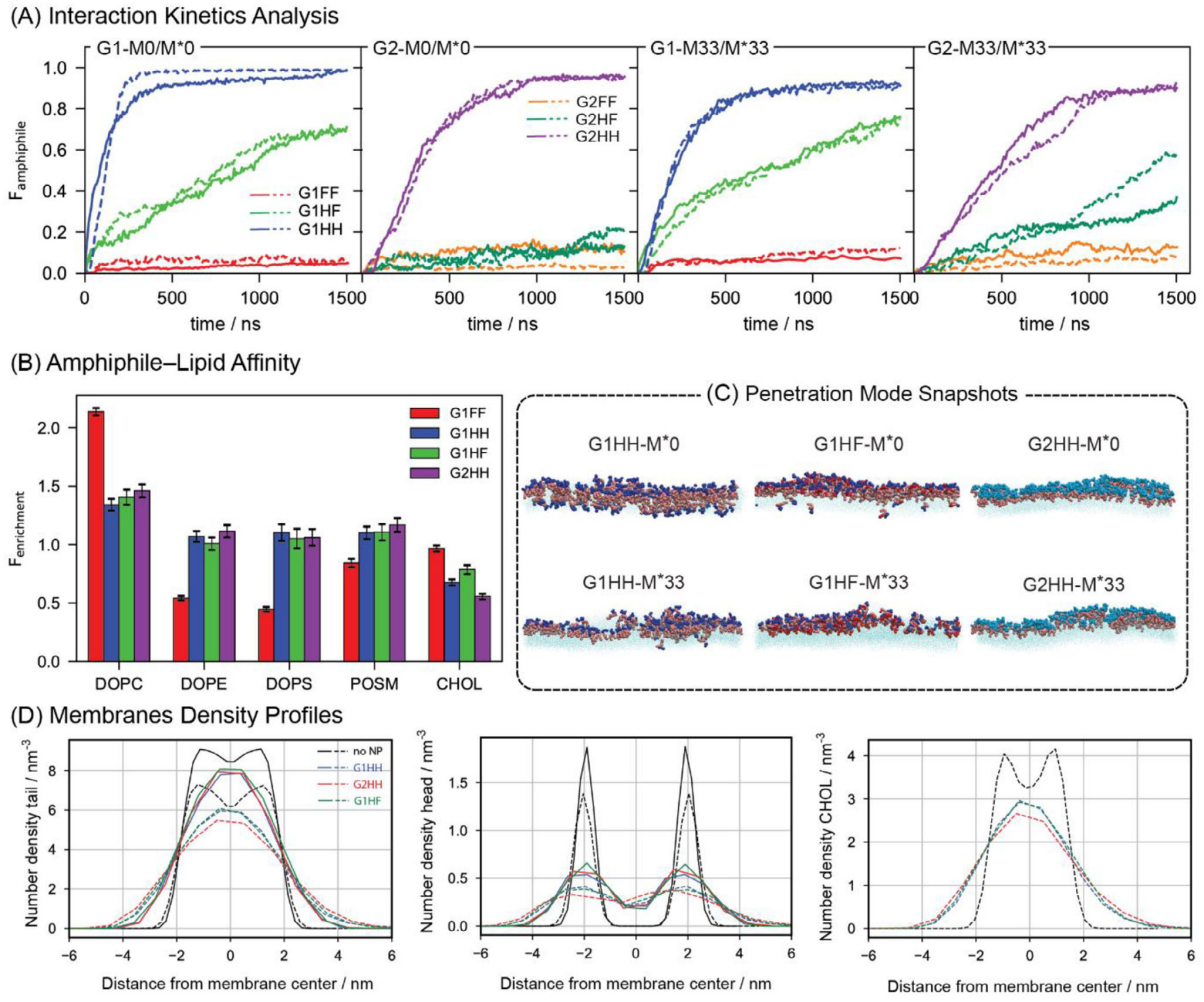
Kinetics, molecular selectivity, and structural reorganization of NP‐membrane interactions. (A) Temporal evolution of amphiphile‐membrane contacts fraction (F_amphiphile_) during 1.5 µs simulations for G1 and G2 systems interacting with cholesterol‐free (M0, M^*^0; solid and dashed lines) and cholesterol‐rich (M33, M^*^33; solid and dashed lines) membranes. (B) Fractional enrichment factors (F_enrichment_) quantifying amphiphile selectivity toward specific lipid species and cholesterol in cholesterol‐rich membranes, averaged over 3 µs. Values above 1 indicate preferential association, while values below 1 indicate depletion relative to random distribution. (C) Representative simulation snapshots at 3 µs showing distinct penetration modes and membrane localization patterns for G1HF, G1HH, and G2HH in cholesterol‐free (M*0) versus cholesterol‐rich (M^*^33) membranes (side views). (D) Number density profiles for lipid tails, headgroups, and cholesterol before and after NP interaction, averaged over the final 100 ns of 3 µs simulations for M^*^0 (solid lines) and M^*^33 (dashed lines) membranes.

**TABLE 1:** Transfer rate constants (k, ns^−^^1^) for amphiphile‐membrane association across different membrane compositions, extracted from linear fits to the initial rising phase of contact fraction curves. Higher k values indicate faster amphiphile‐membrane integration.

System	M0	M*0	M33	M*33
**G1HF**	0.098	0.109	0.087	0.101
**G1HH**	0.924	1.203	0.549	0.710
**G2HF**	0.012	0.017	0.027	0.063
**G2HH**	0.331	0.282	0.182	0.140

Regarding headgroup influence, G1 amphiphiles transfer faster than G2 across all conditions: k = 0.92–1.20 ns^−^^1^ for G1HH versus 0.28–0.33 ns^−^^1^ for G2HH in cholesterol‐free membranes (M0/M^*^0), and k = 0.10–0.11 ns^−^^1^ for G1HF versus 0.01–0.02 ns^−^^1^ for G2HF, confirming that smaller headgroups facilitate membrane insertion. Within each generation, transfer rates follow the thermodynamically predicted order HH > HF > FF, with HH systems exhibiting the fastest transfer and FF systems showing negligible transfer (k ≈ 0).

Cholesterol exerts a differential dampening effect on transfer kinetics. For G1HH, rates decrease from 0.92–1.20 ns^−^^1^ (M0/M^*^0) to 0.55–0.71 ns^−^^1^ (M33/M^*^33), corresponding to 40%–41% reduction. G1HF shows a more modest 7%–12% decrease (from 0.10–0.11 to 0.09–0.10 ns^−^^1^). Strikingly, G2HF exhibits the opposite trend: rates increase from 0.01–0.02 ns^−^^1^ (M0/M^*^0) to 0.03–0.06 ns^−^^1^ (M33/M^*^33), representing a 137%–264% enhancement. In contrast, G2HH experiences 45%–50% reduction (from 0.28–0.33 to 0.14–0.18 ns^−^^1^). This cholesterol‐dependent behavior reveals two distinct kinetic regimes: (i) Systems with strongly negative ΔG_transfer_ (HH amphiphiles) maintain substantial dissociation despite kinetic slowdown in ordered membranes, with a moderate cholesterol‐induced reduction (40%–41% for G1HH, 45%–50% for G2HH). Conversely, (ii) systems near the thermodynamic threshold (HF amphiphiles) exhibit generation‐dependent responses: G1HF shows minimal sensitivity to cholesterol (7%–12% reduction‐as seen in Figure 2), while G2HF uniquely accelerates in cholesterol‐rich membranes (observed in Figure 3), likely due to selective stabilization of partially inserted configurations that reduce the kinetic barrier for full integration. This programmable sensitivity enables membrane composition‐controlled drug delivery, where NP dissociation can be tuned by exploiting the interplay between headgroup architecture, tail chemistry, and lipid ordering. This computationally predicted intermediate behavior of the HF series is experimentally corroborated by the concentration‐dependent, intermediate cellular‐interaction profile of G1HF (Section 2.3). Leaflet symmetry (M vs. M^*^) produces negligible kinetic differences, consistent with the thermodynamic analysis.

To further investigate the amphiphile affinity toward specific lipids and cholesterol in the cell membranes, we computed fractional enrichment factors (F_enrichment_) [62] (Figure 6B):

(1) $$F_{\mathrm{enrichment}}=\frac{\frac{N_{\mathrm{lipid}-\mathrm{species}}}{N_{\mathrm{total}-\mathrm{lipid}}}}{\frac{P_{\mathrm{lipid}-\mathrm{species}}}{P_{\mathrm{total}-\mathrm{lipid}}}}$$

where N_lipid‐species_ is the number of contacts (i.e., similar definition as for F_amphiphile_) between amphiphile beads and a given lipid species, N_total‐lipid_ is the total amphiphile‐lipid contacts, P_lipid‐species_ is the number of molecules of that lipid type, and P_total‐lipid_ is the total lipid count.

Since some HF NPs did not fully dissociate within the initial 1.5 µs simulations, these were extended by an additional 1.5 µs to ensure full dissociation. For M^*^33 membranes, G1HH, G2HH, and G1HF display near‐random distribution across all lipids (F ≈ 1.01–1.46 for DOPC, DOPE, DOPS, POSM), confirming non‐selective lipid association following NP dissociation_._ The narrow enrichment range (F = 1.0–1.5) for dissociated HH and HF systems indicates that, once dissociated from the NP, amphiphiles distribute according to lipid availability rather than chemical preference. However, G1FF exhibits striking heterogeneity, showing high DOPC enrichment (F = 2.14) but depleted DOPE/DOPS/POSM contacts (F = 0.45–0.84). This apparent selectivity is an artifact of spatial restriction rather than chemical preference: positive ΔG_transfer_ confines G1FF to the outer leaflet where DOPC dominates, preventing deeper membrane integration. Therefore, G1FF enrichment values should not be interpreted as lipid‐specific affinity but rather as a consequence of its surface‐bound state.

In terms of cholesterol association, all systems exhibit cholesterol depletion (F < 1); however, fluorinated systems show considerably less depletion (F = 0.97 for G1FF, F = 0.79 for G1HF) compared to hydrocarbon systems (F = 0.68 for G1HH, F = 0.56 for G2HH). This trend suggests that fluorine–cholesterol interactions provide relative stabilization at the membrane interface, contributing to the surface‐biased localization of fluorinated amphiphiles and their slower or restricted penetration in cholesterol‐enriched membranes.

Simulation snapshots after 3 µs reveal qualitatively distinct penetration modes for different amphiphiles (Figure 6C), highlighting that NP dissociation does not guarantee deep membrane penetration. In cholesterol‐free membranes, only G1HH achieves complete transmembrane integration with headgroups translocating across leaflets. Although G1HF and G2HH undergo NP dissociation and release individual amphiphiles, the released amphiphilic molecules remain surface‐associated rather than penetrating deeply. G1HF adopts stable interfacial alignment with partial tail insertion providing membrane anchoring, while G2HH is sterically hindered from deep penetration despite favorable tail‐lipid compatibility, most likely due to the higher hydrophilicity of the G2 headgroup. In cholesterol‐rich membranes, G1HH, G1HF, and G2HH all undergo complete dissociation; however, the released amphiphiles remain heterogeneously distributed within the bilayer, indicating that cholesterol's kinetic dampening not only delays disassembly but also restricts lateral diffusion and redistribution of the freed amphiphiles, preventing uniform dispersion.

### 2.6 Membrane Structural Perturbation Upon Amphiphile Association

To assess the structural changes of the membrane upon amphiphile association, we quantified membrane perturbations via the area per lipid (APL) (see Method for details) and bilayer thickness, averaged over the final 100 ns of a 3 µs simulations (Table 2). Given the negligible influence of leaflet symmetry (M vs. M^*^) on interaction thermodynamics and kinetics established in previous sections, structural perturbations are analyzed only in terms of cholesterol content.

**TABLE 2:** Structural properties of membranes after interaction with G1HH, G1HF and G2HH nanoparticles, including area per lipid (APL) for outer and inner leaflets, membrane thickness, and relative changes compared to equilibrated membranes (Table S6). Values averaged over the last 100 ns of 3 µs simulations. Relative changes: ((Post—Pre)/Pre) × 100%.

Nanoparticle	Membrane	APL outer (nm^2^)	APL inner (nm^2^)	Thickness (nm)	ΔAPL outer (%)	ΔAPL inner (%)	ΔThickness (%)
	M*0	0.709 ± 0.003	0.707 ± 0.002	3.871 ± 0.010	+7.91	+7.61	−1.48
**G1HH**	M*33	0.833 ± 0.002	0.820 ± 0.003	3.912 ± 0.026	+60.50	+55.59	−5.19
	DOPC	0.731 ± 0.002	0.727 ± 0.003	3.823 ± 0.011	+7.34	+6.91	−1.26
	M*0	0.700 ± 0.000	0.701 ± 0.000	3.872 ± 0.010	+6.54	+6.70	−1.45
**G2HH**	M*33	0.576 ± 0.004	0.528 ± 0.003	4.067 ± 0.009	+10.98	+0.19	−1.43
	DOPC	0.720 ± 0.003	0.721 ± 0.003	3.818 ± 0.012	+5.73	+6.03	−1.39
	M*0	0.696±0.000	0.690±0.000	3.853±0.000	+5.94	+5.02	−1.94
**G1HF**	M*33	0.567±0.000	0.541±0.000	3.809±0.000	+9.25	+2.66	−7.67
	DOPC	0.715±0.000	0.716±0.000	3.844±0.000	+4.99	+5.29	−0.72

G1HH induces the most severe, cholesterol‐dependent disruption. In a cholesterol‐rich membrane, outer and inner leaflet APLs increase by ∼60% and ∼56%, respectively, accompanied by ∼5% bilayer thinning. These large perturbations reflect complete amphiphile integration and membrane compression. The massive APL expansion from ∼0.52 to ∼0.83 nm^2^ slightly exceeds the typical range for stable bilayers (0.50–0.70 nm^2^) [63]. In contrast, cholesterol‐free membranes exhibit only minor changes (∼7%–8% APL increase in both leaflets; ∼1%–1.5% thinning), indicating that cholesterol paradoxically amplifies disruption despite slowing dissociation dynamics. Thermodynamically, this stems from cholesterol‐enhanced ΔG_membrane_ values, which strengthen HH–membrane affinity. Once insertion occurs, the tight packing within ordered cholesterol‐rich domains promotes extensive lipid rearrangement: Each inserted amphiphile must displace multiple tightly‐packed lipids to accommodate its cross‐sectional area, creating a larger local perturbation compared to fluid membranes where lipids redistribute more easily.

G2HH causes markedly milder perturbations. In cholesterol‐rich membranes, outer‐leaflet APL increases by only 11% while inner‐leaflet changes remain below 0.2%, with minimal thickness reductions (∼1.4%). Cholesterol‐free systems show similarly modest effects (∼6% APL increase in both leaflets). Despite comparable ΔG_transfer_ values to G1HH, disruption remains limited because the bulky G2 headgroups restrict penetration depth, confining structural deformation to the outer leaflet– a trend strikingly evident in the asymmetric perturbation profile where outer leaflet expansion (+11%) far exceeds inner leaflet changes (+0.2%). This indicates that the G2 headgroup functions as a molecular “anchor,” preventing transmembrane insertion while maintaining inner leaflet integrity and preserving the structural asymmetry essential for proper membrane function.

G1HF induces moderate, cholesterol‐selective disruption. In cholesterol‐free membranes, both leaflets show only minor APL increases (∼5%–6%) and slight thinning (∼1%–2%), consistent with surface alignment and minimal membrane integration. However, in cholesterol‐rich membranes, the outer APL increases moderately (+9.25%) with inner leaflet expansion limited to +2.66%, accompanied by notable thickness reduction (−7.67%). This cholesterol‐dependent enhancement reflects the fluorinated tail's differential interaction with cholesterol‐rich domains (F_enrichment_ = 0.79), which selectively facilitates partial insertion and local membrane remodeling. The intermediate disruption profile—more pronounced than G2HH but considerably milder than G1HH—confirms G1HF's interfacial localization mode where amphiphiles penetrate sufficiently to perturb outer leaflet packing without achieving full transmembrane integration. The preferential outer leaflet perturbation (9.25% vs. 2.66% inner leaflet) further supports this surface‐biased insertion mechanism.

Collectively, these structural perturbations reveal a clear hierarchy: G1HH induces severe, cholesterol‐amplified disruption (60% APL increase), G1HF causes moderate, cholesterol‐selective remodeling (9% outer leaflet expansion), and G2HH produces mild, outer leaflet‐confined changes (11% asymmetric perturbation)—validating that headgroup architecture and tail chemistry together determine the extent and spatial distribution of membrane disruption. This cholesterol‐dependent behavior reveals a dual mechanism: thermodynamically, cholesterol enhances ΔG_membrane_ (Figure 5B), strengthening amphiphile‐bilayer affinity; kinetically, it increases membrane order, raising insertion barriers (Table 1). For HH systems, this creates delayed dissociation (40%–50% slower transfer rates) followed by amplified disruption (+60% vs. +8% APL in cholesterol‐rich vs. cholesterol‐free membranes), whereas HF systems exhibit generation‐dependent responses to this dual effect.

Number density profiles (Figure 6D) quantify membrane structural disruption at the molecular level. Before interaction, membranes exhibit well‐defined bimodal distributions for lipid tails and cholesterol, with distinct headgroup peaks separated by zero‐density midplane regions—hallmarks of bilayer integrity. Following G1HH, G1HF, and G2HH integration, tail and cholesterol profiles merge into single broadened peaks, indicating complete loss of leaflet distinction through transmembrane penetration and interdigitation. Headgroup peaks remain separated but compress toward each other with elevated interleaflet density, reflecting interface deformation without full coalescence. These transformations confirm amphiphile‐induced disruption of membrane asymmetry, with tail insertion driving the most severe structural perturbations.

## 3 Conclusions

A combination of thermodynamic and kinetic analyses from MD simulations, validated against advanced experimental analysis and characterization, reveals a predictive design framework that is able to link amphiphile chemistry to biological fate. While nanoparticle geometry is recognized as an important factor influencing membrane interactions by dictating orientation, contact area, and energetic barriers, we find that amphiphile chemistry constitutes a distinct and governing design principle. This is demonstrated by oligoglycerol amphiphiles which self‐assemble into fundamentally different morphologies (vesicular vs. worm‐like) with similar hydrophobic tail composition, yet exhibit comparable interaction modes, showing that chemical composition can override geometric effects. To quantify this phenomenon, we introduce ΔG_transfer_, the free energy of transferring a single amphiphile from the nanoparticle to the cell membrane, as a universal predictive descriptor for nanoparticle‐membrane interactions. This parameter, obtained from separate free energy calculations on nanoparticle and membrane systems, eliminates the need to explicitly model the entire amphiphilic nanoparticle–membrane system, and its sign and magnitude together predict interaction modes and cellular uptake likelihood. The strong quantitative agreement observed across four independent validation levels supports this framework: (i) simulated interaction trajectories, (ii) computed free energies, (iii) calculated amphiphile transfer rates, and (iv) experimental cellular uptake measurements spanning all three interaction regimes, with G1FF, G1HH, and G1HF confirming surface adsorption, transmembrane internalization, and the intermediate mode, respectively.

Tail fluorination dictates the mode of nanoparticle–membrane interaction. Fully fluorinated tails minimize uptake, preserving membrane integrity for surface targeting; hydrocarbon tails enable transmembrane integration for rapid release. Hybrid tails confer programmability, with nanoparticles remaining intact at rigid, cholesterol‐rich membranes but dissociating at fluid ones, enabling environment‐responsive design, a prediction now experimentally corroborated by the intermediate, concentration‐dependent cellular‐interaction profile of the synthesized G1HF system.

Headgroup effects are also shown to influence membrane interactions. First‐generation (G1) nanoparticles induce more structural disruption, whereas bulkier second‐generation (G2) headgroups sterically hinder deep amphiphile insertion while retaining membrane engagement. G2 thus enables interactions without severe bilayer disruption.

Cholesterol is able to rigidify the membrane, slowing amphiphile insertion and raising energy barriers. This delays hybrid nanoparticle dissociation, promoting cholesterol‐selective retention and limiting lateral diffusion, leading to heterogeneous bilayer distribution. For fully hydrocarbon nanoparticles, cholesterol slows insertion but amplifies membrane disruption through enhanced lipid rearrangement in ordered domains.

We note that the present models employ protein‐ and glycan‐free lipid bilayers, the established starting point for mechanistic NP–membrane studies, chosen here to isolate the molecular‐level amphiphile–lipid thermodynamics that govern the interaction regime. Real plasma membranes additionally incorporate a glycocalyx and transmembrane proteins, and, under physiological conditions, nanoparticles themselves acquire a protein corona from serum proteins prior to membrane contact. These factors primarily modulate the kinetics of NP–membrane encounter and subsequent trafficking rather than reversing the intrinsic partitioning thermodynamics captured by ΔG_transfer_. The ΔG_transfer_ descriptor is therefore expected to remain directionally valid in more complex environments, with quantitative refinement of absolute rate constants under explicit transmembrane‐protein inclusions—now accessible through the extended Martini 3 protein library—identified as a natural next step.

Our integrated approach directly links amphiphile chemical structure to nano–bio interactions, providing a unique predictive framework for the design of self‐assembled nanoparticles with controlled biological outcomes. By revealing how tail chemistry, headgroup size, and membrane composition collectively govern uptake, dissociation, and membrane disruption, our findings enable the rational engineering of nanoparticles for specific biotechnological applications—this includes targeted drug delivery, membrane‐selective therapeutics, or responsive drug release. These principles translate directly into design rules: fully fluorinated tails (ΔG_transfer_ +27 to +32 kJ/mol) yield surface‐targeting vectors for membrane‐confined delivery without internalization; hydrocarbon tails (ΔG_transfer_ −21 to −31 kJ/mol, k = 0.92–1.20 ns^−^^1^) provide rapid‐release intracellular carriers analogous to fusogenic lipid nanoparticles; and semi‐fluorinated G2HF systems, operating near the thermodynamic threshold with 137%–264% cholesterol‐gated rate acceleration, constitute a molecular blueprint for cancer‐selective, composition‐triggered release that exploits the elevated cholesterol of tumor membranes without external stimuli or targeting ligands. Moreover, the ΔG_transfer_ descriptor offers a practical, generalizable tool to predict nanoparticle behavior without needing to model entire nanoparticle–cell systems, thereby providing a route to accelerate high‐throughput design of biointeractive nanomaterials and guiding the development of safer, more effective nanomedicines.

## 4 Method

### 4.1 Simulation Workflow

The computational workflow comprised the following steps: (i) atomistic molecular dynamics (MD) simulations to parameterize coarse‐grained (CG) models; (ii) CG‐MD simulations to drive amphiphile self‐assembly into nanoparticles (NPs) in explicit water; (iii) CG‐MD simulations to equilibrate membrane models; (iv) steered MD (SMD) with umbrella sampling to compute potential of mean force (PMF) profiles and derive free energies (ΔG_nano_, ΔG_membrane_, ΔG_transfer_); (v) CG‐MD simulations of NP‐membrane interactions to quantify kinetics and structural perturbations (details in Sections S1–S9; parameters in Table S1; all simulation files required to reproduce the data presented in this paper, along with detailed README instructions, are available at ref. github.com/HMakkiMD/Nano_Bio).

### 4.2 Force Fields and Software

All simulations were performed using GROMACS 2022 [64], and molecular structures were visualized with VMD [65]. Atomistic simulations employed the CHARMM36 force field [66, 67], with systems prepared via CHARMM‐GUI [68, 69, 70]. Coarse‐grained (CG) simulations used the Martini 3 force field [49, 50]. Further details, including the bead typing, non‐bonded, and bonded parameters are provided in the Supporting Information and Section S4.

### 4.3 Atomistic Simulations

Bulk systems containing 100 amphiphile molecules in a ∼20 nm cubic water box were energy‐minimized, NVT‐equilibrated at 500 K, NPT‐equilibrated at 500 K/1 bar for 5 ns, cooled to 310 K at 0.04 K/ps, and finally NPT‐equilibrated at 310 K/1 bar for 100 ns. Convergence was confirmed by stabilization of density and radius of gyration (details in Section S3).

### 4.4 CG Parameterization and Validation

Atomistic‐to‐CG mapping followed the standard Martini scheme (2–4 heavy atoms per bead); bonded potentials were derived from atomistic trajectory distributions via Boltzmann inversion and fitted to harmonic forms (Equations S1–S3; details in Section S4; parameters in Table S2). Initial validation showed <7% deviation in bulk density and radius of gyration compared to atomistic results (Table S3). For the perfluorocarbon segments, the default Martini 3X4e bead does not reproduce perfluorocarbon thermodynamics across polar and nonpolar environments; we therefore employed a modified X4e bead in which only the Lennard‐Jones ε values of the cross‐interactions were adjusted (σ fixed), following the Martini 3 bead‐size philosophy. The full parameterization protocol, target observables, and validation against independent properties (excess free energy of mixing, miscibility, excess volume/enthalpy, atomistic reverse‐mapping comparison, self‐assembly morphology, structural validation, and CMC ranking) are detailed in the expanded Section S4; all parameter files are available in the associated repository.

### 4.5 NP Self‐Assembly

CG‐MD simulations were conducted at ∼20 mm amphiphile concentration in explicit Martini water, starting from random initial placement (bead‐matched systems across all amphiphiles, Table S4). Systems underwent energy minimization, NVT equilibration, and NPT production at 310 K/1 bar until a single NP formed (∼1350 ns). Convergence was monitored via a plateau in solvent‐accessible surface area (Figure S4; details in Section S5).

### 4.6 Reverse‐Mapped NP Equilibration

CG NP structures were back‐mapped to atomistic resolution using the Backward method [71], followed by two‐stage energy minimization, progressive NVT relaxation with increasing time steps (0.2–2.0 fs), and NPT equilibration at 310 K/1 bar for 100 ns. Equilibration was assessed via RMSD stabilization (∼2 × radius of gyration) and structural snapshots (Figures S8 and S9; details in Section S7). Final validation confirmed <7% deviation in bilayer thickness (Table S8), overall morphology (Figure S8), and local curvature (Figure S10), with good agreement in radial distribution functions and coordination numbers (Figures S11 and S12).

### 4.7 Membrane Construction and Equilibration

Simulations employed DOPC/DOPS/DOPE/PSM lipids with 0–33 mol.% cholesterol at physiological conditions (310 K, 1 bar). Membrane proteins and pH‐dependent protonation were excluded to isolate amphiphile chemistry effects on lipid interactions. Lipid bilayers (M0, M33, M^*^0, M^*^33, DOPC) were constructed using CHARMM‐GUI Martini Membrane Builder with specified compositions and asymmetry (Table S5). Systems were solvated, neutralized with Na^+^ ions, energy‐minimized, NVT‐equilibrated at 310 K, and NPT‐equilibrated at 310 K/1 bar for 900 ns. Membrane properties—area per lipid (APL), bilayer thickness, and lipid order parameter P_2—_were calculated from the final 100 ns (Figures S5–S7 and Table S6; details in Section S6).

### 4.8 NP‐Membrane Interactions

Equilibrated NPs were placed ∼6 nm above the bilayer surface, position‐restrained during initial equilibration, gently pulled to ∼1 nm separation, and then simulated under unrestrained NPT conditions at 310 K/1 bar for 1.5 µs (extended to 3 µs total for special systems; details in Section S9). Amphiphile‐membrane contacts (cutoff ≤0.6 nm) were used to compute contact fraction (F_amphiphile_) and lipid‐specific enrichment factors (F_enrichment_, Equation (1)).

### 4.9 SMD and PMF Calculations

Center‐of‐mass pulling of individual amphiphiles was performed at 0.001 nm/ps with a spring constant of 1000 kJ/mol·nm^2^; two to four independent replicates were run per NP type. PMFs were reconstructed using the weighted histogram analysis method (WHAM) with convergence verified (details in Section S8). NP dissociation free energy (ΔG_nano_) was obtained from pulling within the NP; membrane extraction free energy (ΔG_membrane_) from pulling out of the bilayer.

### 4.10 Cryo‐TEM

Aqueous solutions (7 mm) of G1HH, G1HF and G1FF were prepared and vitrified using a Vitrobot Mark IV (20°C, 100% humidity, 4 µL sample applied to 60 s plasma‐treated Quantifoil grids, blot force −11, 1 s drain time). Imaging was performed on a Talos Arctica microscope (200 kV, defocus −4 µm, magnification 28 000×, Falcon 3CE direct electron detector); scale bars were added using Fiji software (details in Section S12.1).

### 4.11 Cellular Uptake

HeLa cancer cell lines were seeded at 5×10^4^ cells/well and cultured overnight. Cells were incubated for 4 h with either ICC‐labeled amphiphiles (below and above CMC, 0.05 mm or 0.2 mm respectively; 1.3 and 3.3 mol.% ICC for G1FF and G1HH respectively), or control free dye samples (0.0025 mM or 0.01 mm) or no ICC dye (blank). Nuclei were counterstained with 1 µg/mL Hoechst 33342. Confocal imaging was performed using a Leica SP8 CLSM (63× oil immersion objective, λex 550 nm for ICC, 405 nm for Hoechst). The same settings were used for all dye concentrations. Quantification involved measuring cytoplasmic red fluorescence intensity per unit area (3 images per condition, 3–5 cells each, excluding aggregates; details in Section S13). The G1HF system was examined identically at 0.40, 0.20, and 0.05 mm (37°C), with confocal images provided in Figure S29.

## Conflicts of Interest

The authors declare no conflicts of interest.

## Supporting information




**Supporting File**: smll75376‐sup‐0001‐SuppMat.pdf.

## Data Availability

All the simulation input data, including structure, force field, and run files, for (i) nanoparticle self‐assembly simulations, (ii) nanoparticle‐membrane interaction simulations, (iii) atomistic reverse‐mapped models, and examples of output models for each category are provided in github.com/HMakkiMD/Nano_Bio.
