# Supplementary material for: Predictive Amphiphile Design Enables Control of Self‐Assembled Nanoparticle‐Cell Membrane Interactions
Source: Small. 2026 Aug 22;22(60):e75376. doi: 10.1002/smll.75376 (PMC13711464; doi:10.1002/smll.75376)
Supplement: Supplementary file 1 [file SMLL-22-e75376-s001.pdf]

Supporting Information for:

Predictive Amphiphile Design Enables Control of Self-Assembled Nanoparticle-Cell Membrane Interactions

Mahsa Nami[‡a], Tiffany Guitton-Spassky[‡b], Farhad Sharif [a*], Natalie Hanheiser [b], Riensqia Saudale,[b] Jannik Gabriel [b], Aarti [b], Alireza Mirzaalipour [c], MohammadJavad Hafezi [a], Boris Schade [d], Abhishek Kumar Singh [b,e*], Rainer Haag [b], Roland R. Netz [f], Hesam Makki [g,h*]

[a] Department of Polymer and Color Engineering, Amirkabir University of Technology, 424 Hafez Avenue, Tehran 59163-4311, Iran.

[b] Institut für Chemie und Biochemie, Organische Chemie, Freie Universität Berlin, Takustraße 3, 14195 Berlin, Germany.

[c] KU Leuven, Department of Chemical Engineering, Soft Matter, Rheology and Technology, 3001 Leuven, Belgium.

[d] Institut für Chemie und Biochemie, Forschungszentrum für Elektronenmikroskopie, Freie Universität Berlin, Fabeckstraße 36a, 14195 Berlin, Germany

[e] School of Biosciences, Chanakya University, Bengaluru, India

[f] Department of Physics, Freie Universitätät Berlin, Arnimallee 14, 14195 Berlin, Germany.

[g] Department of Chemistry and Materials Innovation Factory, University of Liverpool, L69 7ZD Liverpool, United Kingdom.

[h] Department of Chemical Engineering, University of Bath, Bath, BA2 7AY, United Kingdom.

* Corresponding authors: Prof. Farhad Sharif (sharif@aut.ac.ir), Dr. Abhishek Kumar Singh (abhikmc@zedat.fu-berlin.de), and Dr. Hesam Makki (hm2518@bath.ac.uk)

‡**Mahsa Nami and Tiffany Guitton-Spassky share the first authorship.**

Contents

Part A: Modelling ***3***

S1. Simulation Methodology Overview 3

S2. Amphiphilic Molecules 4

S3. Atomistic Simulation Details 4

S4. Coarse-Grained (CG) Parameterization and Validation 4

S5. NP Self-Assembly 9

S6. Lipid Membrane Compositions and Equilibration 9

S7. self-assembled Structures and their validation 14

S8. Steered Molecular Dynamics Simulations and Convergence Analysis 19

S9. NP-Membrane Interactions 20

Part B: Experimental ***21***

S10. General synthetic and analytical methods 21

S11. Synthetic procedure and characterisation data 21

S12. Characterisation of amphiphile self-assembly in water 26

S12.1. Determination of amphiphile CMC *31*

S13. Membrane interaction experiments 33

S14. Copies of NMR spectra 39

References ***53***

Part A: Modelling

S1. Simulation Methodology Overview

All simulations, including both atomistic and CG models, were conducted using GROMACS 2022 [1], with visualizations generated via VMD [2].The atomistic simulations utilized the CHARMM36 force field [3, 4] and the CHARMM-GUI web interface [5-7], while CG simulations were performed using the Martini 3 force field. **TableS1** presents the detailed parameters for all simulations conducted across various stages, including energy minimization, and MD simulations under NVT and NPT conditions for amphiphile equilibration (**section S3**), nanoparticle (NP) self-assembly (**section S4**), reverse-mapped NP equilibration (**section S7**), membrane equilibration (**section S6**), and NP-membrane interaction studies (**section S9**).

Table S1. MD simulation parameters for amphiphile equilibration, NP self-assembly, reverse-mapped NP equilibration, membrane equilibration, and NP-nembrane interaction.

MD Simulation Parameter	Amphiphile Equilibration (Atomistic)	Reverse-Mapped NP Equilibration (Atomistic)	NP Self-Assembly (CG)	Membrane Equilibration (CG)	NP-Membrane Interaction (CG)
**Energy Minimization**					
Integrator	steep	-	steep	steep	steep
Number of steps	50000	-	250000	250000	250000
Time step (ps)	0.01	-	0.02	0.02	0.02
Cutoff scheme	Verlet	-	Verlet	Verlet	Verlet
Electrostatics	PME	-	Reaction-field	Reaction-field	Reaction-field
vdW cutoff (nm)	1.0	-	1.1	1.1	1.1
Constraints	none	-	none	none	none
**NVT Ensemble**					
Integrator	md	md	md	md	md
Number of steps	1000000	2000000	4000000	4000000	4000000
Time step (ps)	0.002	0.002	0.015	0.015	0.015
Cutoff scheme	Verlet	Verlet	Verlet	Verlet	Verlet
Electrostatics	PME	PME	Reaction-field	Reaction-field	Reaction-field
vdW cutoff (nm)	1.0	1.0	1.1	1.1	1.1
Constraints	all-bonds	all-bonds	none	none	none
Temperature (K)	310	310	310	310	310
Thermostat	V-rescale [8]	V-rescale	V-rescale	V-rescale	V-rescale
taut (ps)	0.1	0.1	1.0	1.0	1.0
**NPT Ensemble**					
Integrator	md	md	md	md	md
Number of steps	50000000	50000000	90000000	60000000	100000000
Time step (ps)	0.002	0.002	0.015	0.015	0.015
Cutoff scheme	Verlet	Verlet	Verlet	Verlet	Verlet
Electrostatics	PME	PME	Reaction-field	Reaction-field	Reaction-field
vdW cutoff (nm)	1.0	1.0	1.1	1.1	1.1
Constraints	all-bonds	all-bonds	none	none	none
Pcoupltype	isotropic	isotropic	isotropic	Semiisotropic	Semiisotropic
Barostat	C-rescale [9]	C-rescale	C-rescale	Parrinello-Rahman [10]	Parrinello-Rahman
taup (ps)	2.0	2.0	6.0	12.0	12.0
Pressure (bar)	1.0	1.0	1.0	1.0 1.0	1.0 1.0
Compressibility	4.5e-5	4.5e-5	4.5e-5	3e-4 3e-4	3e-4 3e-4

S2. Amphiphilic Molecules

Figure S1 illustrates the molecular structure these amphiphiles.

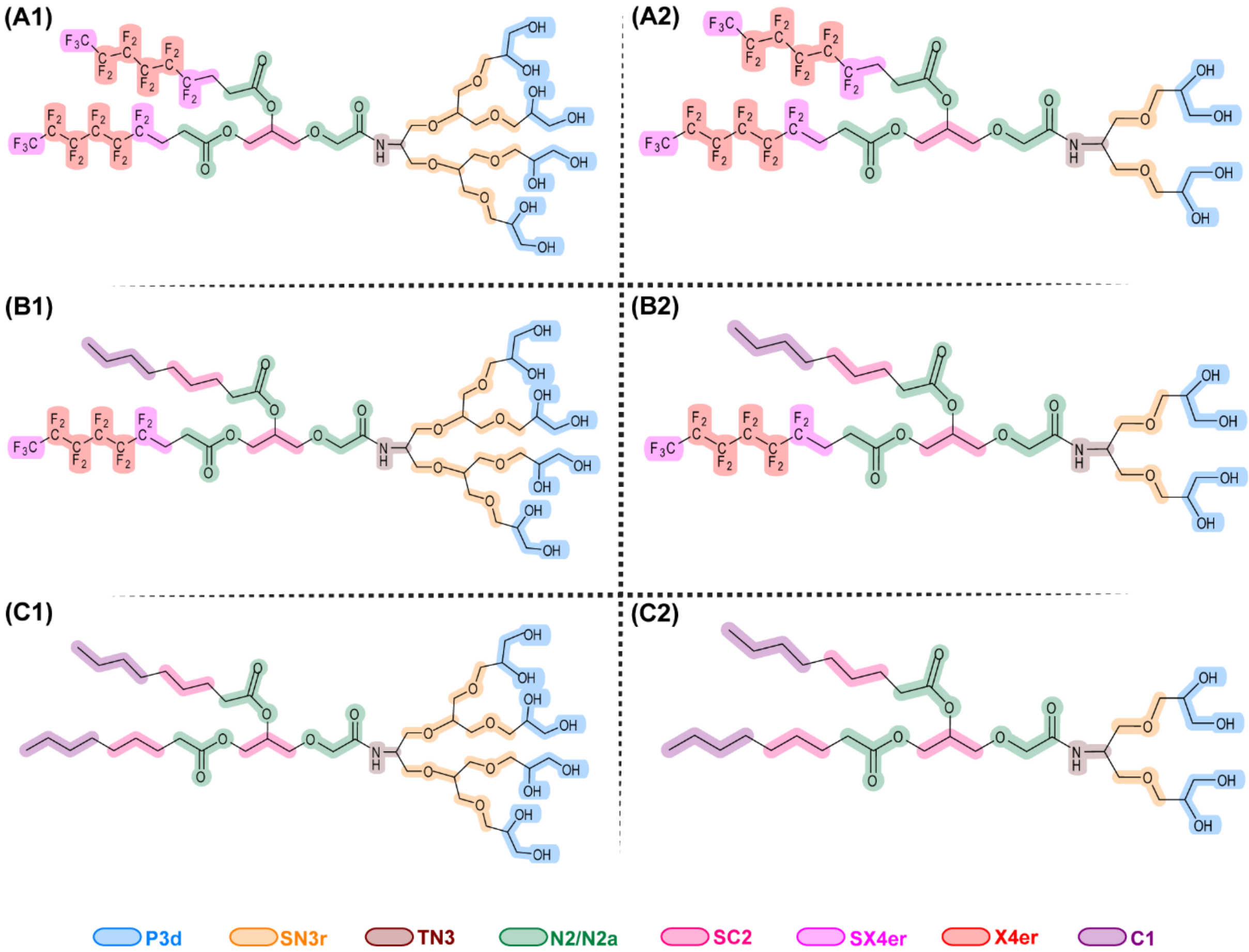

Figure S1. Molecular structures and coarse-grained (CG) mappings of the amphiphiles: (A1) G1FF, (B1) G1HF, (C1) G1HH, (A2) G2FF, (B2) G2HF, (C2) G2HH.

S3. Atomistic Simulation Details

Atomistic MD simulations were employed to equilibrate bulk systems of each amphiphilic molecule, providing reference data for CG parameterization. Each system comprised 100 amphiphile molecules randomly distributed within a 20 nm cubic simulation box. The simulation protocol initiated with energy minimization, followed by an NVT equilibration at 500 K. Subsequently, a 5 ns NPT simulation was conducted at 500 K and 1 bar. The system was then gradually cooled from 500 K to 310 K at a rate of 0.04 K ps^{-1}. Final equilibration was performed under NPT conditions at 310 K and 1 bar for 100 ns. Convergence was verified by monitoring the time evolution of the bulk density and the radius of gyration of amphiphiles, both stabilizing at plateau values, confirming structural and thermodynamic equilibrium. Detailed simulation parameters are presented in **Table S1**.

S4. Coarse-Grained (CG) Parameterization and Validation

Equilibrated atomistic configurations were mapped to their CG representations in accordance with the standard Martini strategy, where each bead corresponds to the center of geometry (COG) of grouped atoms [11] and is classified as regular (4 heavy atoms), small (3 heavy atoms), or tiny (2 heavy atoms) [12]. The bead mapping scheme and corresponding bead types, each represented by a unique color, are illustrated in **Figure S1** for all amphiphiles.

The distance and angular distributions of the beads were mapped from the trajectory of atomistic MD simulations and then transformed into potential energy profiles via the Boltzmann inversion:

$$U = -\ln(p)kT \tag{S1}$$

where U is the potential, p is the probability distribution, k is Boltzmann's constant, and T is the absolute temperature.

Then, the obtained potentials were fitted to harmonic forms:

$$U_{bond} = \frac{1}{2} K_{bond} (l - l_0)^2 \tag{S2}$$

$$U_{angle} = \frac{1}{2} K_{angle} (\cos\theta - \cos\theta_0)^2 \tag{S3}$$

Figure S2 presents representative probability densities of bond lengths (panel A-C) and angle values (panel B-D) for G1FF from independent atomistic and CG simulations.

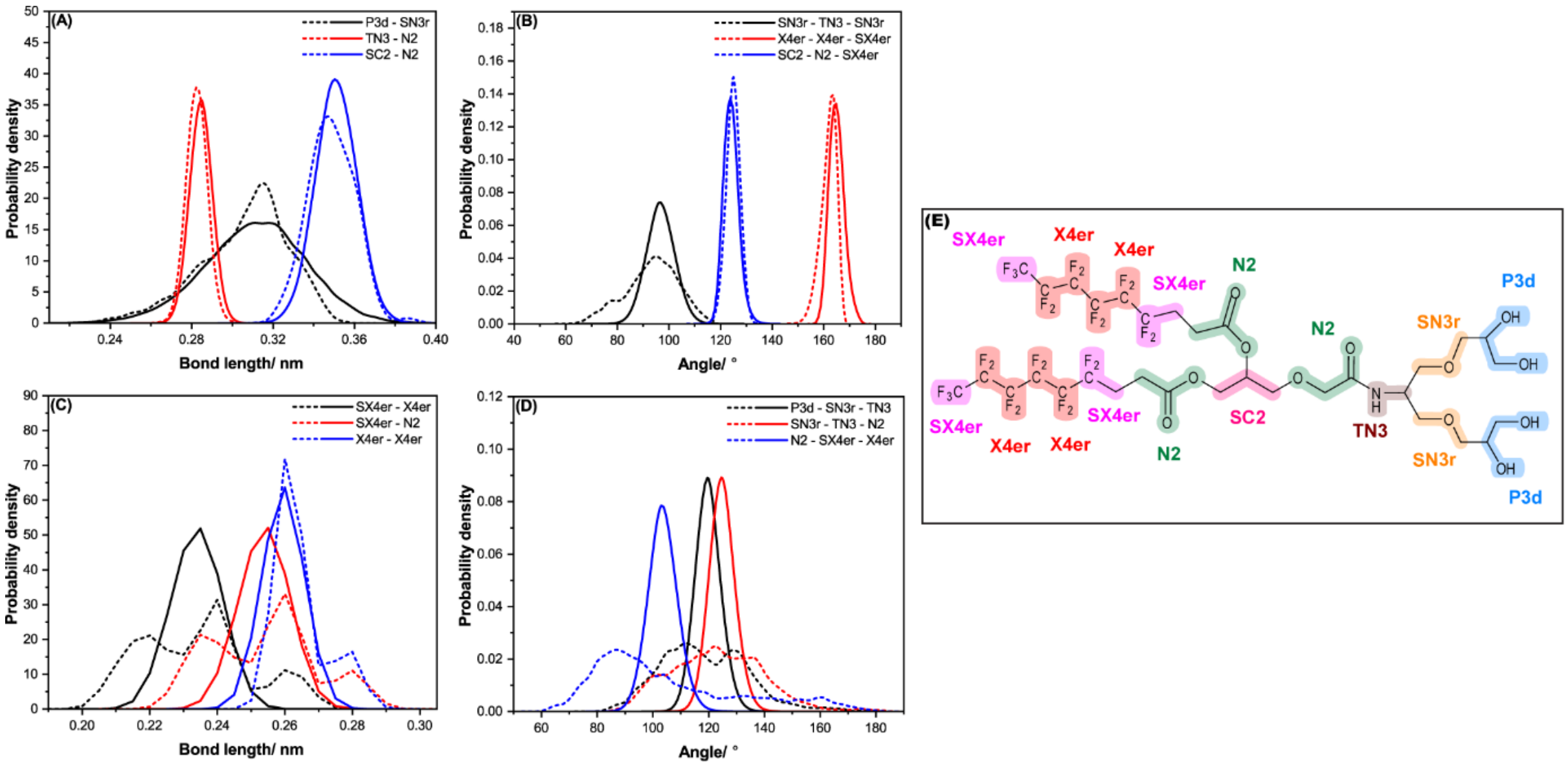

Figure S2. Representative probability density distributions of (A-C) bond lengths and (B-D) angles for G1FF system from atomistic (dashed-line) and CG (solid-line) simulations including the best (A-B), and the average/worst (C-D) matching ones, (E) mapping and bead types of G1FF.

The optimized bonded parameters for all bonds and angles appearing across all amphiphile systems are summarized in **Table S2**.

Table S2. Bond and angle parameters for the CG Martini model.

Bond Type	l_0 (nm)	K_{bond} (kJ/mol nm^{-2})
P3d-SN3r	0.32	6000
SN3r-TN3	0.25	26000
SN3r-SN3r	0.35	45000
TN3-N2/ N2a	0.285	80000
N2/N2a-SC2	0.33	60000
SC2-N2/N2a	0.35	30000
N2/N2a-SX4er	0.26	90000
SX4er-X4er	0.258	110000
X4er-X4er	0.255	170000
X4er-SX4er	0.235	175000
SC2-SC2	0.32	29000
SC2-C1	0.435	50000
**Angle Type**	**θ_0 (degree)**	**K_{angle} (kJ/mol)**
P3d-SN3r-TN3	120	680
SN3r-TN3-SN3r	96	100
P3d–SN3r–SN3r	120	680
SN3r-TN3-N2/N2a	125	700
TN3-N2/N2a -SC2	122	750
N2/N2a -SC2	100	600/420

SC2-N2/N2a-SX4er	122	650
N2/N2a -SX4er-X4er	114	900
SX4er-X4er-X4er	170	45000
X4er-X4er-SX4er	164	20000
SC2-N2a-C1	140	1200
N2a-SC2-SC2	135	650
SC2-SC2-C1	140	1200
P3d-SN3r-SN3r	120	680
SN3r-SN3r-SN3r	75	145
SN3r-SN3r-TN3	120	700/600
N2-SC2-N2	122	630

Bulk density and radius of gyration (R_g) were calculated for equilibrated atomistic and CG models, with values averaged over the last 5 ns. **Table S3** shows a good agreement, with differences below 7% – thereby confirming the structural fidelity of the CG model.

Table S3. Comparison of bulk densities and radii of gyration from atomistic and CG Martini simulations for the amphiphiles. Differences are calculated as ((CG - AA)/AA)*100%.

System	Density (kg/m³) Atomistic	Density (kg/m³) CG	Difference (%)	Rg (nm) Atomistic	Rg (nm) CG	Difference (%)
**G1FF**	1567	1480	−5.56%	0.655	0.659	+0.61%
**G2FF**	1417	1365	−3.67%	0.728	0.725	−0.41%
**G1HH**	1090	1092	+0.18%	0.623	0.621	−0.32%
**G2HH**	1122	1110	−1.07%	0.703	0.701	−0.28%
**G1HF**	1362	1438	+5.58%	0.650	0.653	+0.46%
**G2HF**	1318	1329	+0.84%	0.719	0.722	+0.42%

Fluorocarbon bead reparameterization. The standard Martini 3 force field employs X-class beads (e.g., X4e, SX4e) to represent halogenated moieties.[13] However, as established in our companion parameterization study,[14] the default X4e bead does not reproduce the thermodynamic behavior of perfluorocarbons across polar and nonpolar environments. Analogous limitations for fluorinated systems have been reported previously: Yan et al.[15] showed that tailored Martini 3 parameters are required to capture PFAS self-assembly in water, and Rashmi et al.[16] likewise noted the need for customized parameters in mixed fluorocarbon–hydrocarbon amphiphiles. For this reason, the perfluorocarbon segments of the present amphiphiles (G1FF, G2FF, and the fluorinated tail of the HF systems) were described using the modified X4e bead developed in ref 14.

Parameterization workflow. The reparameterization follows the standard Martini 3 protocol and modifies only the Lennard-Jones ε values of the cross-interactions, while keeping the bead sizes (σ) fixed, in accordance with the bead-size philosophy of Martini 3.[13] A key observation underlying the procedure is that the excess free energy of mixing of perfluorohexane with a range of solvents exhibits an approximately linear dependence on the Lennard-Jones ε parameter across eleven solvents spanning the full Martini 3 polarity range (from water and DMSO to octane and diethyl sulfide). This near-linear relationship enabled a simple, physically interpretable iterative fitting scheme: starting from the default Martini 3 interactions, ε values were gradually adjusted until both the magnitudes and the relative ordering of the excess free energies matched atomistic reference data obtained with the OPLS-AA force field.[17]

Validation. The final parameter set was validated against multiple independent observables that were not part of the fitting target:

(i) **Excess free energy of mixing.** Agreement with the OPLS-AA reference was recovered across all eleven solvent classes, with errors reduced from up to ~17 kJ/mol for the default Martini 3 X4e bead to generally within ~1 kJ/mol.

(ii) **Miscibility classification.** Contact-number analysis correctly classified systems as biphasic, partially miscible, or fully miscible, in agreement with atomistic simulations.

(iii) **Excess volume and mixing enthalpy.** For the perfluorohexane–octane mixture, both the excess molar volume and the mixing enthalpy showed improved agreement with experimental data[18, 19] relative to the default model.

(iv) **Atomistic reverse-mapping comparison**. To directly test whether the modified parameters improve fidelity to atomistic behavior, self-assembled nanoparticles obtained with the original (default) and with the modified Martini 3 parameter sets were reverse-mapped to atomistic resolution and compared against independent all-atom (OPLS-AA) simulations of the same systems (Figure S3). Nanoparticles obtained with the original Martini 3 parameters showed irregular, poorly phase-separated assemblies that deviated markedly from the atomistic reference, whereas those obtained with the modified parameters closely reproduced both the overall morphology and the internal hydrophobic-tail/hydrophilic-headgroup phase separation seen in the atomistic structures.

(v) **Self-assembly morphology.** For the fluorinated dendritic amphiphiles (G1FF and G2FF), the modified parameters reproduced self-assembled morphologies consistent with the all-atom reverse-mapped structures, with quantitative agreement in the mean-curvature analysis; by contrast, the default X4e parameters produced the non-physical aggregates shown in Figure S3.

(vi) **Structural validation.** Bulk density and radius of gyration across all six amphiphile systems agree within 7% between coarse-grained and atomistic simulations (Table S3), consistent with established Martini 3 benchmarks.[13]

(vii) **CMC ranking.** The experimental ordering of critical micelle concentrations is correctly reproduced, e.g., G1FF (0.16 ± 0.06 mM) < G1HH (0.96 ± 0.23 mM) (Section S12.1), consistent with the stronger cohesion imparted by fluorinated tails.

Scope and transferability. Because only cross-interactions are adjusted in a modular fashion, the modified perfluorocarbon bead is transferable to other amphiphilic systems containing CF_2 and CF_3 units, in line with Martini 3 design principles.[13] A comparable strategy — targeted refinement of cross-interactions — was employed in the Martini 3 cholesterol reparameterization.[20] The complete parameter set, target observables, and validation data are provided in the companion study[14] and its associated repository (github.com/HMakkiMD/Modified_Martini3_Fluorocarbon).

Figure S3 presents self-assembled structures of the fully fluorinated nanoparticles obtained with the original and modified Martini 3 parameters. For both G1FF (A1–A3) and G2FF (B1–B3), nanoparticles are shown using the original Martini 3 parameters (A1, B1), the modified parameters (A2, B2), and reverse-mapped all-atom (OPLS-AA) structures (A3, B3). Cross-sectional views are shown, with full nanoparticle renderings in the lower right corner of each panel. The modified parameters markedly improve agreement with the atomistic reference: both the overall shape (mean-curvature analysis) and the internal phase separation between hydrophobic tails and hydrophilic headgroups closely match the atomistic structures, whereas the original Martini parameters yield irregular, poorly phase-separated assemblies.

To further verify the reparameterization, we tracked whether an atomistic trajectory initiated from the original Martini 3 prediction spontaneously evolves toward the morphology obtained with the modified parameters. The G1FF nanoparticle predicted by the original Martini 3 parameters (panel C1) was reverse-mapped to atomistic resolution and relaxed with the OPLS-AA force field for 300 ns (panels C2–C4, corresponding to 100, 200, and 300 ns). Over this trajectory, the assembly progressively reorganized away from the irregular, poorly phase-separated structure of the original Martini prediction and toward the well-defined, internally phase-separated morphology independently obtained with the modified parameters (panel A2). This convergence is quantified in panel C5, which compares the mean-curvature (K_m) distributions of three atomistic structures: the

atomistic model derived from the modified parameters, and the atomistic models derived from the original parameters at 0 ns and at 300 ns of OPLS-AA relaxation. The K_m distribution of the original-parameter structure shifts steadily during relaxation and, by 300 ns, closely approaches that of the modified-parameter structure. Because an unbiased atomistic force field independently drives the original-parameter assembly toward the modified-parameter morphology, this analysis provides direct, force-field-level validation that the modified parameters — not the original ones — reproduce the physically correct self-assembled structure.

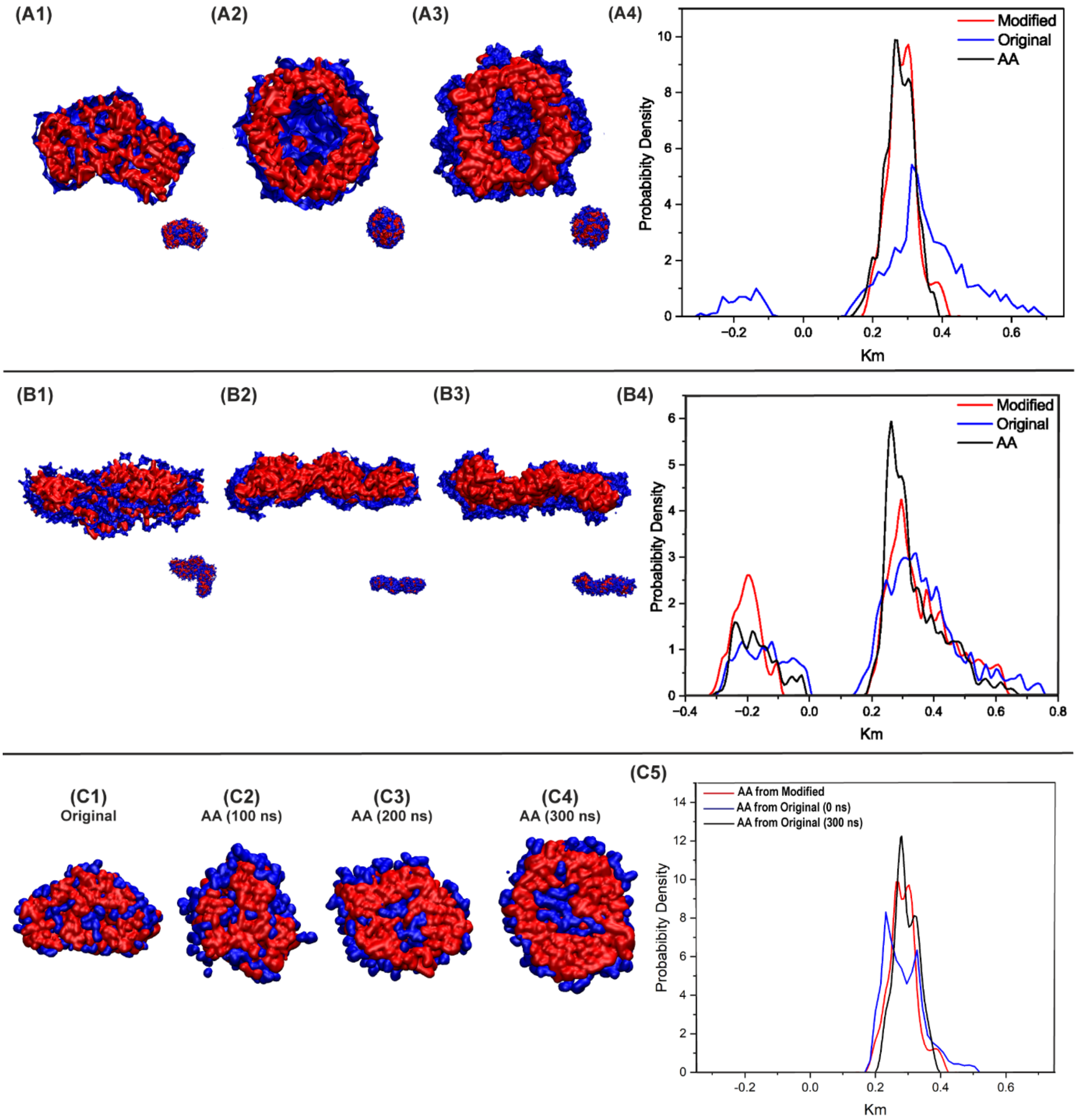

Figure S3. Comparison of self-assembled nanoparticles of the fluorinated dendritic amphiphiles G1FF (A) and G2FF (B) obtained with the original and modified Martini 3 parameters. (1) Original Martini 3 parameters, (2) modified Martini 3 parameters, (3) reverse-mapped all-atom (OPLS-AA) structure (in all panels hydrophobic tails are shown in red and hydrophilic heads in blue) and (4) mean-curvature (Km) distributions. (C) Atomistic (OPLS-AA) relaxation of the G1FF nanoparticle initiated from the original Martini 3 prediction: (C1) original Martini 3 nanoparticle; (C2–C4) reverse-mapped atomistic structure after 100, 200, and 300 ns of OPLS-AA relaxation; (C5) mean-curvature (Km) distributions for the atomistic structure obtained from the modified parameters and for the atomistic structures obtained from the original parameters at 0 ns and after 300 ns of relaxation.

S5. NP Self-Assembly

CG-MD simulations were employed to model the self-assembly of amphiphilic molecules into NPs within a water-filled cubic box at a concentration of 20 mM, a range previously validated as suitable for effective self-assembly in similar systems [16]. To ensure comparability across simulation box sizes, the six systems were designed with approximately consistent numbers of CG beads per NP (**Table S4**). Initially, amphiphiles were randomly dispersed, solvated with Martini water beads, and allowed to self-assemble spontaneously. Detailed simulation parameters are presented in **Table S1**.

Table S4. Structural composition and coarse-grained resolution of amphiphilic systems

Parameter	G1FF	G1HH	G1HF	G2FF	G2HH	G2HF
**Number of amphiphiles**	191	250	216	141	171	155
**Atoms per amphiphile**	106	106	106	150	150	150
**CG beads per molecule**	17	13	15	23	19	21

The equilibration protocol for the model initiated with energy minimization, followed by NVT equilibration at 310 K. Subsequent production runs were performed under NPT conditions at 310 K and 1 bar, continuing until a single stable NP formed in each system, achieved after approximately 1350 ns.

The solvent-accessible surface area (SASA), was used to exaine NP formation and equilibration. **Figure S4** shows the SASA profiles over the early 900ns course of the production simulations. The trajectories exhibited an initial decrease in surface area, indicative of compaction during assembly, followed by stabilization upon NP formation. Amphiphiles with hydrocarbon tails (HH) generally displayed higher stable SASA values compared to those with hybrid or fluorocarbon tails, suggesting looser packing for HH systems. Second-generation (G2) headgroups resulted in slightly larger SASA than first-generation (G1), attributable to the bulkier dendritic structures increasing surface exposure.

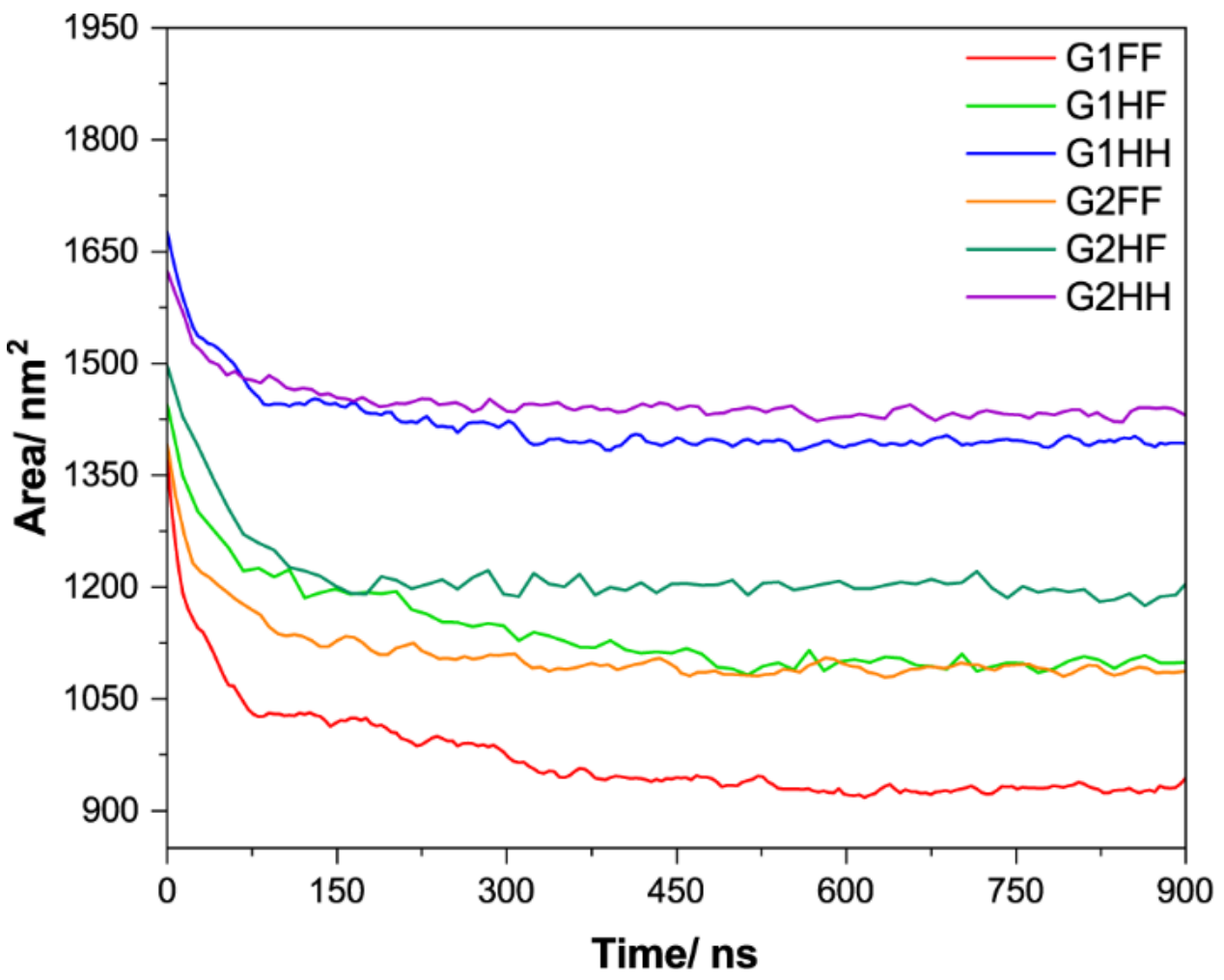

Figure S4. Solvent-accessible surface area (SASA) profiles for all NPs, calculated over production simulations.

S6. Lipid Membrane Compositions and Equilibration

Table S5 summerizes the structure of the membrane models used in this study. M-type membranes exhibit asymmetry, with outer leaflets enriched in sphingomyelin (SM) and 1,2-dioleoyl-sn-glycero-3-phosphocholine (DOPC), and inner leaflets enriched in 1,2-dioleoyl-sn-glycero-3-phosphoethanolamine (DOPE) and 1,2-dioleoyl-sn-glycero-3-phospho-L-serine (DOPS). M* membranes are more symmetric, with elevated DOPS in the outer leaflet. The numbers 0 and 33 in the model labels indicate the mol% of cholesterol in the membrane composition.

Table S5. Composition of the inner and outer leaflets in the symmetric and asymmetric cell membrane models.

Lipid Type	M33		M0		M*33		M*0	
	Outer leaflet	Inner leaflet	Outer leaflet	Inner leaflet	Outer leaflet	Inner leaflet	Outer leaflet	Inner leaflet
**Sphingomyelin (PSM)**	756	216	756	216	486	486	486	486
**DOPC**	828	252	828	252	540	540	540	540
**DOPE**	252	828	252	828	540	540	540	540
**DOPS**	0	540	0	540	270	270	270	270
**CHOL**	918	918	0	0	918	918	0	0
**Net charge**	0	-540	0	-540	-270	-270	-270	-270

****Notes: P: 16:0 palmitic acid. O: 18:1 Oleic acid. PC: Phosphatidyl Choline. PE: Phosphatidyl Ethanolamine. PS: Phosphatidyl Serine.**

All membrane models (M33, M0, M*33, M*0, and DOPC) were constructed using the CHARMM-GUI Martini Membrane Builder [21, 22]. Each membrane was solvated with Martini water beads on both sides, and Na^+ ions were added to achieve charge neutrality [23].

Membrane equilibration began with energy minimization, followed by NVT equilibration at 310 K. The membrane models were then further stabilized by NPT equilibration at 310 K, and 1 bar for 900 ns with detailed simulation parameters presented in **Table S1**

Properties such as membrane thickness, area per lipid (APL), and lipid tail order parameters (P_2), were assessed over the final 100 ns of the 900 ns production (**Figures S4-S6**) and summarized in **Table S6**. APL values ranged from 0.517 to 0.681 nm², showing consistency across inner and outer leaflets and between asymmetric and symmetric models. Membranes with 33% cholesterol exhibited reduced APL (~0.52 nm²) compared to cholesterol-free membranes (~0.66–0.68 nm²), reflecting enhanced lipid packing. Bilayer thickness ranged from 3.87 to 4.13 nm. Cholesterol-containing membranes were approximately 5–6% thicker (~4.12 nm) than cholesterol-free ones (~3.88–3.93 nm), consistent with height increases compensating for reduced surface area in incompressible bilayers. These trends are in agreement with previous experimental and simulation studies [24-27]. Notably, DOPS overexpression in symmetric membranes had minimal impact on APL and thickness, indicating that cholesterol is the primary regulator of structural properties.

Table S6. Structural properties (APL and thickness) of equilibrated membrane models averaged over the final 100 ns of simulations.

Membrane	Area Per Lipid (APL) (nm²)		Thickness (nm)
	Outer leaflet	Inner leaflet	
**M*0**	0.657 ± 0.007	0.657 ± 0.008	3.929 ± 0.033
**M*33**	0.519 ± 0.008	0.527 ± 0.008	4.126 ± 0.033
**DOPC**	0.681 ± 0.008	0.680 ± 0.009	3.872 ± 0.036
**M0**	0.659 ± 0.007	0.659 ± 0.007	3.924 ± 0.035
**M33**	0.517 ± 0.006	0.531 ± 0.007	4.120 ± 0.036

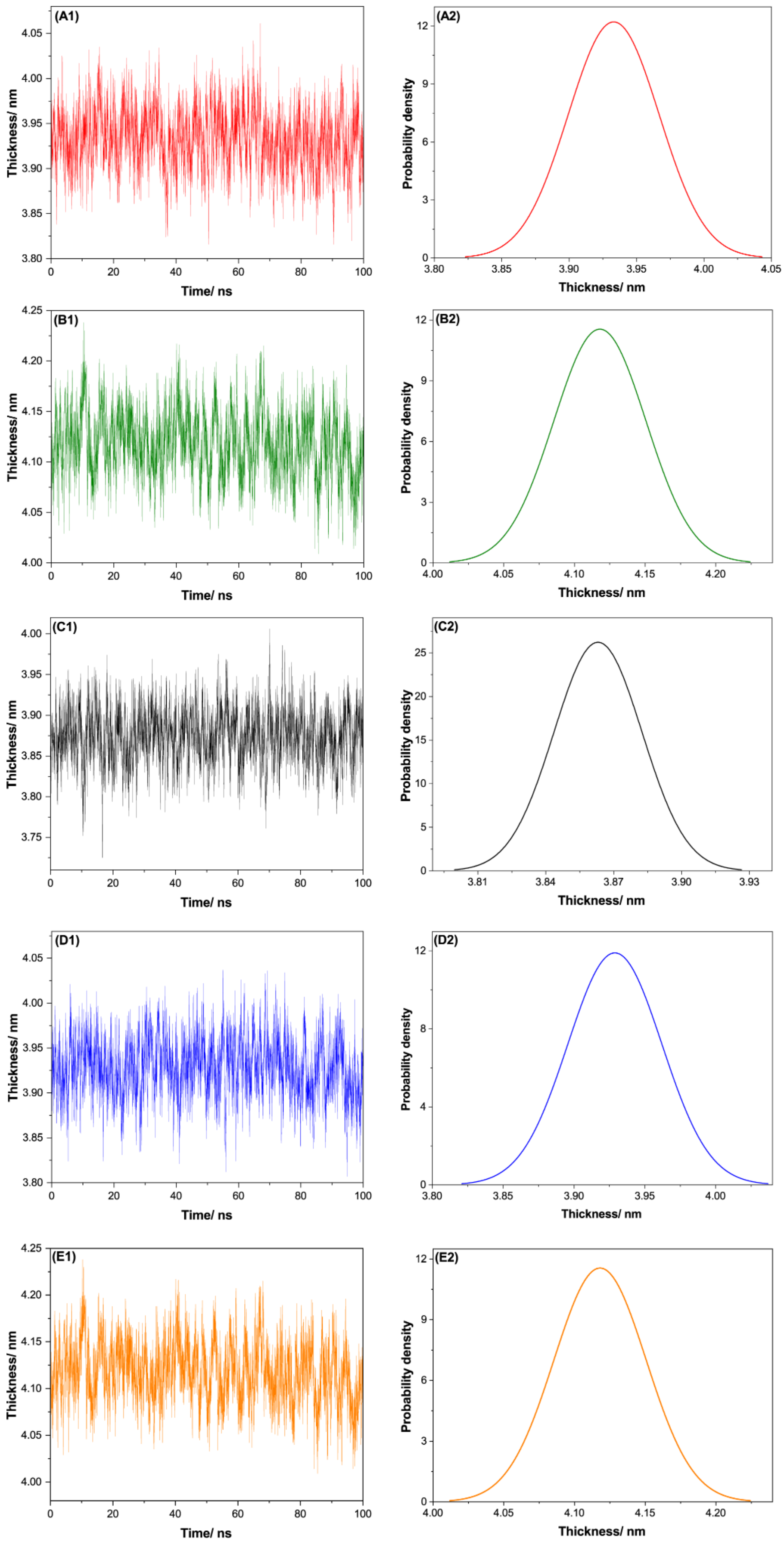

Figure S5. Time evolution (left) and probability density (right) of membrane thickness using the FATSLiM tool [28] for (A) M*0, (B) M*33, (C) DOPC ,(D) M0 and (E) M33.

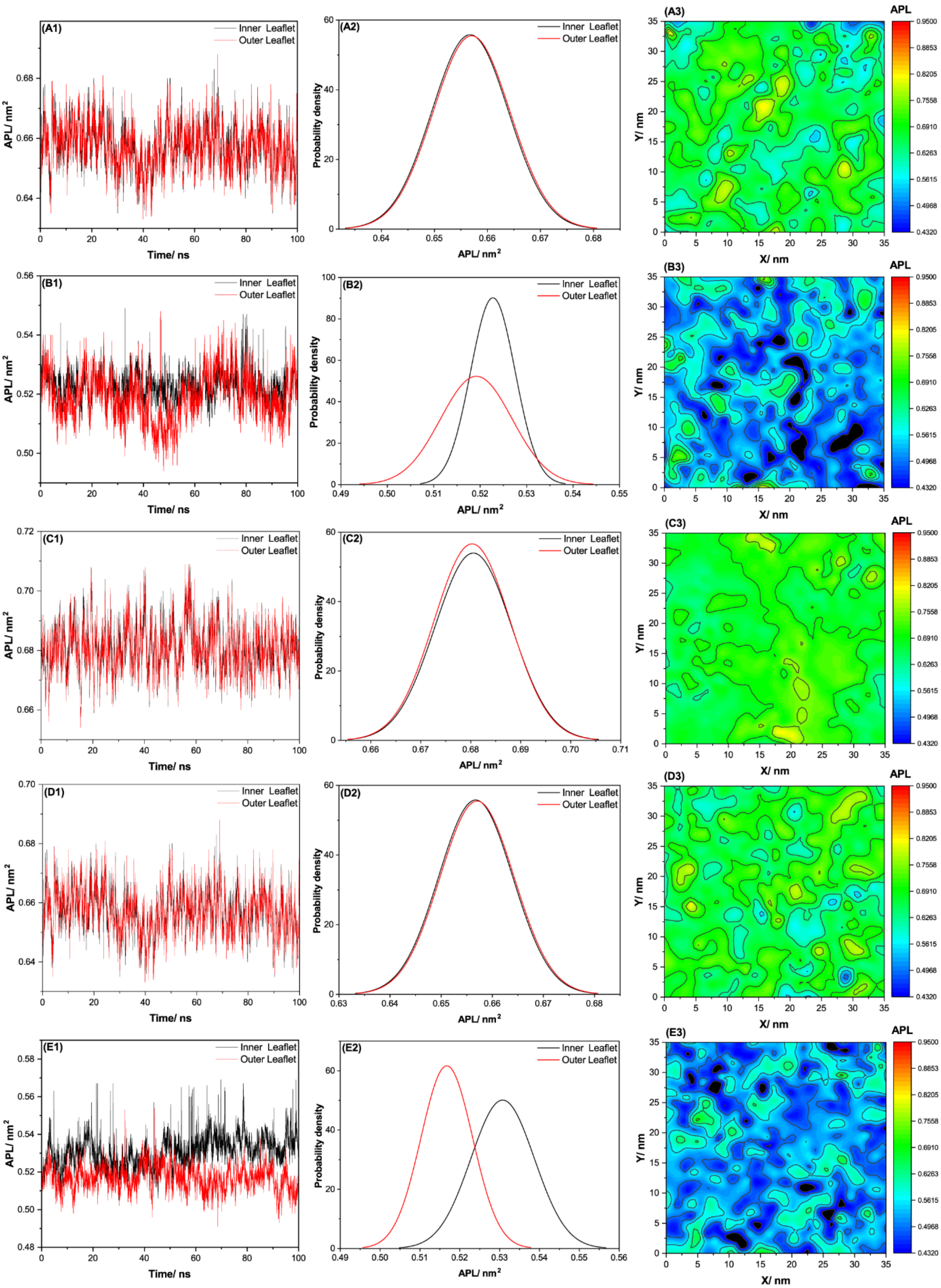

Figure S6. Time evolution (left), probability density (middle), and spatial mapping (right) of APL values, measured using FATSLiM tool for (A) M*0, (B) M*33, (C) DOPC ,(D) M0 and (E) M33.

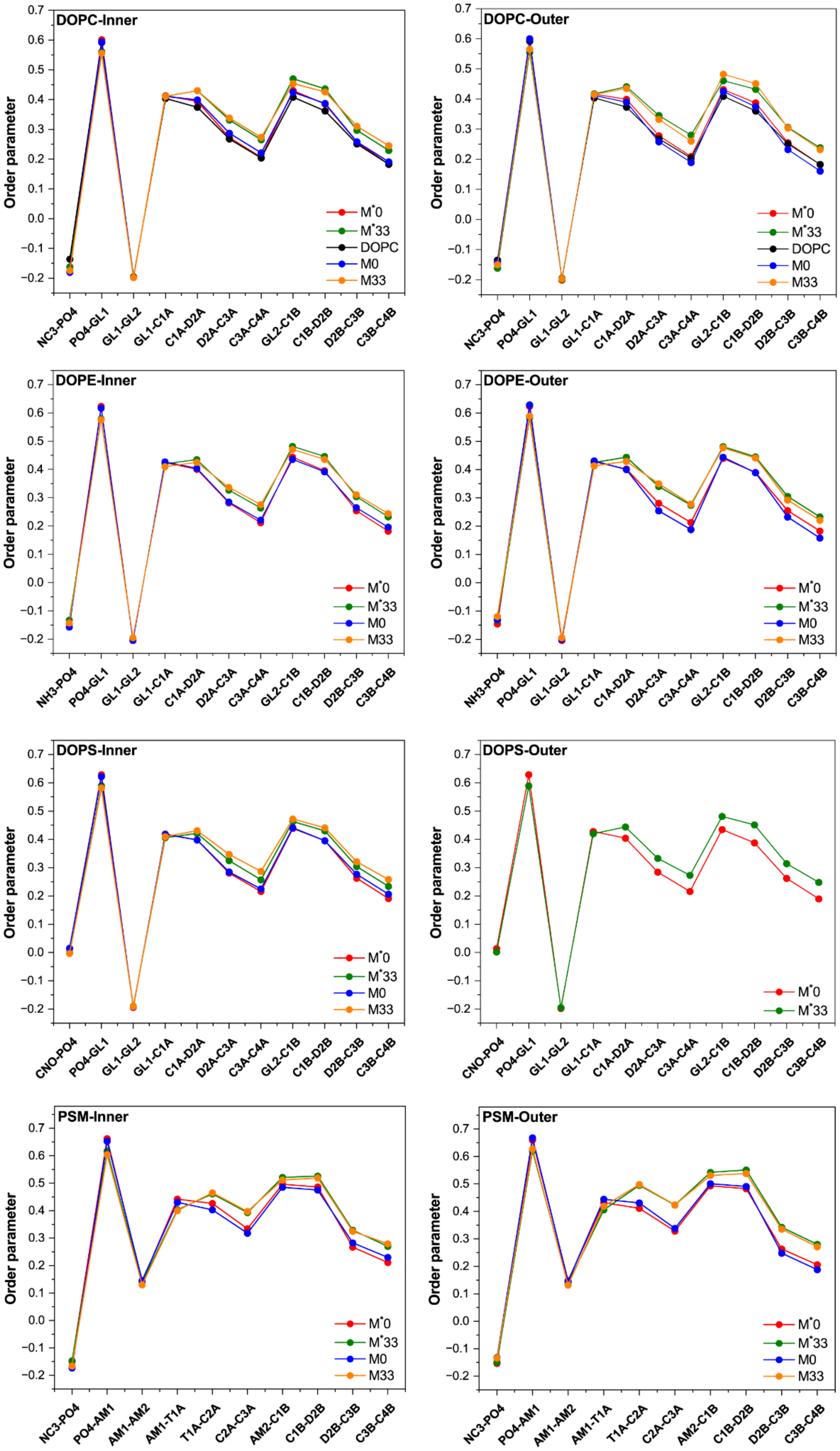

Figure S7. Lipid tail order parameters P_2 for phospholipid species in inner and outer leaflets, calculated using a custom script [29] $P_2 = (1/2)(3\cos^2\theta - 1)$ where θ is the angle between consecutive CG beads and the bilayer normal.

S7. self-assembled Structures and their validation

To further validate the CG self-assembled models, the equilibriated CG NP models were reverse mapped to atomistc models by using Backward method [30]. This included the projection of the CG structures onto atomistic coordinates, followed by two 500-step energy minimizations: one excluding nonbonded interactions in selected groups, and another including all interactions. NVT simulations at 310 K were then conducted with progressively increasing time steps (0.2, 0.5, 1.0, 2.0 fs) for relaxation. The reverse-mapped systems were then equilibrated for 100 ns under NPT conditions at 1 bar and 310 K, as detailed in **Table S1**. As shown in **Figure S8**, there is a good agreement between CG and atomistc self-assembled models .

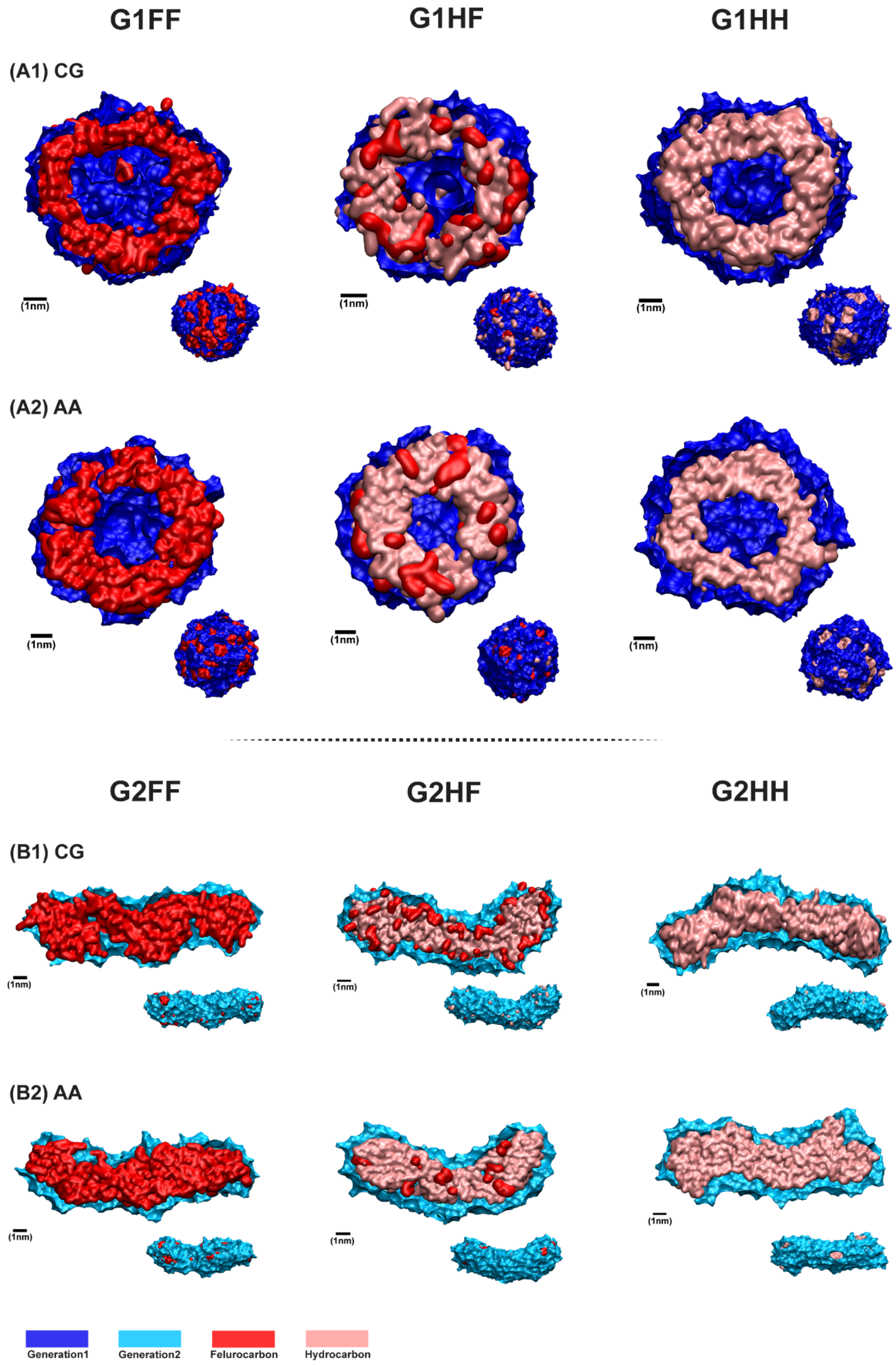

Figure S8. Self-assembled NP morphologies. (A1) CG representations of first-generation amphiphiles (G1HH, G1HF, G1FF); (A2) corresponding reverse-mapped atomistic structures. (B1) CG representations of second-generation amphiphiles (G2HH, G2HF, G2FF); (B2) corresponding reverse-mapped atomistic structures.

To confirm that the amphiphiles had sufficient mobility to form an equilibrated morphology, the root-mean-square deviation (RMSD) of the G1HF amphiphile molecules was calculated relative to the initial reverse-mapped structure throughout the 100 ns atomistic simulation. The RMSD profile showed a sharp increase during the first ~45 ns, followed by stable fluctuations that plateau around 4.9 nm, indicating that the system reached equilibrium (**Figure S9-A**). Additionally, the dynamic motion of individual amphiphile molecules was visualized using VMD snapshots extracted at regular intervals, demonstrating extensive reorientation and migration within the G1HF nanoparticle assembly (**Figure S9-B).**

To quantitatively assess the mobility of amphiphiles across all systems, self-diffusion coefficients (D) were calculated from the atomistic simulations (**Table S7**). Using the Einstein relation for three-dimensional diffusion, $\langle r^2 \rangle = 2dDt$ (where d = 3), the mean-squared displacement after 100 ns was determined for each system. For G1HF, with D = 0.043 $nm^2\ ns^{-1}$, the calculated displacement was r = 5.08 nm, which is significantly larger than twice the radius of gyration (2 × Rg = 1.30 nm). This substantial displacement, well exceeding the 2Rg threshold, confirms that the amphiphiles explored sufficient conformational space to achieve equilibrated morphologies. Similar mobility criteria were satisfied across all systems (**Table S7**), with root-mean-square displacements ranging from 3.63 to 6.57 nm, all substantially larger than their respective 2Rg values.

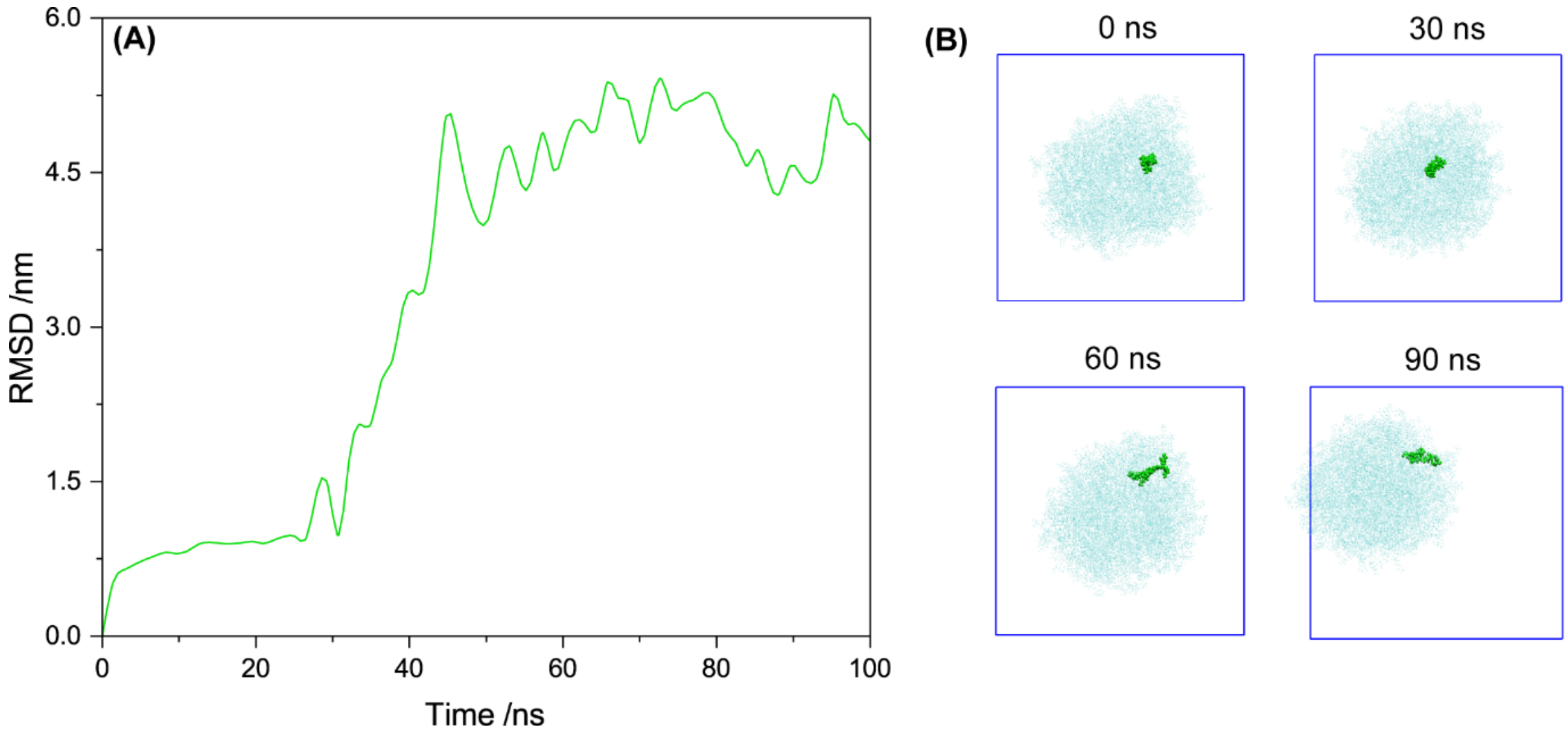

Figure S9. Mobility analysis of G1HF amphiphiles in the atomistic simulation. (A) RMSD of amphiphiles relative to the initial reverse-mapped structure over 100 ns, showing convergence to a plateau. (B) VMD snapshots showing the dynamic motion of a representative amphiphile molecule (highlighted in green) at 0 ns, 30 ns, 60 ns, and 90 ns.

Table S7. Self-diffusion coefficients (D), mean-squared displacements ($\langle r^2 \rangle = 2dDt$), corresponding root-mean-square displacements (r), and radius of gyration (Rg) for all amphiphilic systems.

System	D ($nm^2\ ns^{-1}$)	$r^2 = 2dDt$ (nm^2)	r (nm)	Rg (nm)
G1HH	0.02	13.41	3.66	0.62
G1HF	0.04	23.44	4.84	0.65
G1FF	0.06	37.06	6.09	0.65
G2HH	0.03	19.21	4.38	0.70
G2HF	0.07	43.03	6.56	0.72
G2FF	0.04	26.22	5.12	0.73

For fluorinated amphiphiles, we used modified Martini 3 parameters[14] to accurately represent fluorocarbon/hydrocarbon and fluorocarbon/water partitioning.

Bilayer thickness for G1 amphiphiles showed good agreement between atomistic and CG simulations (**Table S8**). Measurements were averaged over multiple cross-sections using VMD.

Table S8. Bilayer thickness comparison between atomistic and coarse-grained CG simulations.

NP	Bilayer Thickness (nm) (AA)	Bilayer Thickness (nm) (CG)
**G1FF**	2.1 ± 0.2	2.1 ± 0.2
**G1HH**	2.6 ± 0.2	2.4 ± 0.2
**G1HF**	2.3 ± 0.2	2.2 ± 0.2

Surface curvature (K_m) was analyzed using the Willard-Chandler method [31] (**Figure S10**) confirming that the CG model accurately replicated atomistic shapes, with the modified Martini parameters capturing structural features effectively.

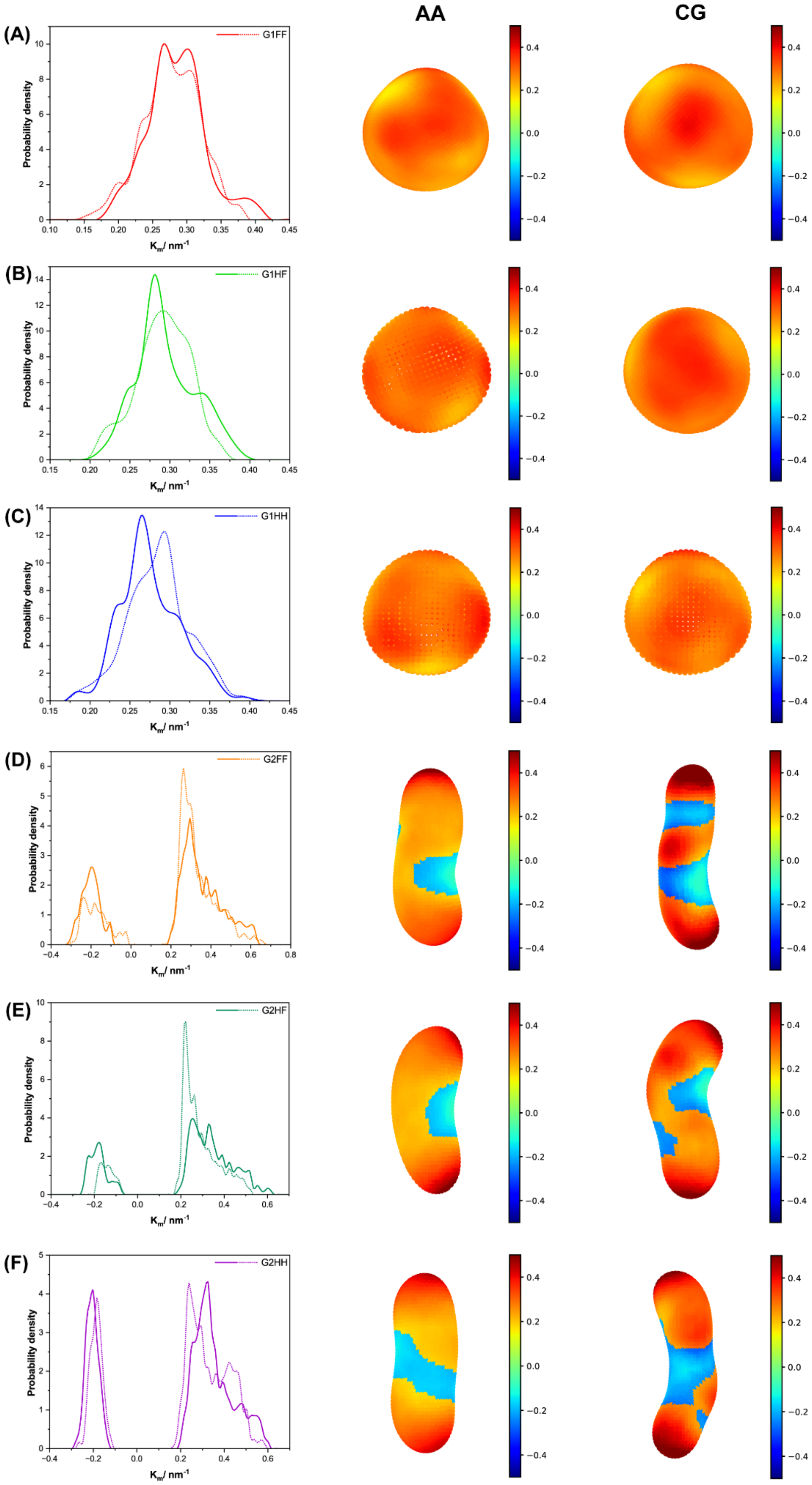

Figure S10. Mean curvature (K_m) analysis of self-assembled NPs, comparing atomistic (AA) and coarse-grained (CG) simulations for (A) G1FF, (B) G1HF, (C) G1HH, (D) G2FF, (E) G2HF, and (F) G2HH. The left panel shows the K_m histogram from atomistic (dashed line) and CG (solid line) simulations, the middle panel depicts the atomistic surface mapping, and the right panel presents the CG surface mapping.

To further validate the CG model against the atomistic simulations, radial distribution functions (RDFs) were employed to quantify local interactions between amphiphile head groups (-OH) and water. Additionally, the coordination number (CN), defined as the average number of water beads within a specified cutoff distance around an amphiphile bead, was used to assess hydration levels and NP-water interactions. RDF profiles for hydrophilic heads (-OH) and water reveal that atomistic simulations display a sharp peak (g(r) ≈ 1 at r ≈ 0.2 nm), indicative of strong, localized hydration shells. In contrast, CG simulations exhibit a broader peak (g(r) ≈ 1.7 at r ≈ 0.5 nm), which aligns with the reduced resolution inherent to coarse-graining but maintains qualitative trends in hydration behavior. The overall consistency in RDF shapes between CG and reverse-mapped atomistic models confirms the fidelity of the CG representation for capturing essential intermolecular interactions (**Figure S11**).

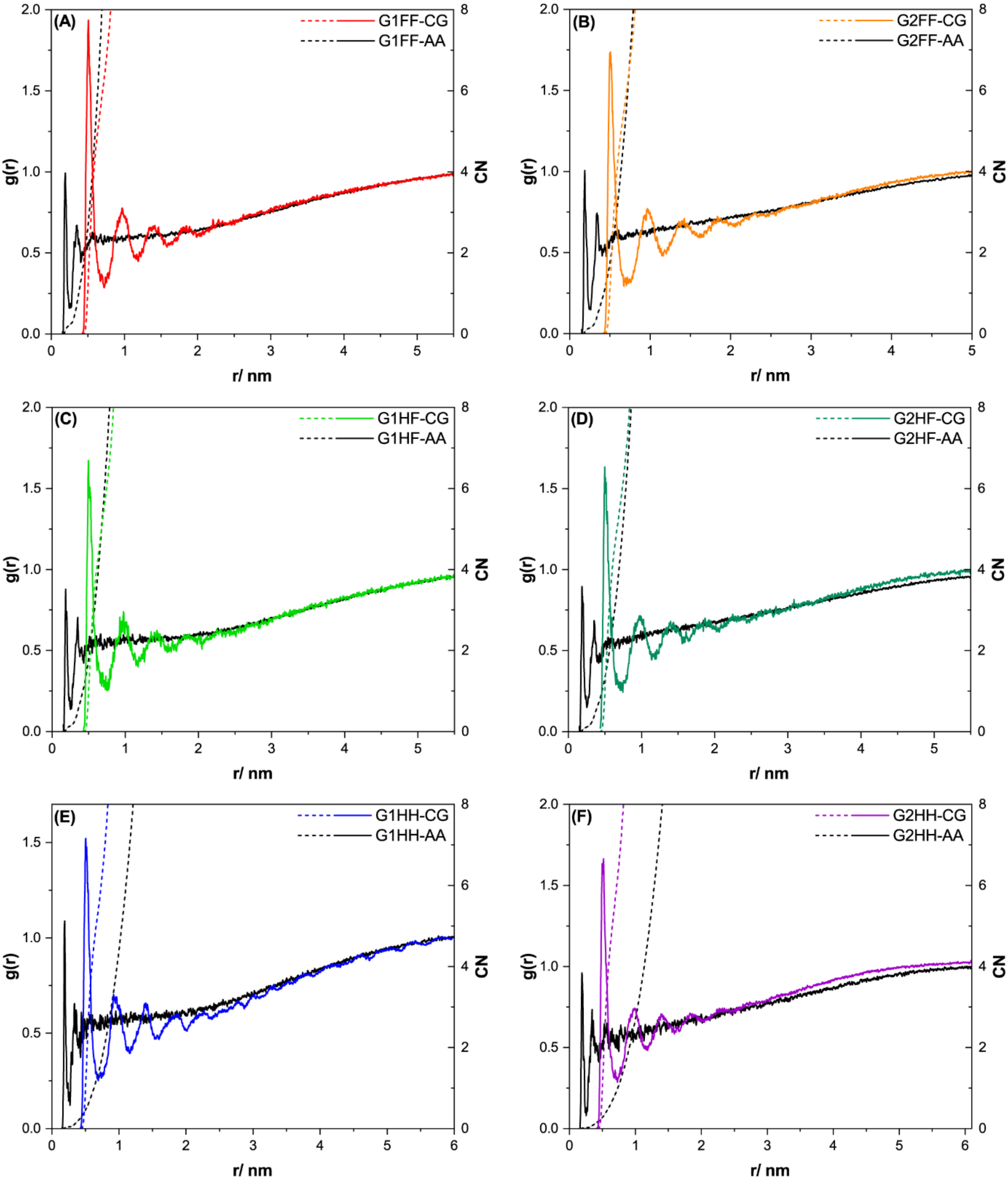

Figure S11. Radial distribution function (RDF) profiles for hydroxyl (-OH) groups and water molecules. The secondary axis indicates the coordination number. Comparisons are shown for (A) G1FF, (B) G2FF, (C) G1HH, (D) G2HH, (E) G1HF, (F) G2HF.

RDFs and CNs were analyzed to probe phase separation between hydrocarbon (H-Tail) and fluorocarbon (F-Tail) tails in the G1HF NP using a new CG parametrization. The H-Tail RDF, with H-Tail as reference, shows a sharp peak at r ≈ 0.34 nm (g(r) ≈ 68), and the F-Tail RDF, with H-Tail as reference, exhibits a peak at r ≈ 0.26 nm (g(r) ≈ 60) (**Figure S12**).

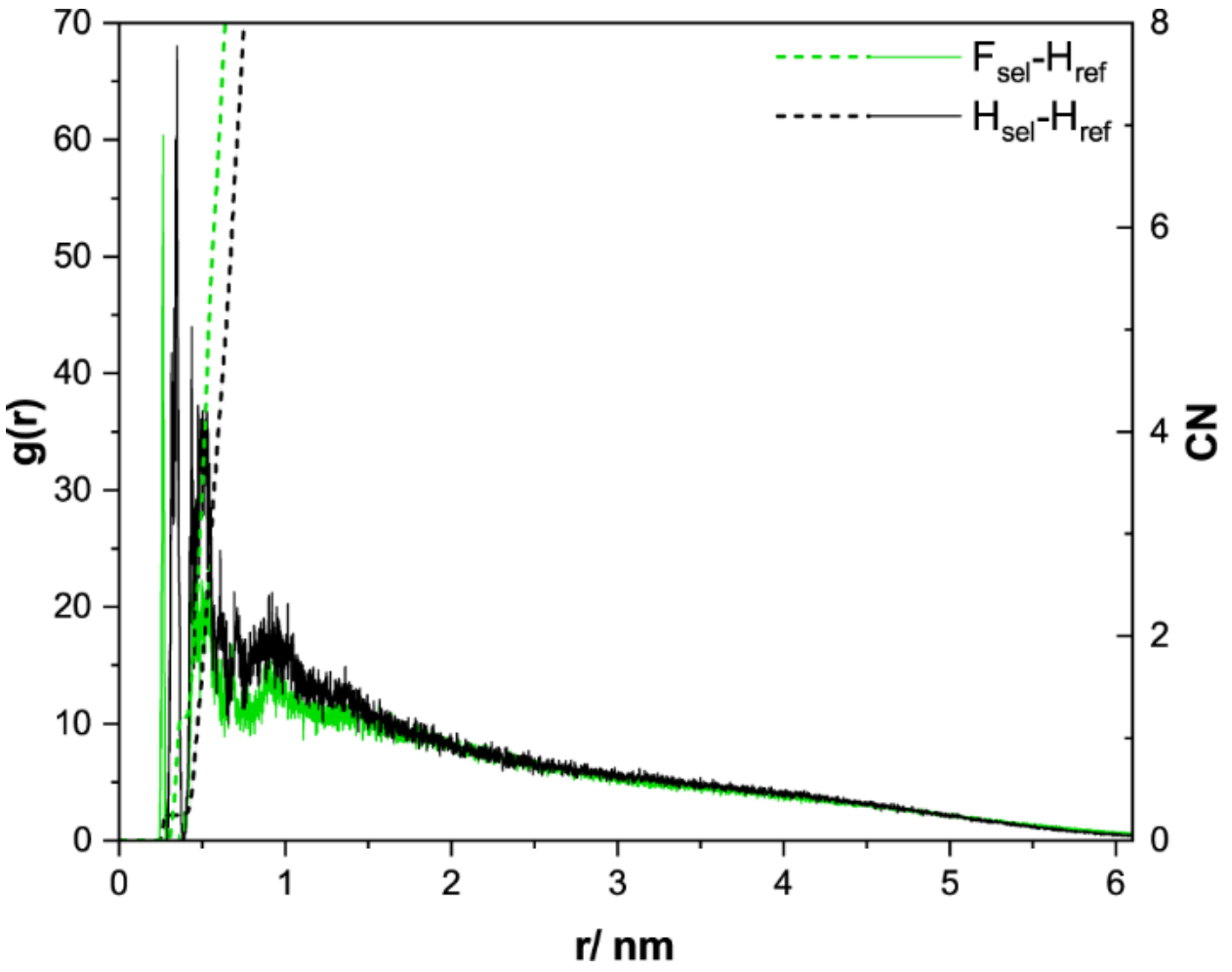

Figure S12. RDFs and CNs for tail interactions in the CG G1HF NP.

To investigate phase separation within the G1HF nanoparticle, we quantified pairwise bead–bead contacts between hydrocarbon (H) and fluorocarbon (F) tail segments over the final 200 ns of the NPT trajectory. Contacts were computed using a 0.6 nm cutoff. Average contact counts were: H–H= 217, F–F= 217, H–F= 216. The near-identical H–F contact number compared to same-type contacts indicates complete mixing of H and F tails, with no evidence of phase separation in G1HF.

S8. Steered Molecular Dynamics Simulations and Convergence Analysis

Steered molecular dynamics (SMD) simulations, incorporating center-of-mass pulling and umbrella sampling, were employed to compute the potential of mean force (PMF) for amphiphile dissociation from NPs in aqueous solution [32]. The PMF was derived by averaging profiles from four independent amphiphiles per NP, pulled at a rate of 0.001 nm/ps with a force constant of 1000 kJ/mol/nm², and reconstructed using the weighted histogram analysis method (WHAM) [33]. The dissociation free energy (ΔG_{nano}) quantifies NP stability, with higher values indicating stronger cohesion.

Given that membrane asymmetry has a negligible impact on transfer kinetics relative to cholesterol content, thermodynamic analyses were focused on M*0 and M*33 membranes to isolate the effects of cholesterol. Two amphiphiles from each NP were positioned 1 nm above the membrane surface and equilibrated through energy minimization, NVT, and NPT stages to achieve stable integration (equilibration similar to the **S5** section). Subsequently, two amphiphiles were extracted from each membrane using SMD, following the protocol described above to achieve the membrane extraction free energy ($\Delta G_{membrane}$), with higher values indicating stronger affinity.

To ensure the reliability of PMF calculations for dissociation, umbrella sampling was conducted with varying durations to assess convergence. For each system, the PMF was obtained from pulling one amphiphile from the center of mass (COM), with free energy analyzed over the last 5 ns of different run lengths. **Figure S13** illustrates this for G2FF: (A) PMF profiles at various times as a function of COM distance, showing convergence at 60 ns; (B) histograms of umbrella windows to confirm sampling adequacy. This approach was applied to all systems to determine appropriate simulation durations.

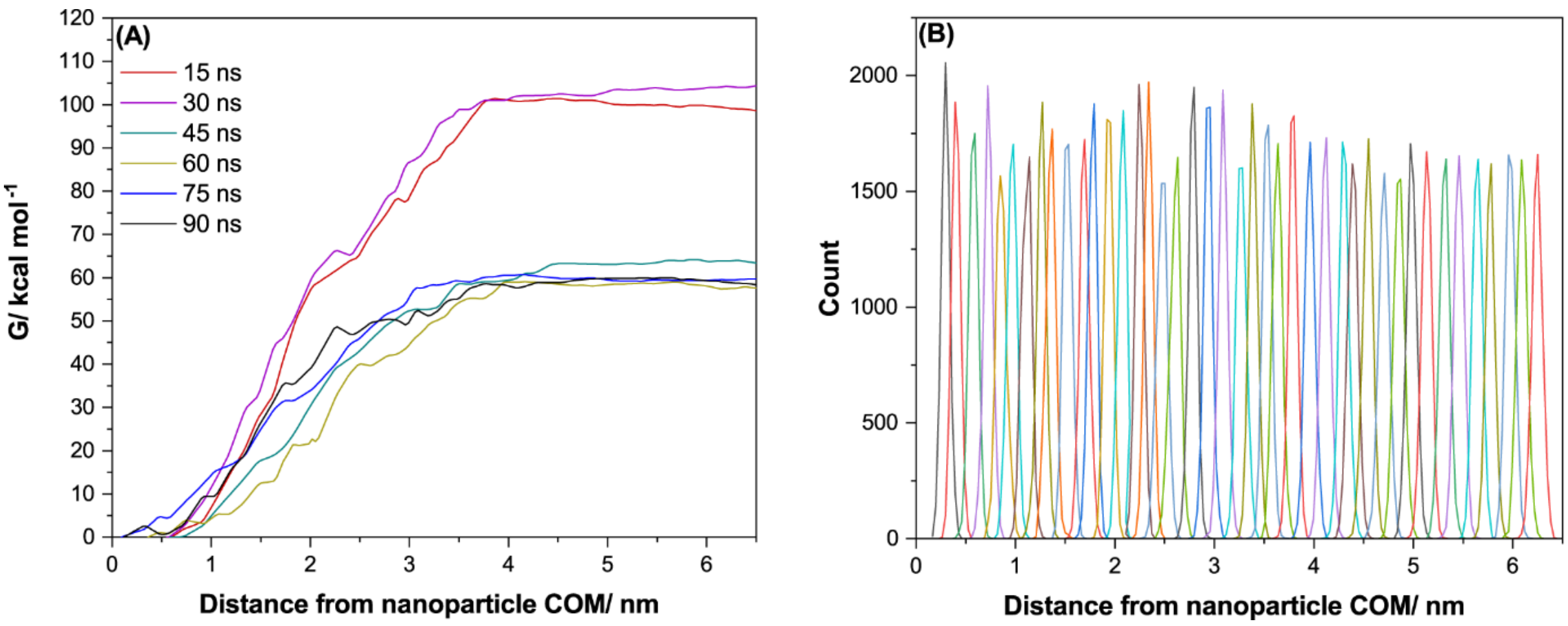

Figure S13. Convergence analysis of PMF computations for the G2FF NP. (A) Free energy profiles for a single amphiphile pulled from the NP's center of mass (COM) at various simulation times. (B) Histogram of umbrella sampling windows.

S9. NP-Membrane Interactions

MD simulations of NP–membrane interactions were conducted for each pair of NP-membrane (30 simulations in total). Initially, the NP was positioned 6 nm above the membrane surface, with positional restraints applied during equilibration. Following equilibration (as detailed in **S5**), the restraints were released, and the NP was pulled to approximately 1 nm from the membrane using a harmonic potential, establishing the starting configuration for interaction studies. **Figure S14** illustrates the initial positioning of NPs at ~1 nm from the membrane surface for these interaction studies.

Afrer EM and NVT stages, production simulations were performed under NPT at 310 K, 1 bar, for 1.5 µs to provide sufficient time for interactions to develop (as detailed in **Table S1**)

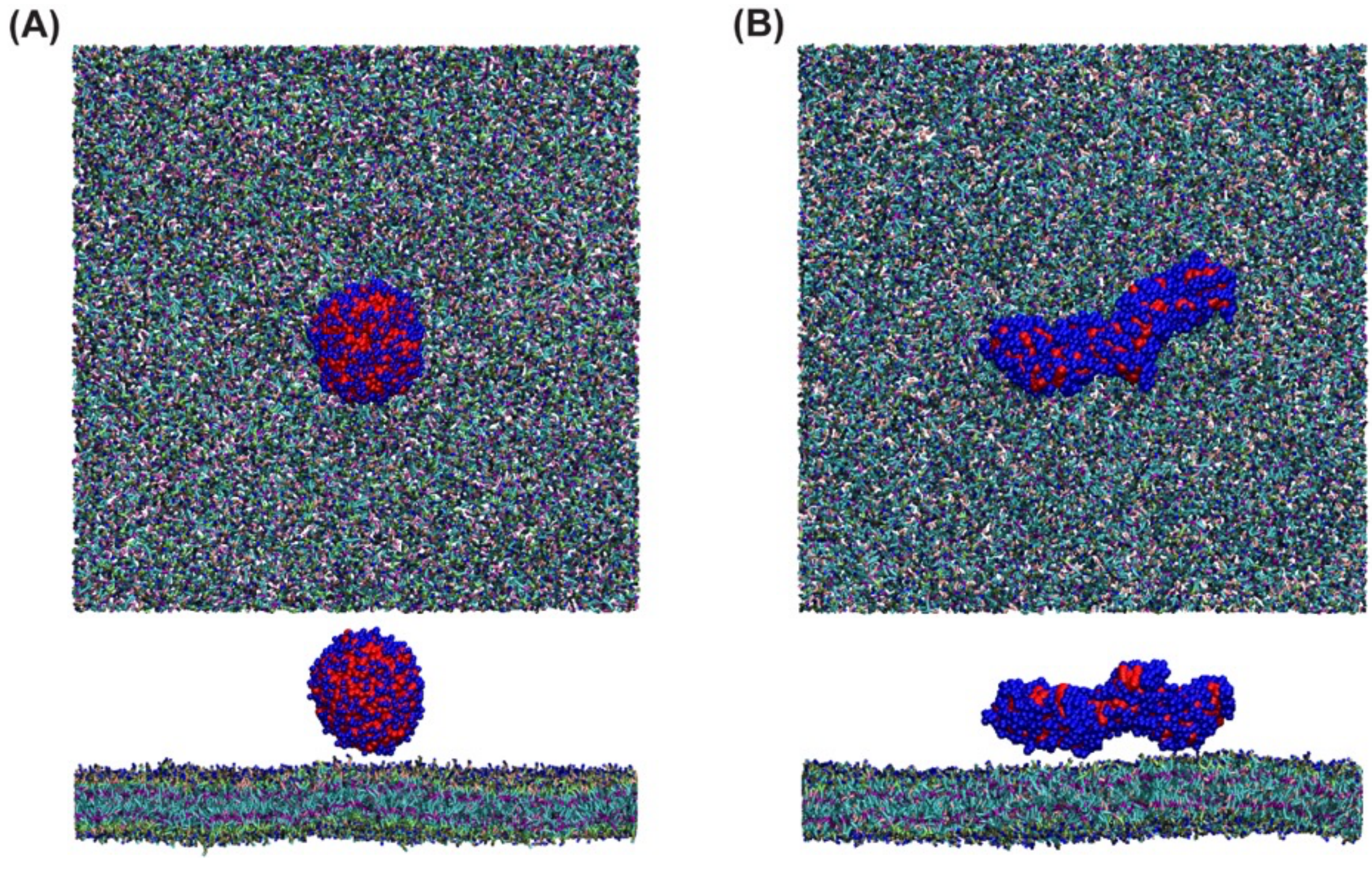

Figure S14. Initial positioning of NPs at ~1 nm from the membrane surface for interaction studies. Panel (A) shows first-generation NPs (top and side views), and panel (B) shows second-generation NPs (top and side views). For enhanced visual clarity, the hydrophilic heads of all NPs are rendered in blue, while the hydrophobic tails are shown in red.

Part B: Experimental

S10. General synthetic and analytical methods

All commercially available compounds were purchased from Sigma-Aldrich, TCI and abcr Chemicals. Indocarbocyanine dye was purchased from Mivenion GmbH (Berlin, Germany). Compound pG1-NH_2 was synthesized according to previous procedures. [34]

To monitor the progress of the reaction, pre-coated thin-layer chromatography (TLC) plate (Merck silica gel 60F254) was used with visualization of the spots on TLC using potassium permanganate solution. For column chromatography, a CombiFlash Rf+ Teledyne Isco system was used with RediSep Silver Normal Phase Silica columns. Millipore water was used for preparation of samples for their physio-chemical characterization and transport studies. UV-Vis and fluorescence measurements were performed using a Tecan Spark® plate reader using either Sarstedt transparent 96 flat or Brand black 96 flat well plates. NMR spectra were measured with the spectrometers ECX 400 (400 MHz) and ECP500 (500 MHz) from JEOL and Avance 500 (500 MHz) and Avance 700 (700 MHz) from Bruker. Mass spectra were measured on a 6210 ESI- TOF and 6230 ESI-TOF from Agilent. Abbreviations used in NMR signal reporting are the following: m, multiplet; s, singlet, d, doublet; t, triplet; q, quartet; quint, quintet; sext, sextet; sept, septet. The Images were analysed using the Fiji version of ImageJ 1.54p. A scale bar was added to all images [35, 36].

S11. Synthetic procedure and characterisation data

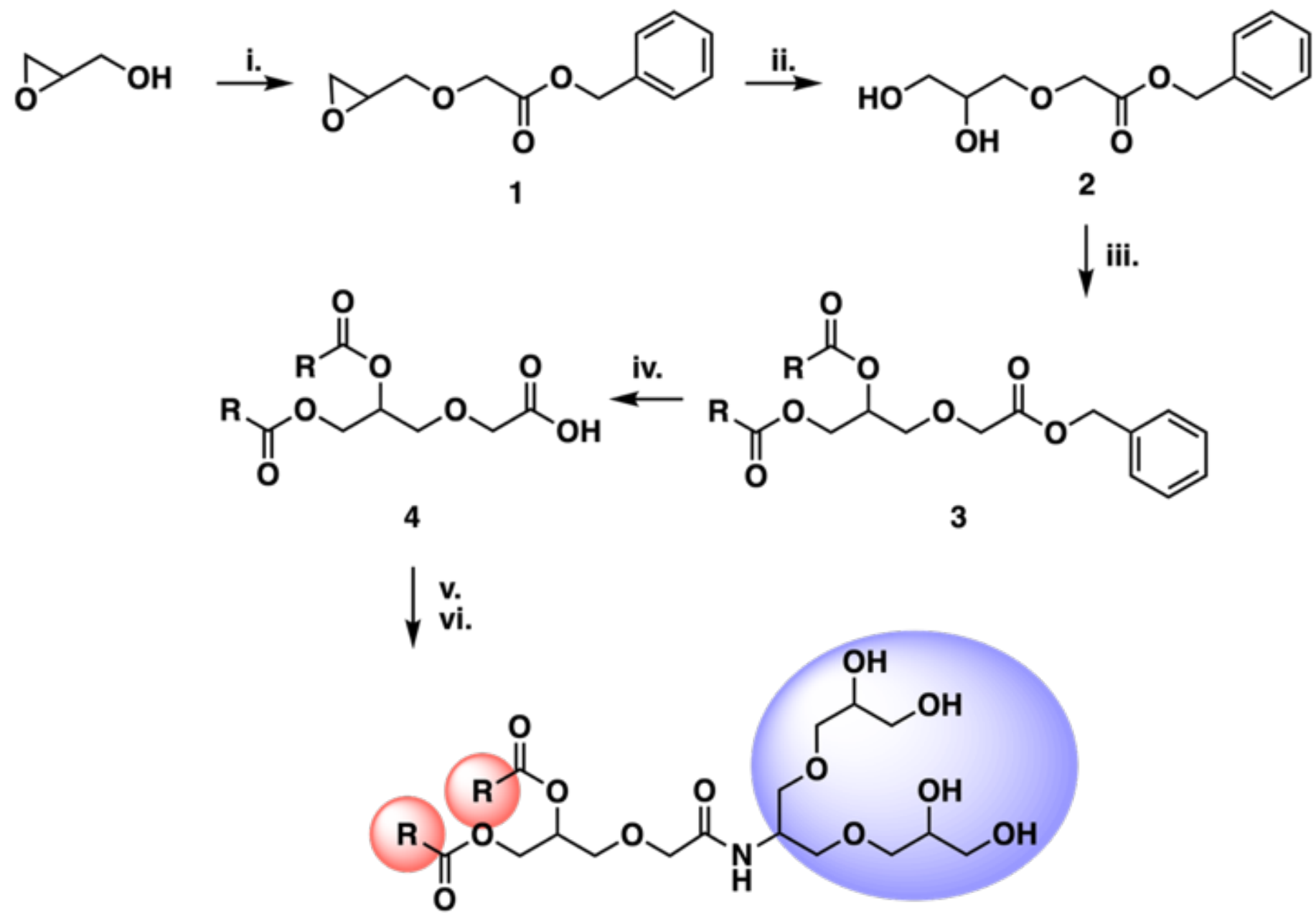

Compound name	R group
G1-HH	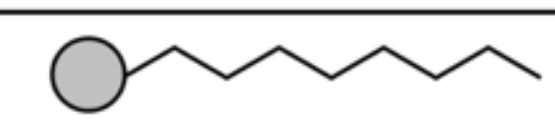
G1-FF	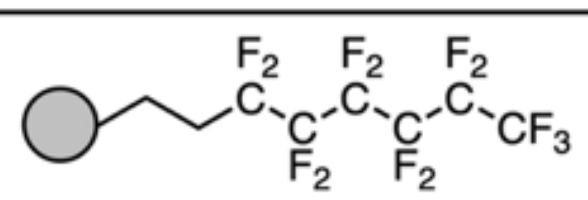

Figure S15: Synthetic scheme of amphiphiles G1HH and G1FF.

- ***Compound 1***

In a pre-dried Schlenk flask, a 60% dispersion of NaH in mineral oil (2.70 g, 67.5 mmol, 1.0 eq.) was dissolved in anhydrous THF (30 ml) under argon at 0 °C. To this solution was added dropwise glycidol (5.00 g, 67.5 mmol, 1.0 eq.), releasing hydrogen gas. Subsequently, benzyl bromoacetate was added dropwise (15.46 g, 67.5 mmol, 1.0 eq.) and the reaction was left to stir overnight, progressively coming back to room temperature, and its progress was monitored by thin-layer chromatography (20% EtOAc/CyH). The reaction was neutralised using water and extracted using diethyl ether (3 × 20 ml). The combined organic phases were washed with brine (1 × 30 ml), dried over Na_2SO_4 and the solvent was removed by rotary evaporation. The crude product was purified by flash chromatography (10→15% EtOAc/CyH + 1% Et_3N) to obtain a colourless oil, **compound 1** (7.80 g, 35.1 mmol, **52%**); TLC: 20% EtOAc/CyH, R_f = 0.23; ^{1}H NMR (400 MHz, $(CD_3)_2CO$): δ 7.42–7.34 (m, 5H), 5.18 (s, 2H), 4.22 (s, 2H), 3.85 (dd, *J* = 11.6, 2.9 Hz, 1H), 3.43 (dd, *J* = 11.6, 6.4 Hz, 1H), 3.11 (dddd, *J* = 6.4, 4.2, 2.9, 2.7 Hz, 1H), 2.71 (dd, *J* = 5.1, 4.2 Hz, 1H), 2.53 (dd, *J* = 5.1, 2.7 Hz, 1H) ppm; ^{13}C NMR (101 MHz, $(CD_3)_2CO$): δ 170.7, 137.1, 129.3, 129.0, 73.1, 68.8, 66.6, 50.9, 44.0 ppm; HRMS (ESI-TOF) m/z: $[M + Na]^+$ calcd. for $C_{12}H_{14}O_4$ 245.2298, found 245.0845.

- ***Compound 2***

This procedure was adapted from the literature. [37] In a flask were added **compound 1** (6.28 g, 28.3 mmol) and deionized water (225 ml), which were then heated at 100 °C for 2 h 45 min in a pre-heated oil bath. Water was removed using rotary evaporation and the product was dissolved in DCM to be dried using Na_2SO_4. The solvent was again removed using rotary evaporation, yielding a colourless oil, **compound 2** (6.01 g, 25.0 mmol, **89%**); ^{1}H NMR (400 MHz, CD_3OD): δ 7.39–7.31 (m, 5H), 5.20 (s, 2H), 4.20 (s, 2H), 3.80–3.75 (m, 1H), 3.65–3.51 (m, 4H) ppm; ^{13}C NMR (151 MHz, CD_3OD): δ 172.4, 137.2, 129.6, 129.4, 74.1, 72.1, 69.4, 67.6, 64.3 ppm; HRMS (ESI-TOF) m/z: $[M + Na]^+$ calcd. for $C_{12}H_{16}O_5$ 263.0895, found 263.0952.

- ***Compound 3-HH***

In a pre-dried Schlenk flask was dissolved nonanoic acid (1.65 g, 10.4 mmol, 2.5 eq.) in anhydrous DCM (40 ml) at 0 °C under argon. To the resulting solution was added DMAP (1.02 g, 8.35 mmol, 2.0 eq.) and EDC·HCl (2.39 g, 12.5 mmol, 3.0 eq.), then the reaction mixture was stirred 30 min at 0 °C. Following this, **compound 2** (1.00 g, 4.16 mmol, 1.0 eq.) was added and stirred at 30 °C overnight. The reaction was extracted using DCM (3 × 20 ml). The combined organic phases were washed with brine (1 × 20 ml), dried over Na_2SO_4 and the solvent was removed by rotary evaporation. The crude product was purified by flash chromatography (10% EtOAc/CyH) to yield a colourless oil, **compound 3-HH** (1.88 g, 3.61 mmol, **87%**). TLC: 30% EtOAc/CyH, R_f = 0.76; ^{1}H NMR (500 MHz, CD_2Cl_2): δ 7.39–7.31 (m, 5H), 5.21–5.17 (m, 3H), 4.39–4.24 (m, 1H), 4.17–4.06 (m, 3H), 3.72–3.63 (m, 2H), 2.35–2.27 (m, 4H), 1.61–1.55 (m, 4H), 1.33–1.22 (m, 20H), 0.88 (t, *J* = 7.0 Hz, 6H) ppm; HRMS (ESI-TOF) m/z: $[M + Na]^+$ calcd. for $C_{30}H_{48}O_7$ 543.329774, found 543.3253.

- ***Compound 3-FF***

In a pre-dried Schlenk flask was dissolved 4,4,5,5,6,6,7,7,8,8,9,9,9-tridecafluorononanoic acid (6.12 g, 15.6 mmol, 2.5 eq.) in anhydrous DCM (30 ml) and DMF (30 ml) under argon at 0 °C. Following this, DMAP (1.53 g, 12.5 mmol, 2.0 eq.) and EDC·HCl (1.53 g, 18.7 mmol, 3.0 eq.) were added and stirred for 30 min, then **compound 2** (1.50 g, 6.24 mmol, 1.0 eq.) was added using DCM (10 ml) and the resulting reaction mixture was stirred at 30 °C overnight. After removing the solvent by rotary

evaporation, the reaction was extracted using EtOAc (3 × 20 ml). The combined organic phases were washed with brine (1 × 30 ml), dried over Na_2SO_4 and the solvent was removed by rotary evaporation. The crude product was purified by flash chromatography (20→30% EtOAc/CyH) to obtain a colourless oil, **compound 3-FF** (4.20 g, 4.25 mmol, **68%**); TLC: 30% EtOAc/CyH, R_f = 0.64; ^1H NMR (400 MHz, $CDCl_3$): δ 7.40–7.29 (m, 5H), 5.30–5.25 (m, 1H), 5.18 (s, 2H), 4.48 (dd, J = 12.3, 3.4 Hz, 1H), 4.27 (dd, J = 12.3, 6.5 Hz, 1H), 4.14 (s, 2H), 3.78–3.70 (m, 2H), 2.68–2.61 (m, 4H), 2.53–2.40 (m, 4H) ppm; ^{13}C NMR (101 MHz, $CDCl_3$): δ 170.9, 170.7, 169.9, 135.4, 128.8, 128.7, 128.6, 71.0, 69.7, 68.5, 66.9, 63.4, 26.6, 25.4 ppm; ^{19}F NMR (376 MHz, $CDCl_3$): δ -81.00 (t), -114.89 (hex), -122.00 (br s), -122.98 (br s), -123.61 (br s), -126.30 (br s) ppm; HRMS (ESI-TOF) m/z: $[M + Na]^+$ calcd. for $C_{30}H_{22}F_{26}O_7$ 1011.0848, found 1011.0869.

- ***Compound 4-HH***

In a Schlenk flask under argon was added Pd/C (200 mg, 10 wt%) and a solution of **compound 3-HH** (2.10 g, 4.04 mmol, 100 wt%) in THF/H_2O (9 : 1, 100 ml). The flask was further evacuated and put under argon. A hydrogen balloon was connected and the reaction was stirred at room temperature for 3 days, refilling the hydrogen balloon as needed. The catalyst was removed by paper filtration and washed with THF and H_2O, then the solvents were removed by rotary evaporation to yield a colourless oil, **compound 4-HH** (1.71 g, 3.96 mmol, **98%**). ^1H NMR (400 MHz, CD_3OD): δ 5.21 (br s, 1H), 4.42–4.39 (m, 1H), 4.17–4.09 (m, 3H), 3.71 (br s, 2H), 2.32–2.22 (m, 4H), 1.60 (br s, 4H), 1.31 (s, 20H), 0.91 (br s, 6H) ppm.

- ***Compound 4-FF***

In a Schlenk flask was dissolved **compound 3-FF** (3.82 g, 3.86 mmol) in THF and H_2O (9 : 1, 100 ml) under argon, then Pd/C catalyst (382 mg, 10 wt%) was added. The reaction flask was evacuated and put under H_2 gas using a balloon, which was refilled as needed over the course of the reaction, and the mixture was stirred at room temperature for 3 days. The reaction mixture was then filtered on Celite and washed with THF, then solvents were removed under reduced pressure to yield a white hard solid, **compound 4-FF** (3.37 g, 3.77 mmol, **98%**). ^1H NMR (400 MHz, CD_3OD): δ 5.31–5.26 (m, 1H), 4.49 (dd, J = 12.1, 3.1 Hz, 1H), 4.28 (dd, J = 12.1, 6.8 Hz, 1H), 4.10 (s, 2H), 3.75 (d, J = 5.2 Hz, 2H), 2.74–2.65 (m, 4H), 2.63–2.49 (m, 4H) ppm; ^{19}F NMR (376 MHz, THF-D_8): δ -81.71 (br s), -115.21 (br s), -122.43 (br s), -123.38 (br s), -124.08 (br s), -126.79 (br s) ppm; HRMS (ESI-TOF) m/z: $[M – H]^–$ calcd. for $C_{23}H_{16}F_{26}O_7$ 897.0403, found 897.0469.

- ***pG1HH***

In a pre-dried Schlenk flask was dissolved **compound 4-HH** (1.71 g, 3.96 mmol, 1.0 eq.) in anhydrous DMF (50 ml). Following this, EDC·HCl (1.14 g, 5.94 mmol, 1.5 eq.) and HOBt (642 mg, 4.75 mmol, 1.2 eq.) were added and the resulting mixture was stirred at 35 °C overnight. Subsequently, Et_3N (801 mg, 7.92 mmol, 2.0 eq.) was added and stirred for 30 min, then pG1-NH_2 (1.26 g, 3.96 mmol, 1.0 eq.) was added using anhydrous DMF (15 ml) and stirred for 48 h at 35 °C. Solvent was removed by rotary evaporation and the product was purified by flash chromatography to yield a yellow oil, **pG1HH** (1.29 g, 1.76 mmol, **45%**). ^1H NMR (400 MHz, CD_3OD): δ 4.42–4.34 (m, 1H), 4.29–4.00 (m, 8H), 3.75–3.45 (m, 10H), 2.37– 2.30 m, 4H), 1.62–1.60 (m, 4H), 1.39–1.27 (m, 32H), 0.91 (t, J = 6.8 Hz, 6H) ppm; HRMS (ESI-TOF) m/z: $[M + Na]^+$ calcd. for $C_{38}H_{69}NO_{12}$ 754.4720, found 754.4727.

- ***pG1FF***

In a pre-dried Schlenk flask was dissolved **compound 4-FF** (1.00 g, 1.11 mmol, 1.0 eq.), pG1-NH_2 (390 mg, 1.22 mmol, 1.1 eq.), EDC·HCl (260 mg, 1.34 mmol, 1.2 eq.), HOBt (150 mg, 1.11 mmol, 1.0 eq.) in anhydrous DMF (100 ml). The resulting reaction mixture was stirred at 50 °C overnight, the solvent was removed by rotary evaporation, the reaction was extracted using DCM. The combined organic phases were washed with brine (1 × 30 ml), dried over Na_2SO_4 and the solvent was removed by rotary evaporation. The crude product was purified by flash chromatography (70% EtOAc/CyH + 1% Et_3N) to obtain a light red oil (1.25 g). This product was further purified by RP-HPLC (90% MeOH/H_2O) to yield a colourless cloudy oil, which crystallised overnight, **pG1FF** (1.03 g, 0.857 mmol, **77%**). ^{1}H NMR (600 MHz, CD_3OD): δ 5.34 (br s, 1H), 4.50 (dt, J = 12.0, 2.3 Hz, 1H), 4.30–4.21 (m, 4H), 4.06–3.99 (m, 4H), 3.77–3.71 (m, 4H), 3.64–3.49 (m, 8H), 2.76–2.66 (m, 4H), 2.60–2.49 (m, 4H), 1.38 (s, 6H), 1.32 (s, 6H) ppm; ^{13}C NMR (151 MHz, CD_3OD) δ 210.0, 172.3, 172.2, 171.7, 110.6, 76.1, 73.2, 73.1, 72.3, 72.3, 71.5, 71.1, 71.0, 70.8, 69.3, 67.5, 67.5, 67.5, 67.0, 64.4, 54.8, 49.8, 49.8, 49.7, 30.8, 30.7, 27.4, 27.3, 27.1, 27.1, 26.4, 26.3, 26.3, 26.1, 26.1, 26.1, 25.6 ppm; ^{19}F NMR (565 MHz, CD_3OD): δ -82.40 (t), -115.58– -115.84 (m), -122.85 (br s), -123.83 (br s), -124.49 (br s), -127.30 (br s) ppm; HRMS (ESI-TOF) m/z: [M + Na]$^+$ calcd. for $C_{38}H_{43}F_{26}NO_{12}$ 1222.2268, found 1222.2312.

- ***G1HH***

In a flask was dissolved **pG1HH** (1.19 g, 1.63 mmol) in MeOH (10 ml) and Dowex® 50WX8 (H) (1.19 g, 100 wt%) was added, then the resulting reaction mixture was stirred at 35 °C for 24 h. The product was filtered and washed with MeOH, the solvent was removed by rotary evaporation and purification was done by RP-HPLC (60→80% MeOH/H_2O) to yield a colorless viscous oil, **G1HH** (140 mg, 0.21 mmol, **13%**). ^{1}H NMR (500 MHz, CD_3OD): δ 5.30–5.26 (m, 1H), 4.40 (dd, *J* = 12.3, 3.4 Hz, 1H), 4.25 (pentet, *J* = 5.4 Hz, 1H), 4.18 (dd, *J* = 12.3, 6.5 Hz, 1H), 4.05–3.98 (m, 2H), 3.78–3.74 (m, 2H), 3.70 (d, *J* = 5.4 Hz, 2H), 3.63–3.46 (m, 12H), 2.37–2.31 (m, 4H), 1.65–1.58 (m, 4H), 1.36–1.27 (m, 20H), 0.91 (t, *J* = 7.0 Hz, 6H) ppm; ^{13}C NMR (151 MHz, CD_3OD): δ 175.0, 174.7, 171.9, 73.7, 73.6, 72.2, 72.1, 71.5, 71.5, 71.0, 64.5, 64.4, 63.7, 49.9, 49.9, 35.1, 34.9, 33.0, 30.4, 30.4, 30.3, 30.3, 30.2, 30.2, 26.1, 26.0, 23.7, 14.5 ppm; HRMS (ESI-TOF) m/z: [M + Na]$^+$ calcd. for $C_{32}H_{61}NO_{12}$ 674.4094, found 674.4085.

- ***G1FF***

In a flask was dissolved **pG1FF** (631 mg, 0.526 mmol) in MeOH (160 ml) and Dowex® 50WX8 (H) (1.956 g, 310 wt%) was added, then the resulting reaction mixture was stirred at 50 °C for 24 h. The product was filtered and washed with MeOH, and the solvent was removed by rotary evaporation to yield a white gummy solid, **G1FF** (561 mg, 0.501 mmol, **95%**). ^{1}H NMR (700 MHz, CD_3OD): δ 5.35–5.32 (m, 1H), 4.50 (dd, *J* = 12.3, 3.1 Hz, 1H), 4.29–4.23 (m, 2H), 4.05–4.00 (m, 2H), 3.77–3.72 (m, 4H), 3.64–3.46 (12H), 2.75–2.66 (m, 4H), 2.60–2.50 (m, 4H) ppm; ^{13}C NMR (176 MHz, CD_3OD): δ 172.4, 73.8, 73.6, 72.3, 72.2, 72.2, 71.5, 71.1, 71.1, 70.8, 64.4, 27.4, 27.3, 27.2, 26.4, 26.3, 26.1 ppm; ^{19}F NMR (376 MHz, CD_3OD): δ -82.36 (t), -115.70– -115.93 (m), -122.87 (br s), -123.86 (br s), -124.54 (br s), -127.30 (br s) ppm; HRMS (ESI-TOF) m/z: [M + Na]$^+$ calcd. for $C_{32}H_{35}F_{26}NO_{12}$ 1142.1642, found 1142.1694.

- ***G1-HF***

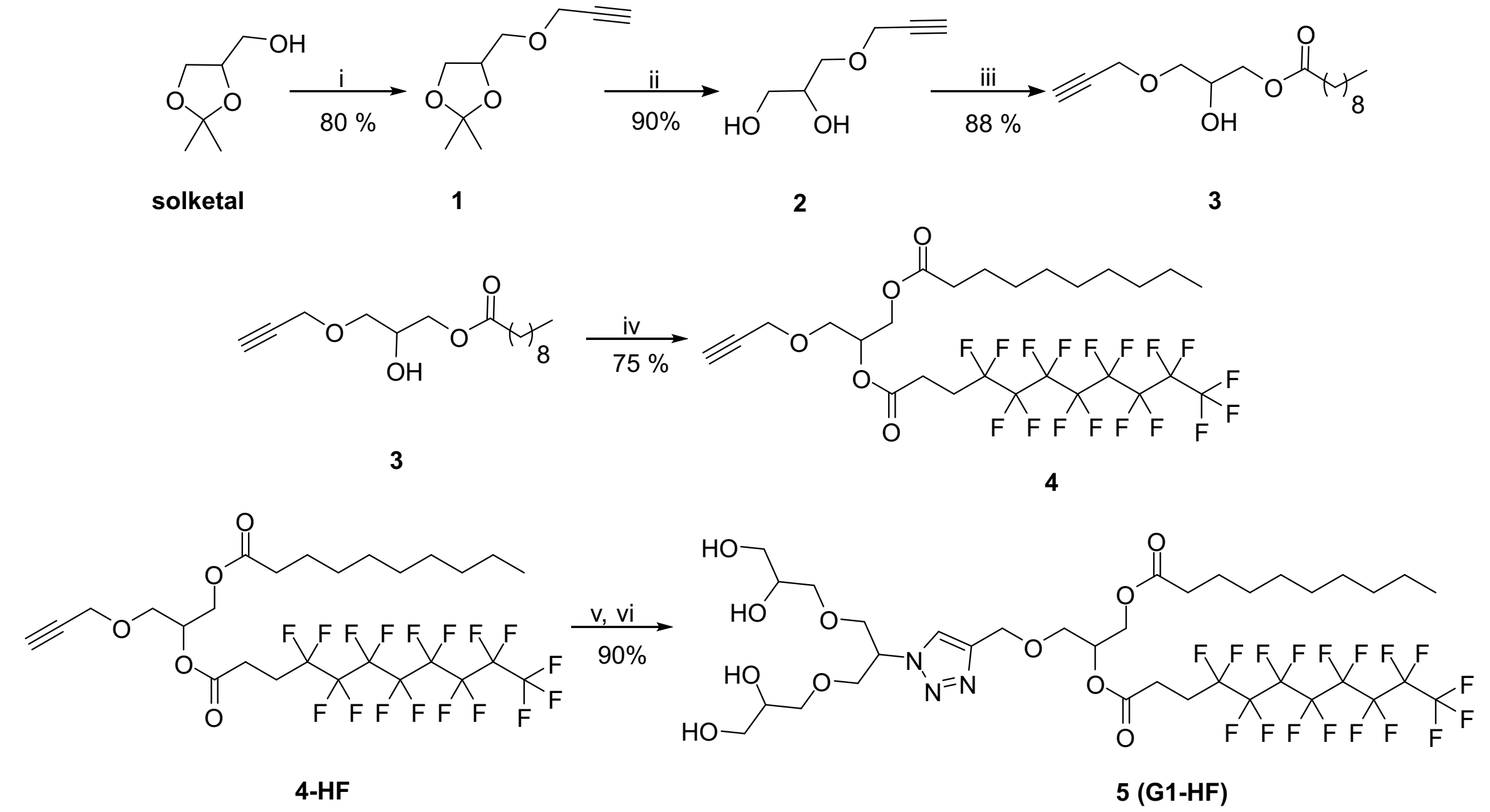

Figure S16. a) Synthesis of G1-HF amphiphile: i) NaH, propargyl bromide, THF, rt, 12 h. ii) DOWEX-50, MeOH, Water, 50 ℃, 12 h. iii) Vinyl decanoate, Novozym-435, THF, 35 ℃, 6 h. iv) 4,4,5,5,6,6,7,7,8,8,9,9,10,10,11,11,11-heptadecafluoroundecanoic acid, EDC.HCl, DMAP, DCM, rt, 24 h. v) protected G1azide, copper (I) catalyst, DMF, 60 ℃, 24 h. vi) DOWEX-50, MeOH, 50 ℃, 12 h.

Synthetic procedure and characterisation data

The alkyne-functionalized precursor 4-HF was synthesized according to the procedure previously reported by Rashmi *et al*.

In a pre-dried Schlenk flask was dissolved **compound 4-HF** (0.50 g, 0.67 mmol, 1.0 eq.), protected G1-N_3 (232 mg, 0.73 mmol, 1.1 eq.), Copper (I) catalyst (10 mg) in anhydrous DMF (50 ml). The resulting reaction mixture was stirred at 60 °C overnight, the solvent was removed by rotary evaporation, the reaction was extracted using DCM. The combined organic phases were dried over Na_2SO_4 and the solvent was removed by rotary evaporation. The crude product was purified by flash chromatography (25% EtOAc/CyH) to obtain a light yellow oil (0.65 g).

This obtained protected product (0.5 g) was then dissolved in methanol (50 ml) and treated with DOWEX (200 mg) in the presence of a few drops of water. The reaction mixture was stirred at 50 ℃ overnight. After removal of the resin by filtration, the solvent was evaporated under reduced pressure to afford **G1-HF** as an off-white solid in 90 % yield.

^{1}H NMR (600 MHz, CD_3OD): δ 0.89 (t, J = 6 Hz, 3H), 1.34-1.29 (m, 12H), 1.59 (p, J = 6 Hz, 2H), 2.30 (t, J = 6 Hz, 2H), 2.59–2.52 (m, 2H), 2.70–2.66 (m, 2H), 3.56–3.44 (m, 5H), 3.64–3.61 (m, 1H), 3.72-3.68 (m, 3H), 4.01-3.91 (m, 5H), 4.20-4.14 (m, 2H), 4.40-4.37 (m,1H), 4.66-4.61 (m, 2H), 4.85 (br s, 4H) 5.02-4.99 (m, 1H), 5.25-5.23 (m, 1H), 8.12-8.09 (m, 1H) ppm; ^{13}C NMR (151 MHz, CD_3OD) δ 174.7, 171.7, 144.8, 125.0, 110.2, 75.7, 75.7, 73.5, 73.4, 72.9, 72.2, 71.8, 71.7, 71.0, 70.9, 69.1, 69.1, 67.0, 67.0, 64.8, 63.9, 63.9, 63.3, 62.1, 34.5, 32.7, 30.3, 30.1, 30.1, 29.9, 26.7, 26.0, 25.6, 25.3, 25.2, 23.4, 14.1 ppm; ^{19}F NMR (565 MHz, CD_3OD): δ ppm; HRMS (ESI-TOF) m/z: $[M + Na]^+$ calcd. for $C_{36}H_{50}F_{17}N_3O_{11}$ 1023.7268, found 1023.6412.

S12. Characterisation of amphiphile self-assembly in water

- ***Preparation of amphiphile solutions***

Amphiphiles are dissolved in pure water to reach a 7 mM concentration. To dissolve them, they are vortexed for 30 seconds, sonicated for 10 minutes then again vortexed for 30 seconds. The solutions are left to equilibrate overnight then characterised.

- ***DLS***

Method

DLS measurements were carried out using the Malvern Zetasizer Ultra (Malvern Instruments Limited, U.K.) equipped with a 10 mW He–Ne laser operating at a wavelength of 632.8 nm. The scattered light was detected using the backscattering setting at an angle of 173° (NIBS, noninvasive backscatter). All measurements were done in triplicates and carried out in 10 mm square quartz cuvettes (Hellma Analytics).

Measurement data

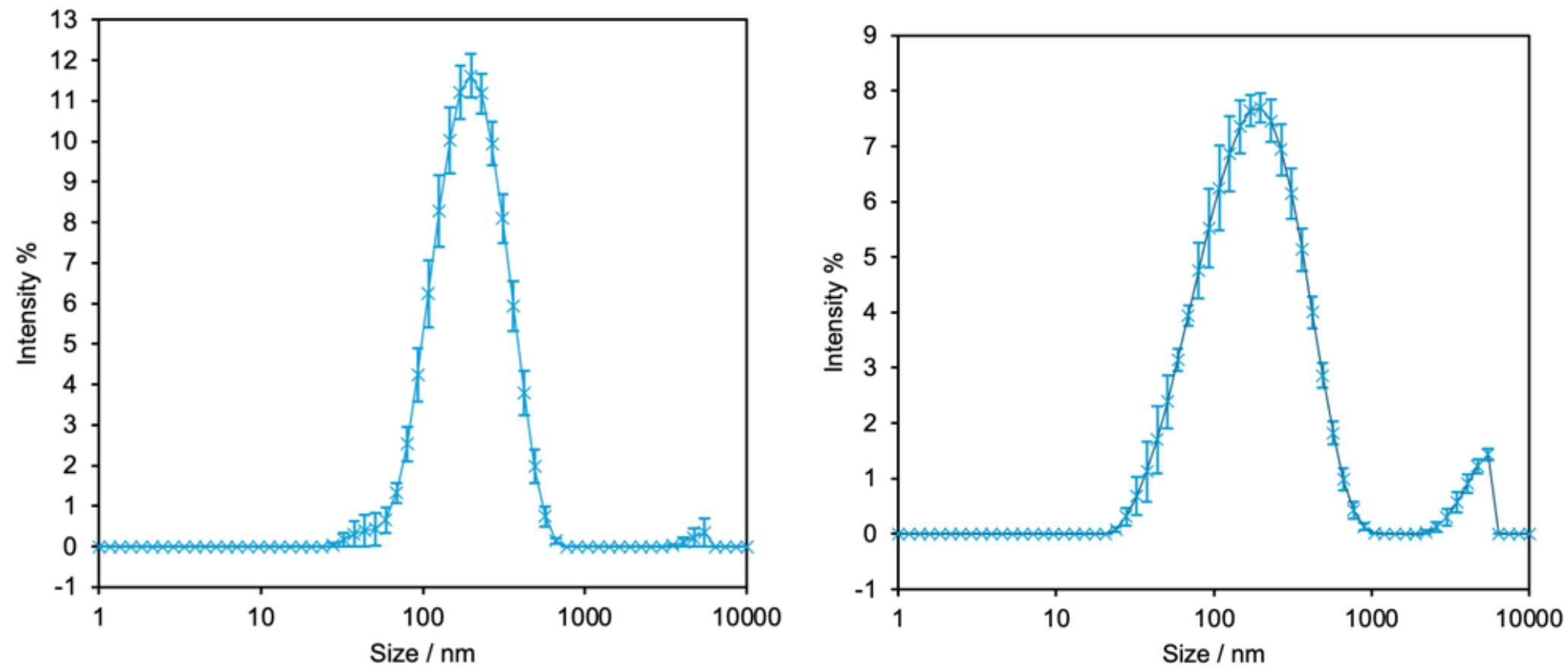

Figure S17: DLS plots of G1HH (left) and G1FF (right) with standard errors, dissolved in water with concentration of 7 mM.

Table S9: Summary of DLS results of G1HH and G1FF 7 mM solutions in water.

Amphiphile	G1HH	G1FF
**Z-average / nm**	166 ± 2	144 ± 3
**PDI**	0.26 ± 0.02	0.40±0.02

- ***Cryo-TEM***

Method

The samples for cryogenic transmission electron microscopy (cryo–TEM) were vetrified using a vitrobot Mark IV (ThermoFisher Scientific Inc., Waltham, MA, USA). The chamber's climate was set to 20 °C and 100% humidity. After placing a droplet (4 µl) of the sample solution on a hydrophilized (60 s Plasma treatment at 8 W using a BALTEC MED 020 device) 200 mesh copper grid covered with a perforated holey carbon film support (Quantifoil Micro Tools GmbH, Großlöbichau, Germany) excess fluid was automatically blotted off to create an ultrathin layer (typical thickness of 100 nm) of the solution spanning the holes of the supported film. The grids were immediately propelled into liquid ethane at its freezing point (–184°C). This ultra-fast cooling of the aqueous solution (vitrification) is necessary to avoid crystallization and thus produce ultra-thin, artifact-free cryo-samples. The vitrified grids were assembled to so-called autogrids, which can be stored under liquid nitrogen before use.

Autrogrids were then transferred into a Talos Arctica transmission electron microscope (ThermoFisher Scientific Inc., Waltham, MA, USA) using the microscope's autoloader transfer routine. The microscope is illuminated by an X-FEG cathode operated at 200 kV acceleration voltage. Micrographs were recorded on a Falcon 3CE direct electron detector at full size (4K) at a magnification of 28.000× using the microscopes' low-dose protocol. The defocus was chosen to be 4 µm in all cases to create sufficient phase contrast.

- ***Additional micrographs***

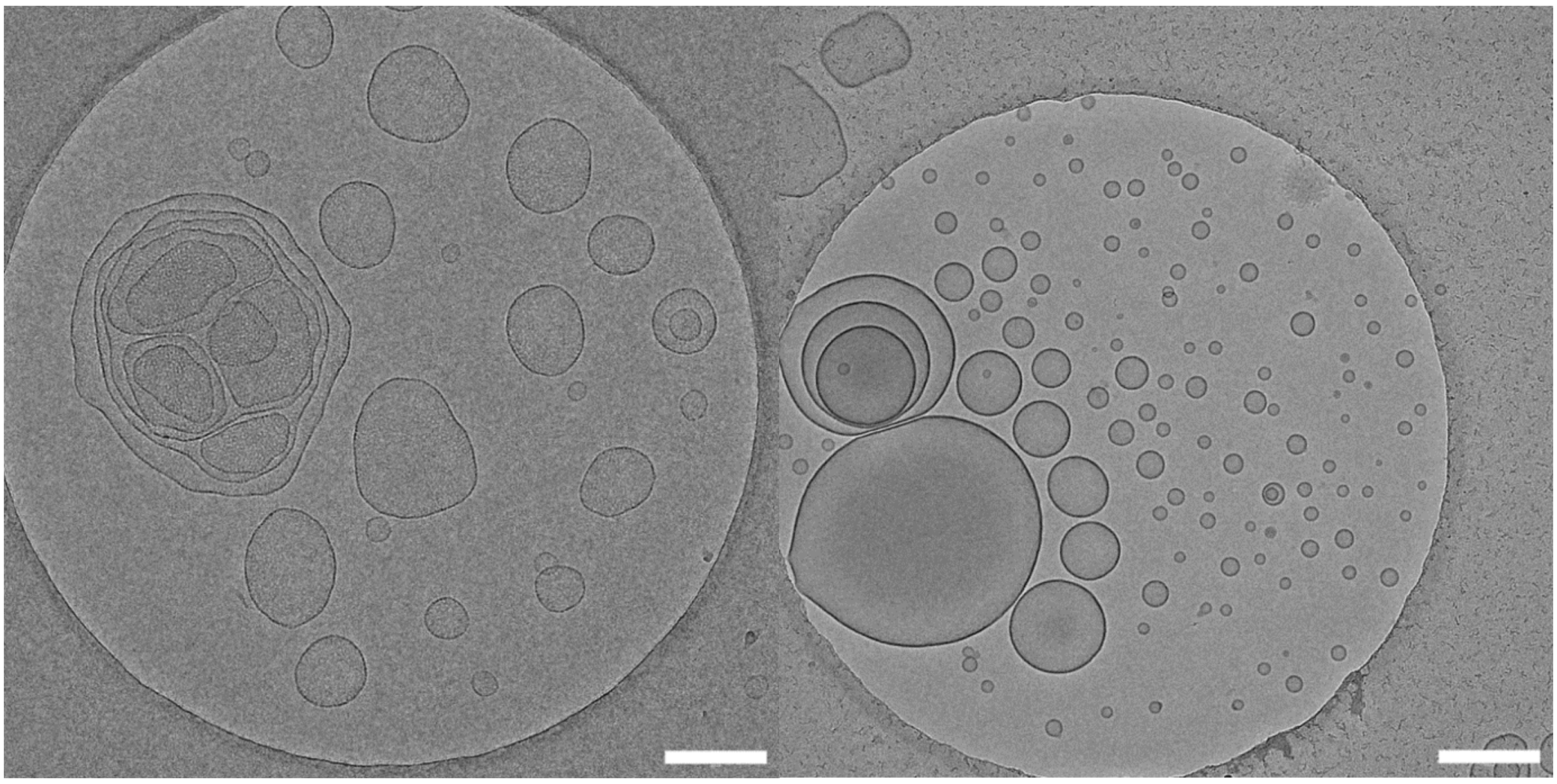

Figure S18: Comparison of the aggregation behaviour of G1HH (left) and G1FF (right) in water at concentrations of 7 mM using cryo-TEM; scale bar is 200 nm.

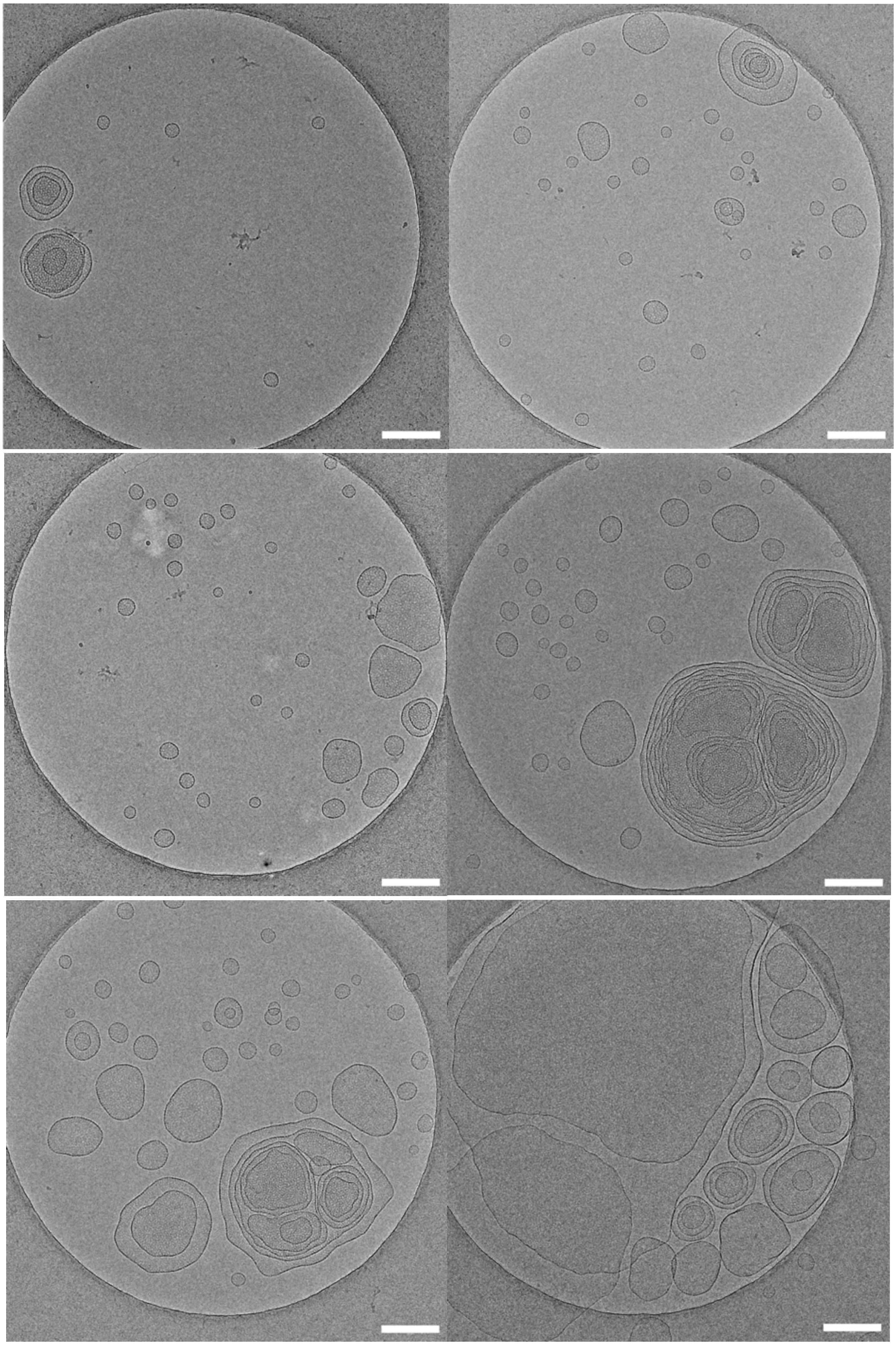

Figure S19: Additional cryo-TEM micrographs for G1HH; scale bar is 200 nm.

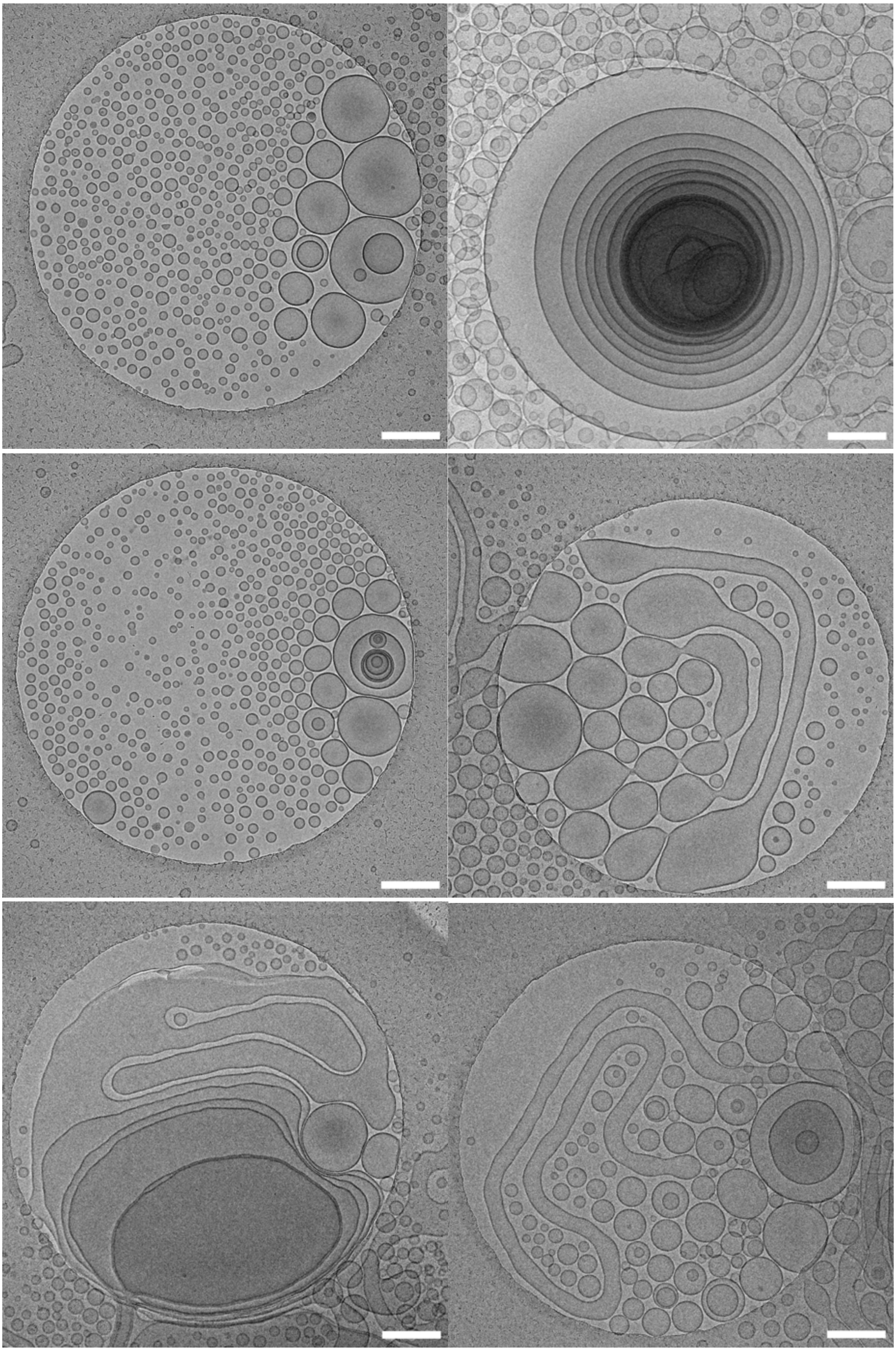

Figure S20: Additional cryo-TEM micrographs for G1FF; scale bar is 200 nm.

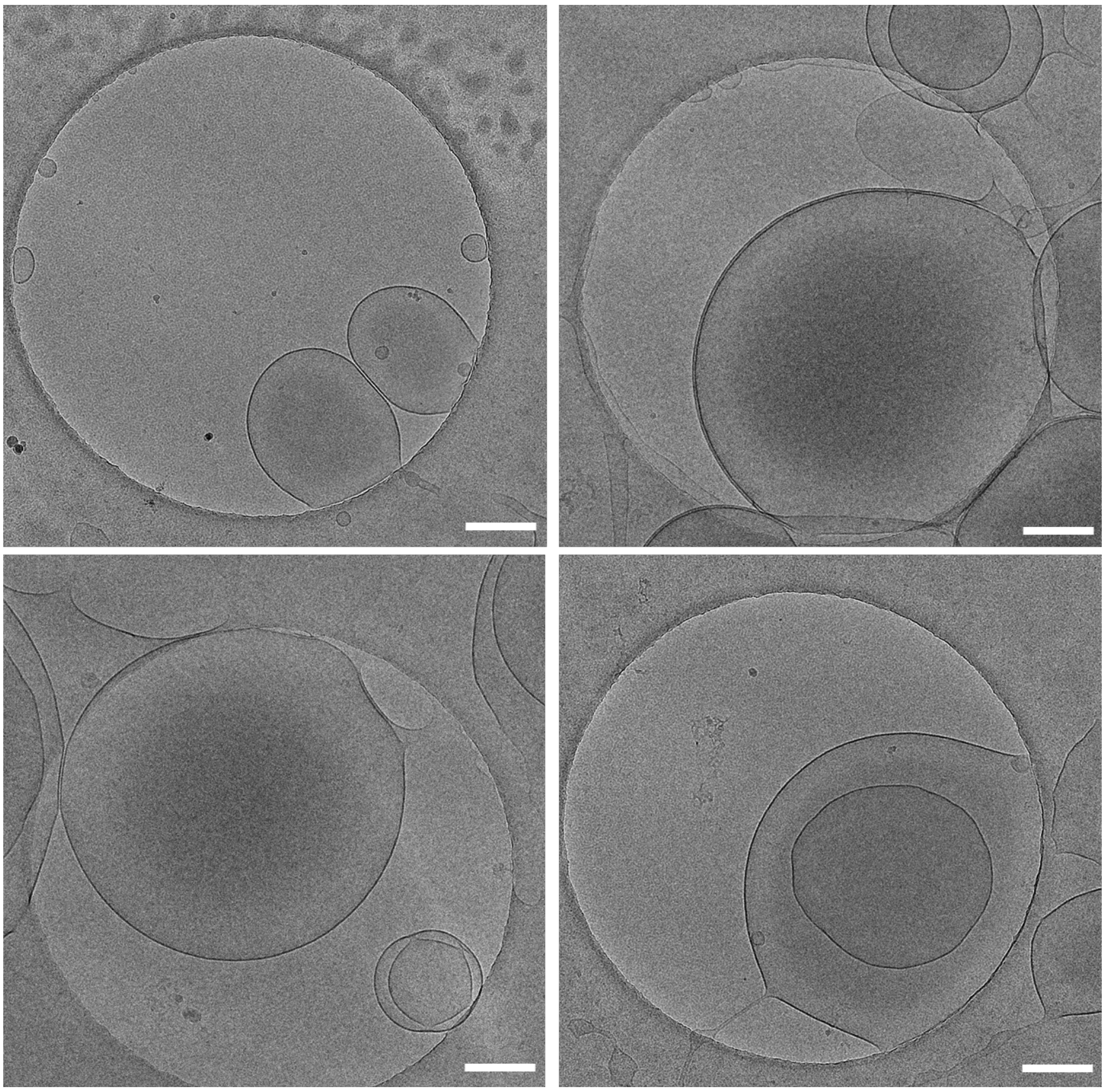

Figure S21. Cryo-TEM micrograph of self-assembled G1HF in water (7 mM). G1HF forms vesicular structures, in agreement with the coarse-grained prediction for G1-generation amphiphiles. Scale bar is 200 nm.

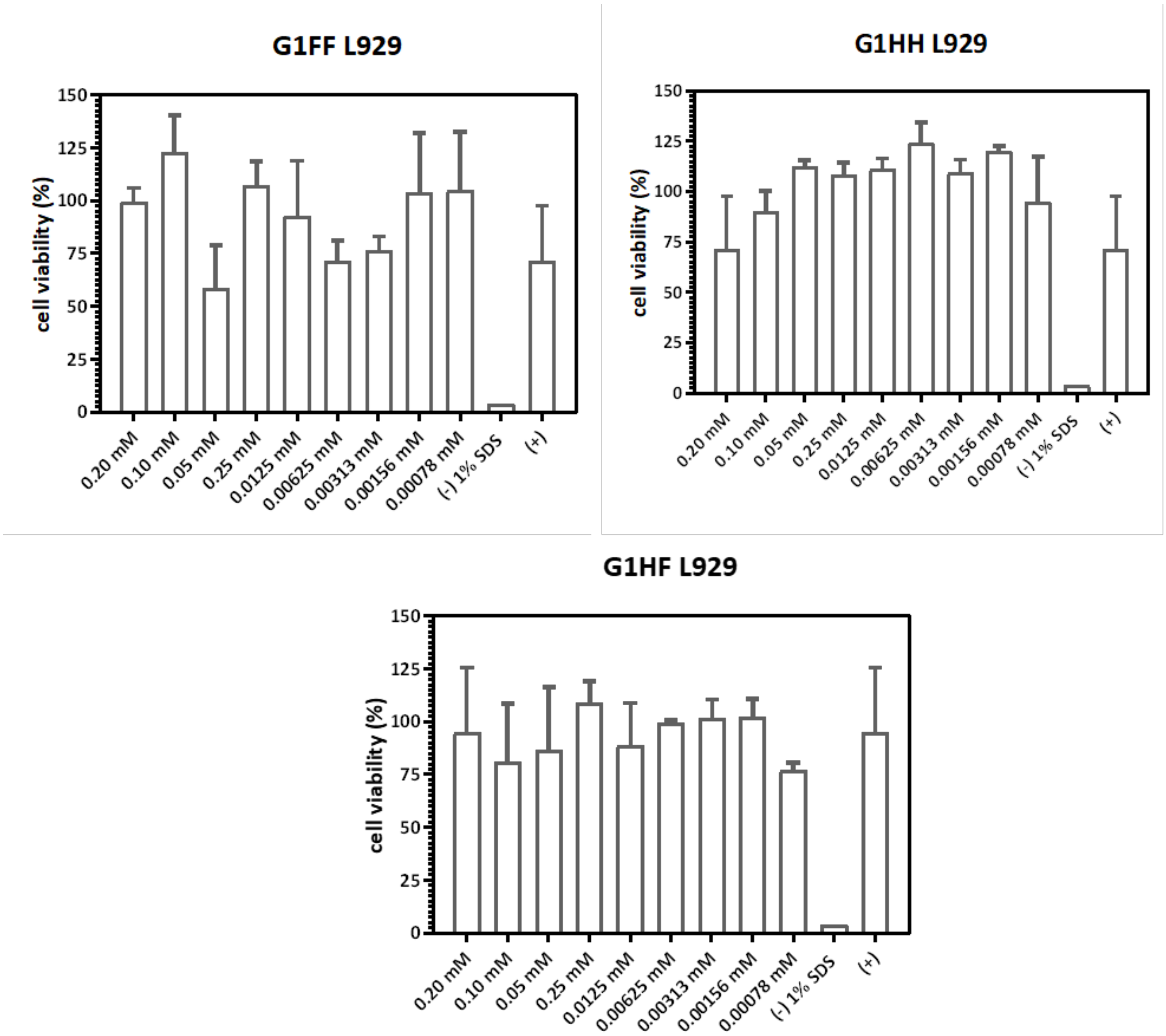

Figure S22. Cytotoxicity assessment of G1FF, G1HH, and G1HF using the normal, fibroblast-like L929 cell line, showing cell viability (%) at different amphiphile concentrations. PBS and SDS (1%) served as negative and positive controls, respectively (mean ± SD, n = 3).

S12.1. Determination of amphiphile CMC

- **Method**

The CMC was determined by the fluorescence method using Nile red dye. A stock solution of the dye in THF (5 mg ml^{-1}) was prepared. Each 20 µL of this stock solution was added to 10 sample vials where the THF was allowed to evaporate to leave a thin-film of the dye. Stock solutions of the amphiphiles in water were stirred at least for 1 h before their serial dilutions to the final sample concentrations. The amphiphile solutions (500 µl) were then transferred to the vials containing dye thin-films and the resulting mixture was stirred overnight. Finally, non-encapsulated dye was removed by filtration through 0.45 µm PTFE filter. Fluorescence measurements were performed using a Tecan Spark® plate reader. To graphically determine the CMC, the fluorescence intensity maximum was plotted against the logarithm of the amphiphile concentration. Two sections of the graph were chosen based on when an increase in fluorescence intensity was observed, and both sections were analysed by linear regression. The CMC is the concentration at the intersection.

- **Data analysis and results**

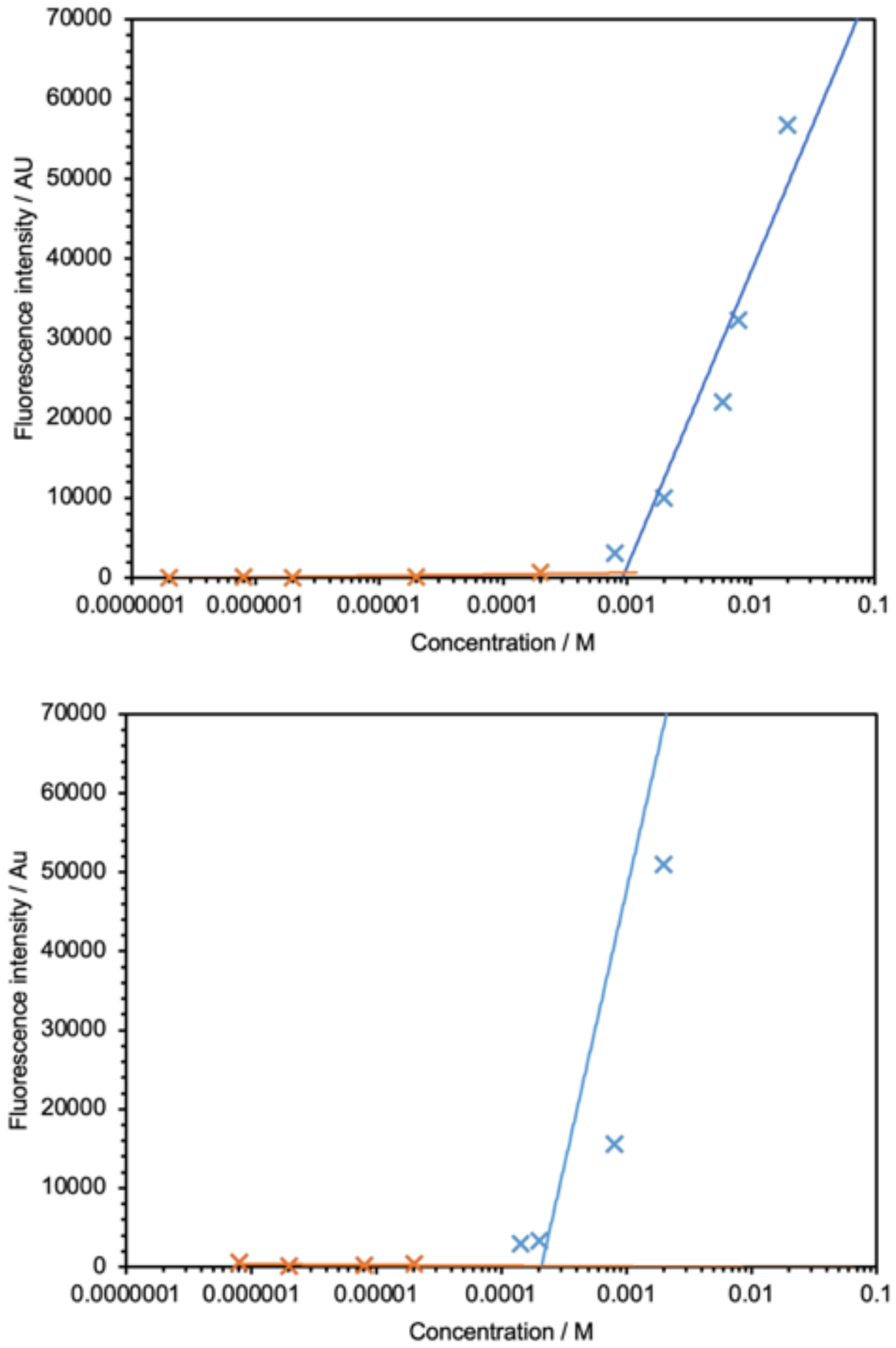

Figure S23: CMC determination of G1HH (left, CMC = 0.96 ± 0.23 mM) and G1FF (right, CMC = 0.16 ± 0.06 mM).

S13. Membrane interaction experiments

- **Labelling procedure of amphiphile with hydrophilic dye ICC-COOH**

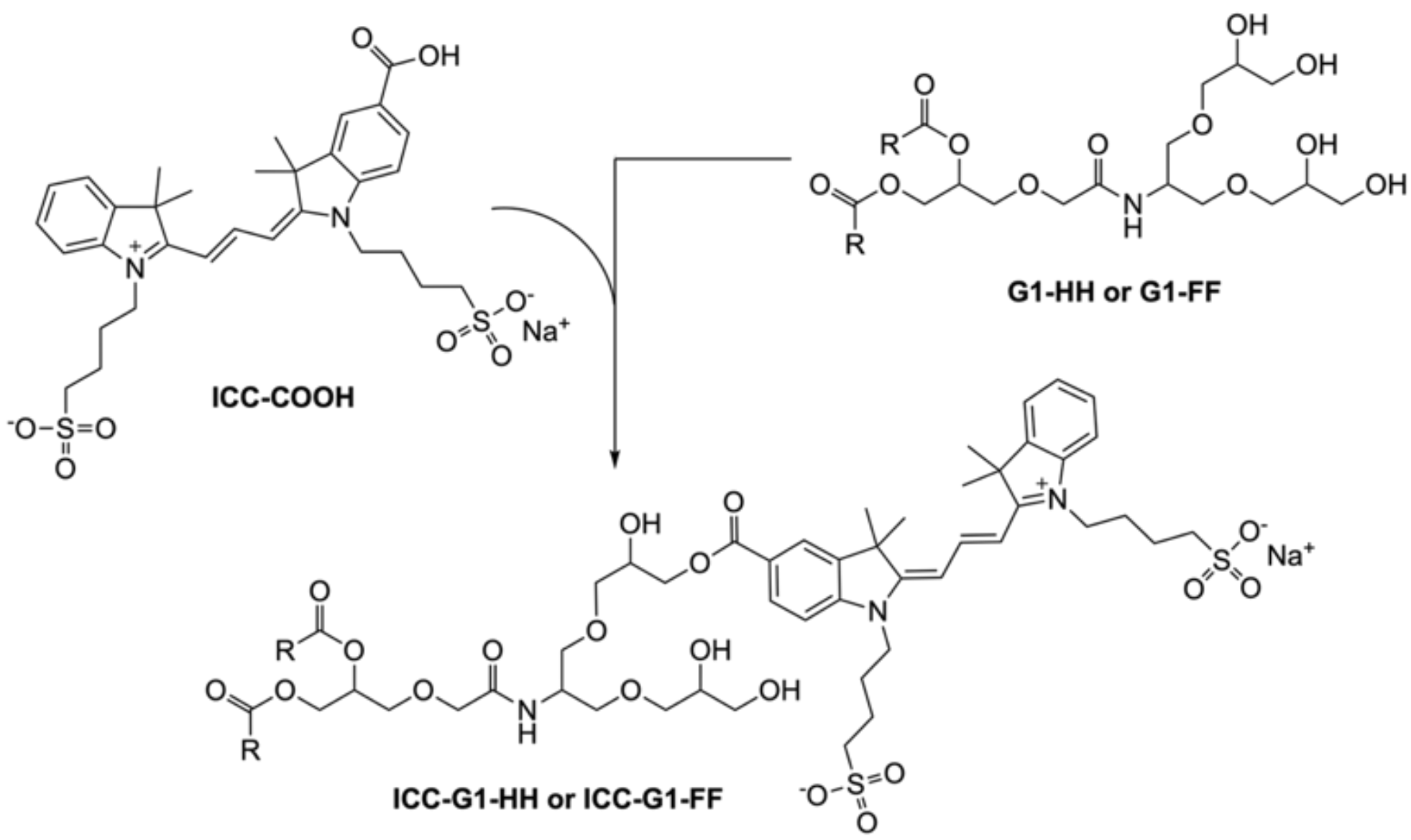

Figure S24: Synthesis of dye labelled amphiphiles.

The synthetic procedure and subsequent purification method was adapted from the literature [38-42].

ICC-G1HH: In a flask under argon were dissolved ICC-COOH (0.69 mg, 0.00104 mmol, 1.0 eq.), DMAP (0.3 mg, 0.00207 mmol, 2 eq.) and EDC·HCl (0.6 mg, 0.00311 mmol, 3 eq.) in anhydrous DMF (10 ml) and stirred at 0 °C for 30 min. A solution of G1HH (2.52 mg, 0.00387 mmol, 3.7 eq.), in anhydrous DMF (5 ml) was added and the resulting mixtures was heated to 35 °C and stirred for 48 h. Successful reaction was initially tested with a small-scale separation using Sephadex® G-25 Superfine in a glass pipette, and the rapid collection of the dye indicated a successful reaction. The absence of free dye was further verified by RP-TLC comparing the uncoupled dye ICC-COOH and the product ICC-G1HH (80% MeOH/H_2O, R_f = 0 for ICC-COOH and 0.56 for ICC-G1HH; traces of $(ICC)_2$-G1HH were found at R_f = 0.29). The DMF in the rest of the reaction mixture was removed by rotary evaporation at 60 °C, redissolved in H_2O and purified using Sephadex® G-25 Superfine, this time on a large-scale. The product was concentrated by rotary evaporation and ICC-G1HH was obtained. The product was diluted to achieve a total concentration of 1 mM in water and characterised by UV-Vis spectroscopy (λ_{max} = 550 nm, **1 : 0.021 amphiphile to dye ratio**) then used for cellular uptake experiments. Fluorescence measurements demonstrated that the ICC dye kept its fluorescence activity intact after conjugation.

ICC-G1FF: In a flask under argon were dissolved ICC-COOH (0.97 mg, 0.0015 mmol, 1 eq.), DMAP (0.4 mg, 0.0029 mmol, 2 eq.) and EDC·HCl (0.8 mg, 0.0044 mmol, 3 eq.) in anhydrous DMF (10 ml) and stirred at 0 °C for 30 min. A solution of G1FF (6.84 mg, 0.0061 mmol, 4.2 eq.) in anhydrous DMF (5 ml) was added and the resulting mixtures was heated to 35 °C and stirred for 48 h. Successful reaction was tested with a small-scale separation using Sephadex® G-25 Superfine in a glass pipette, and the rapid collection of the dye indicated a successful reaction. The absence of free dye was further verified by RP-TLC comparing the uncoupled dye ICC-COOH and the product ICC-G1FF (80% MeOH/H_2O, R_f = 0 for ICC-COOH and 0.67 for ICC-G1FF; traces of $(ICC)_2$-G1FF were found at R_f = 0.4). The DMF in the rest of the reaction mixture was removed by rotary evaporation at 60 °C, redissolved in H_2O and purified

using Sephadex® G-25 Superfine, this time on a large-scale. The product was concentrated by rotary evaporation and ICC-G1FF was obtained. The product was diluted to achieve a total concentration of 1 mM in water and characterised by UV-Vis spectroscopy (λ_{max} = 550 nm, **1 : 0.020 amphiphile to dye ratio**) then used for cellular uptake experiments. Fluorescence measurements demonstrated that the ICC dye kept its fluorescence activity intact after conjugation.

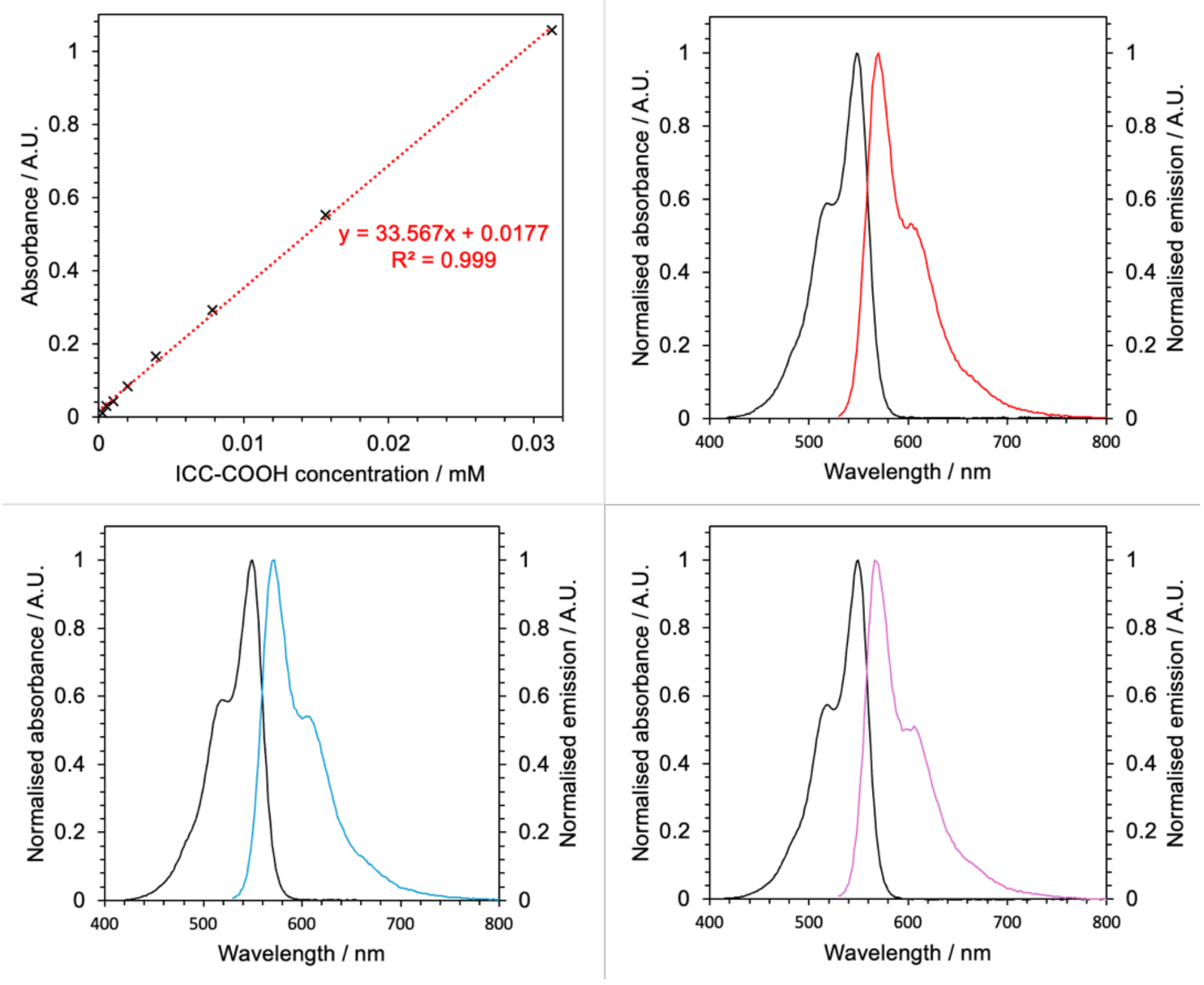

Figure S25: (Top left) The calibration curve of ICC-COOH in water used to determine the amphiphile to dye ratio in samples for cellular uptake experiments. For the ICC-COOH samples, pure water was used as a background sample, while for the amphiphile solutions, an unlabelled amphiphile solution of the same concentration was used. Additionnally, spectra were corrected to account for Rayleigh scattering; (red) absorbance and emission spectra for ICC-G1HH in water (λ_{max}(abs) = 548 nm, λ_{max}(em) = 570 nm) ; (blue) absorbance and emission spectra for ICC-G1FF in water (λ_{max}(abs) = 550 nm, $\lambda_{max,}$(em) = 572 nm); (violet) absorbance and emission spectra for ICC-COOH in water (λ_{max}(abs) = 550 nm, $\lambda_{max,}$(em) = 566 nm).

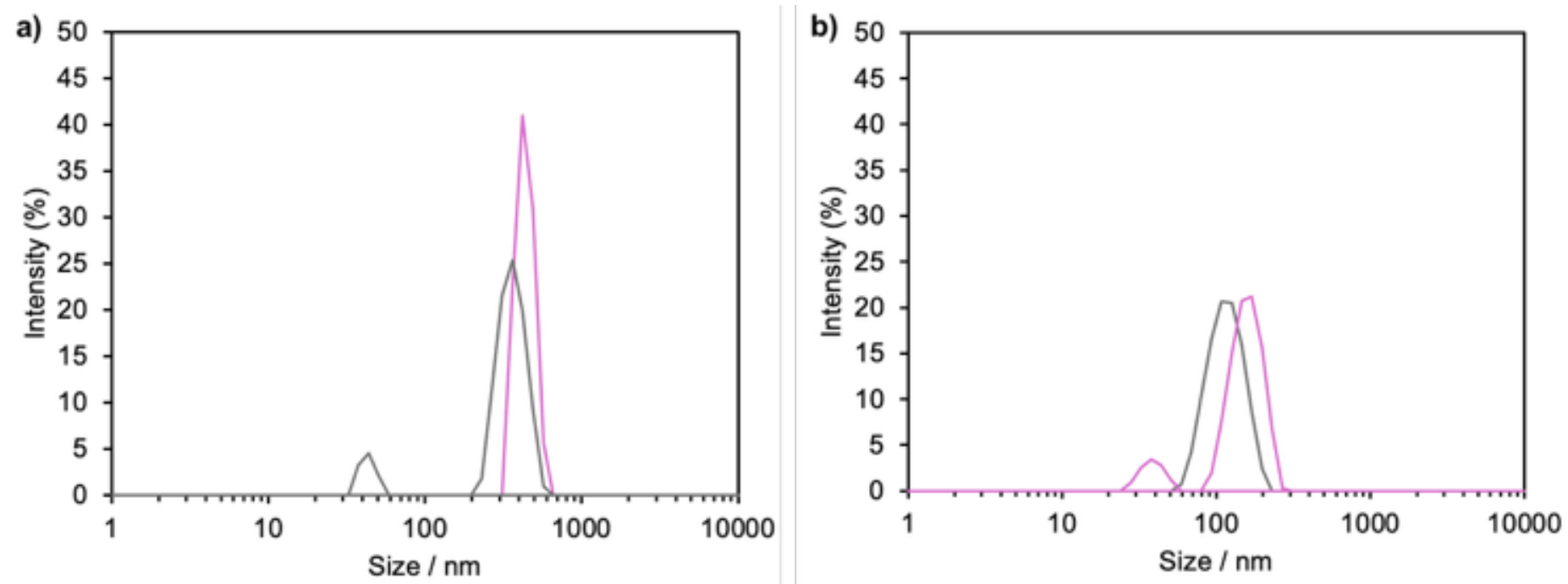

Figure S26: a) DLS plot of G1FF before (grey) and after (violet) dye labelling; b) G1HH before (grey) and after (violet) dye labelling.

- **Cellular uptake**

The amphiphile cellular uptake in HeLa cancer cell line [(DSMZ no.: ACC 57)] was monitored using confocal laser scanning microscopy (cLSM). The cells were routinely cultivated. For cLSM, 400 µl of cells in DMEM [(1X) (Gibco, Ref. No.: 31966-021) + 10% fetal bovine serum (FBS) (Sigma-Aldrich, Co., Cat. No.: F7524) + 1% penicillin-streptomycin (PS) (Sigma-Aldrich, Co., Cat. No.: P4333)] with a cell

density of 5 x10^4 cells ml^{-1} were seeded in each well of an 8-well μ-slide [8 Well ibiTreat (Cat.No: 80806)]. After incubation over night at 37°C and 5.00 % CO_2, labelled amphiphile aqueous solution was added at a final test concentration of either 0.4, 0.2 or 0.05 mM. As a positive control ICC-COOH aqueous solution at a final test concentration of either 0.008, 0.004 or 0.001 mM was added. After incubation for 4 h the cell nuclei were stained with Hoechst 33342 (Life Technologies GmbH, Darmstadt, Germany). The confocal images were taken by using an inverted confocal laser scanning microscope Leica DMI6000CSB SP8 (Leica, Wetzlar, Germany) with an 63x/1.4 HC PL APO CS2 oil immersion objective using the manufacture given LAS X software.

- **Cytotoxicity assessment**

All experiments were conducted following the German genetic engineering laws and German biosafety guidelines in the laboratory (S2). For determining the cell viability a CCK-8 Kit was used following the manufacturers instructions.

HeLa cells were cultivated in DMEM medium. For cell viability measurements both cell lines were seeded in a 96 well plate at a density of 5x10^4 cells/mL in DMEM-Medium (90.0 μL/well). The seeded cells were incubated over night at 37°C and 5% CO_2. 10.0 μL/well of each compound was applied to the plate including negative (1% SDS) and positive (Medium, 10% DPBS) controls. The cells with compound were incubated for another 24h at 37°C and 5% CO_2. After 24h of incubation 10.0 μL/well of CCK-8 solution was added. After 3h of incubation at 37°C and 5% CO2 the absorbance was measured (450nm/650nm) using a Tecan SPARK Plate Reader.

All measurement were performed with three technical and two biological repeats. The cell viability was calculated by setting the positive control to 100% using the Excel software. All Graphs were plotted using GraphPad Prism 6.

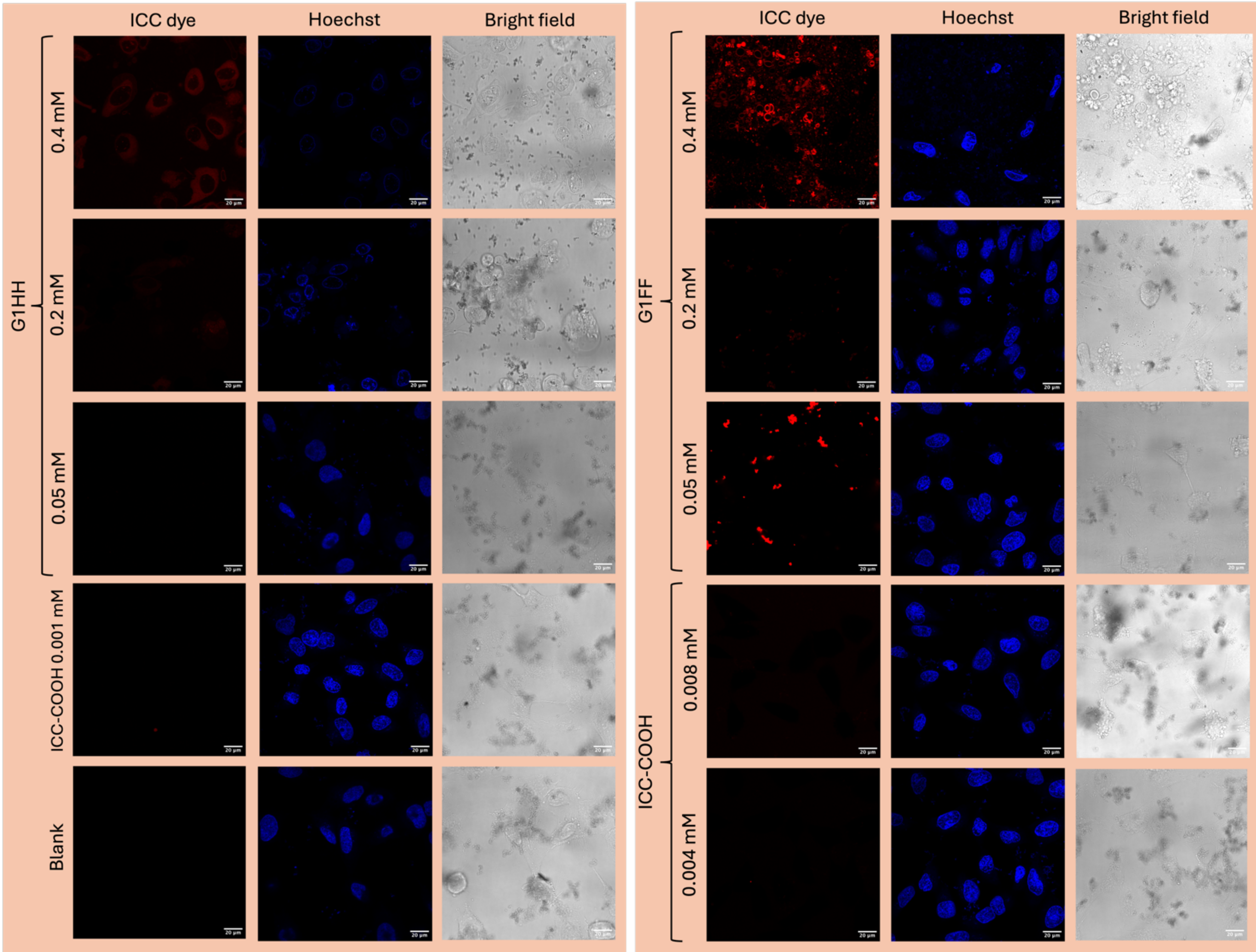

Figure S27: Confocal laser scanning microscopy images of labelled amphiphiles and control samples taken up by HeLa cells after incubation at 37 °C. Scale bar is 20 µm.

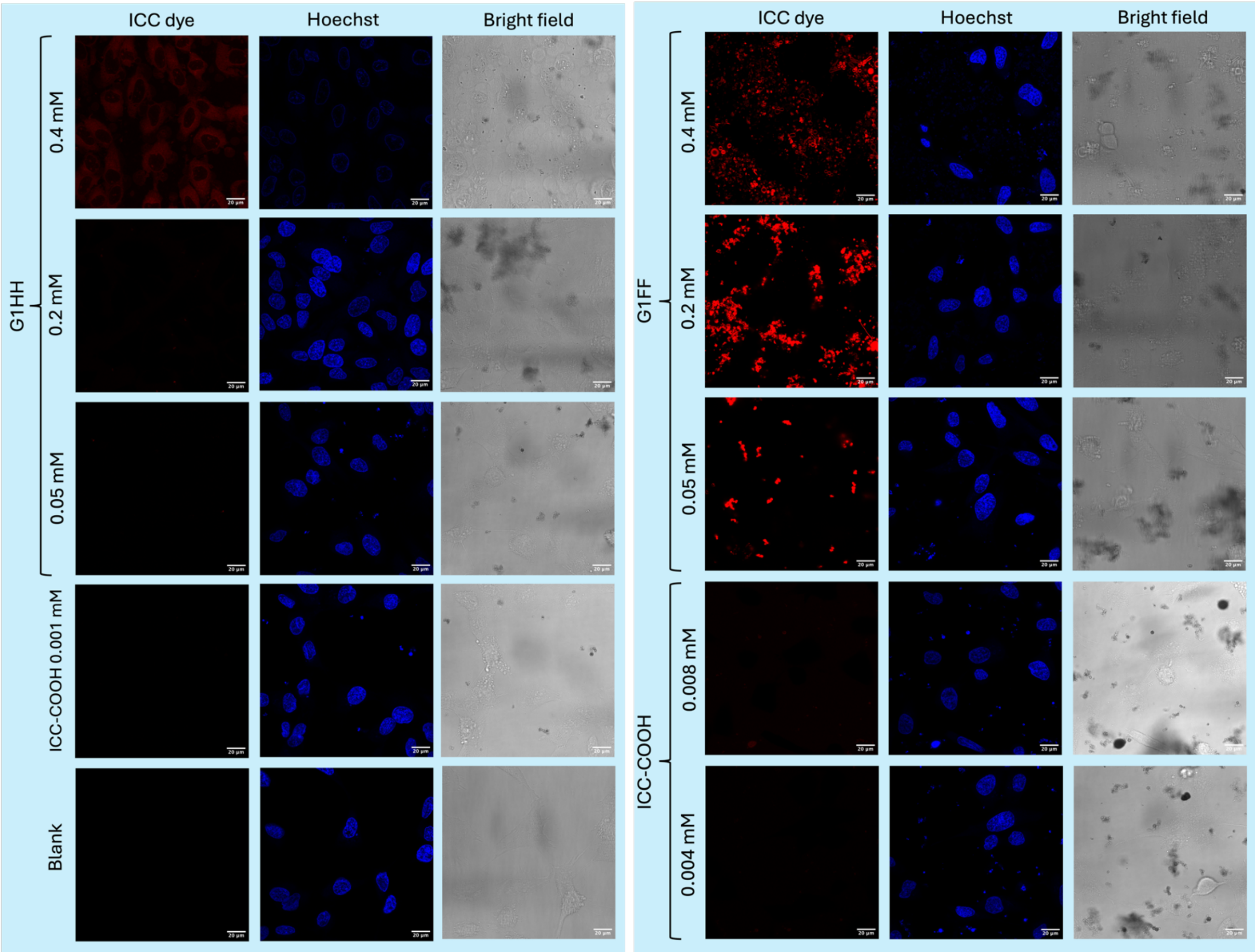

Figure S28: Confocal laser scanning microscopy images of labelled amphiphiles and control samples taken up by HeLa cells after incubation at 4 °C. Scale bar is 20 µm. More sensitive settings were used for G1FF 0.2 mM to make self-assemblies visible, which looked similar to the 37 °C case if consistent settings were used.

G1HF was incubated with HeLa cells at 0.40, 0.20, and 0.05 mM (37 °C) using the same confocal protocol as for G1HH and G1FF. A concentration-dependent, intermediate profile was observed: extracellular aggregates dominated at 0.40 mM, intracellular uptake of individual molecules was seen at 0.05 mM, and both coexisted at 0.20 mM (Figure S29).

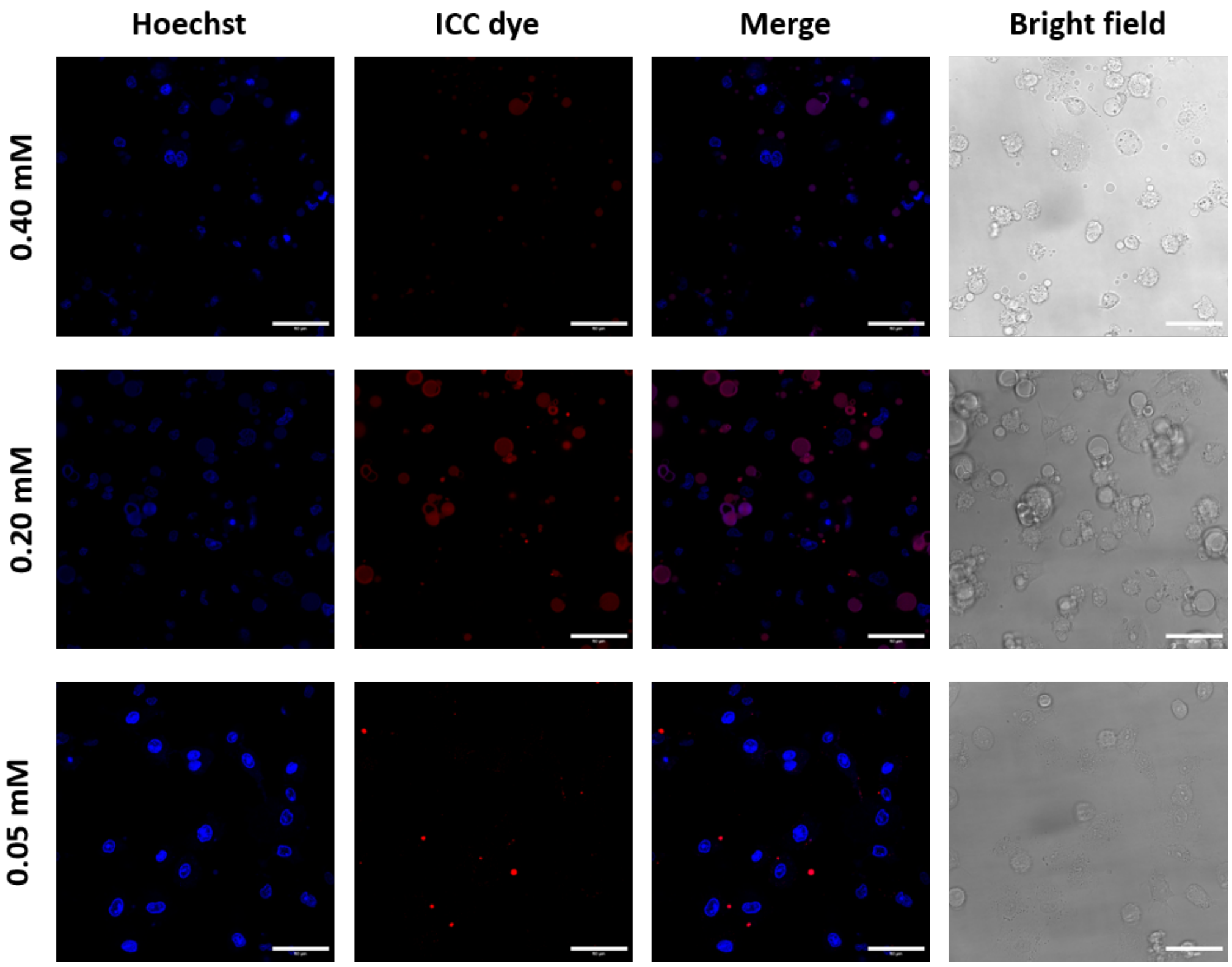

Figure S29: Confocal laser scanning microscopy images of ICC labelled G1HF (0.40 mM, 0.20 mM and 0.05 mM) taken up by HeLa cells after incubation at 37 °C. Scale bar is 50 μm.

S14. Copies of NMR spectra

- **Compound 1**

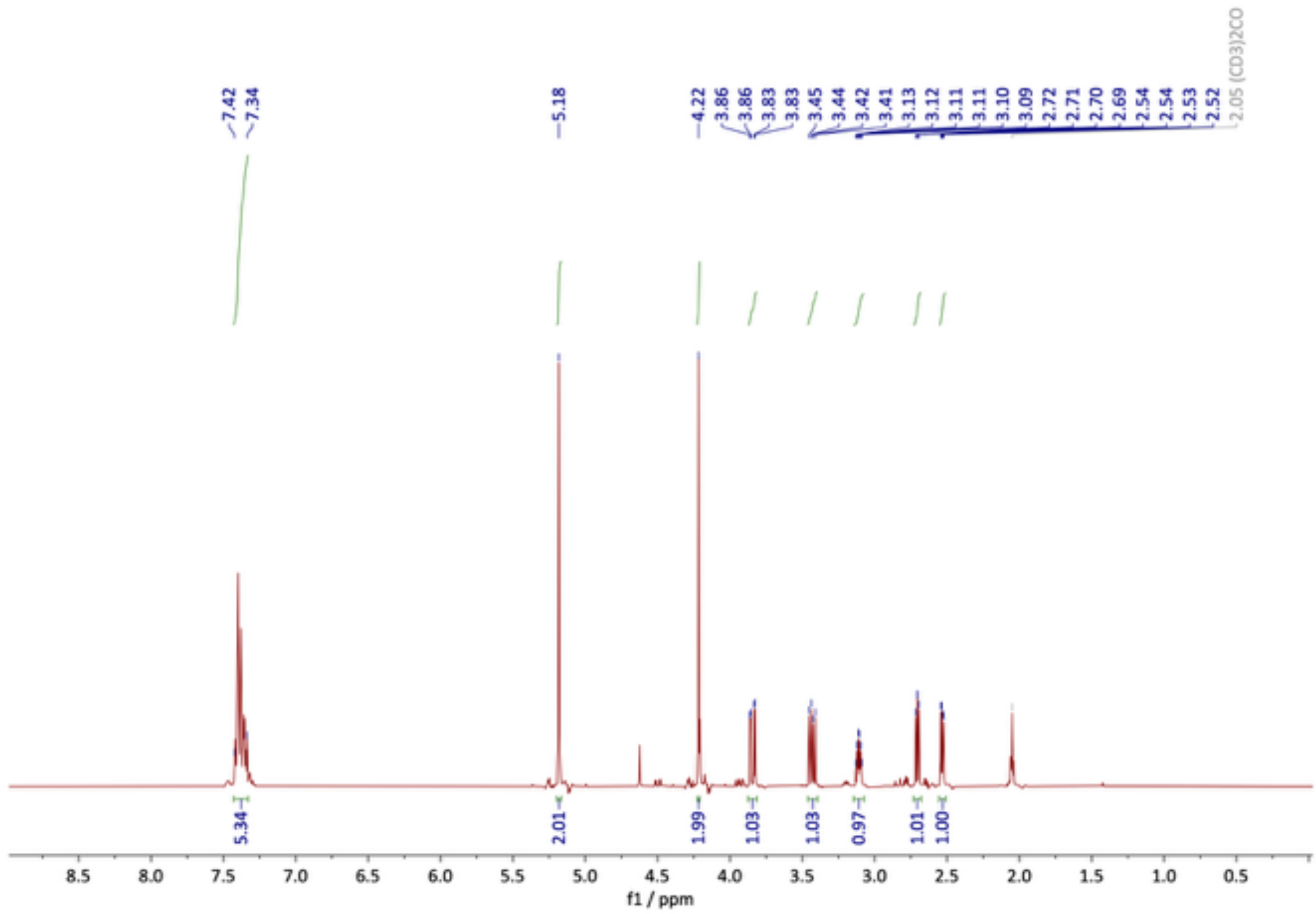

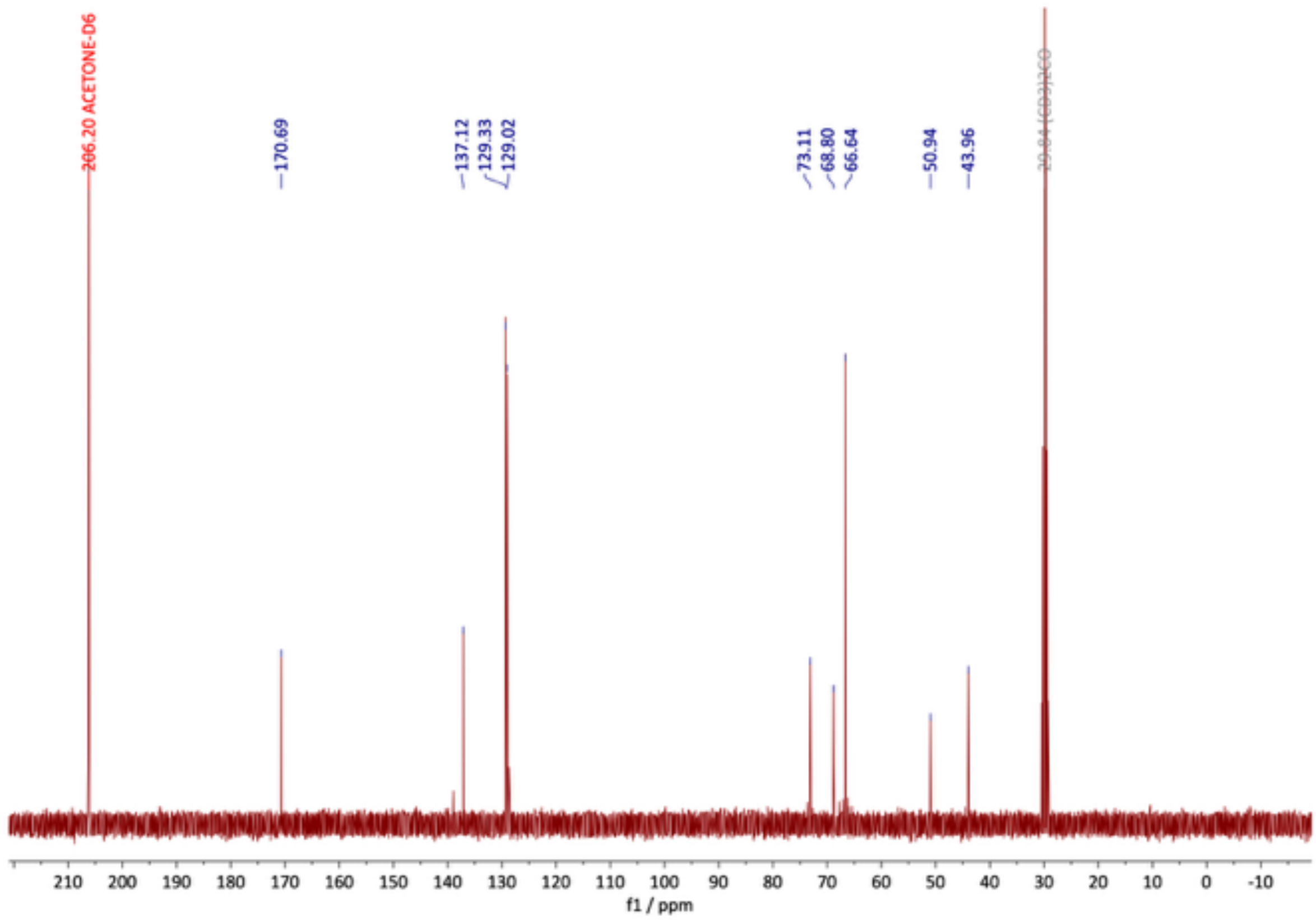

- **Compound 2**

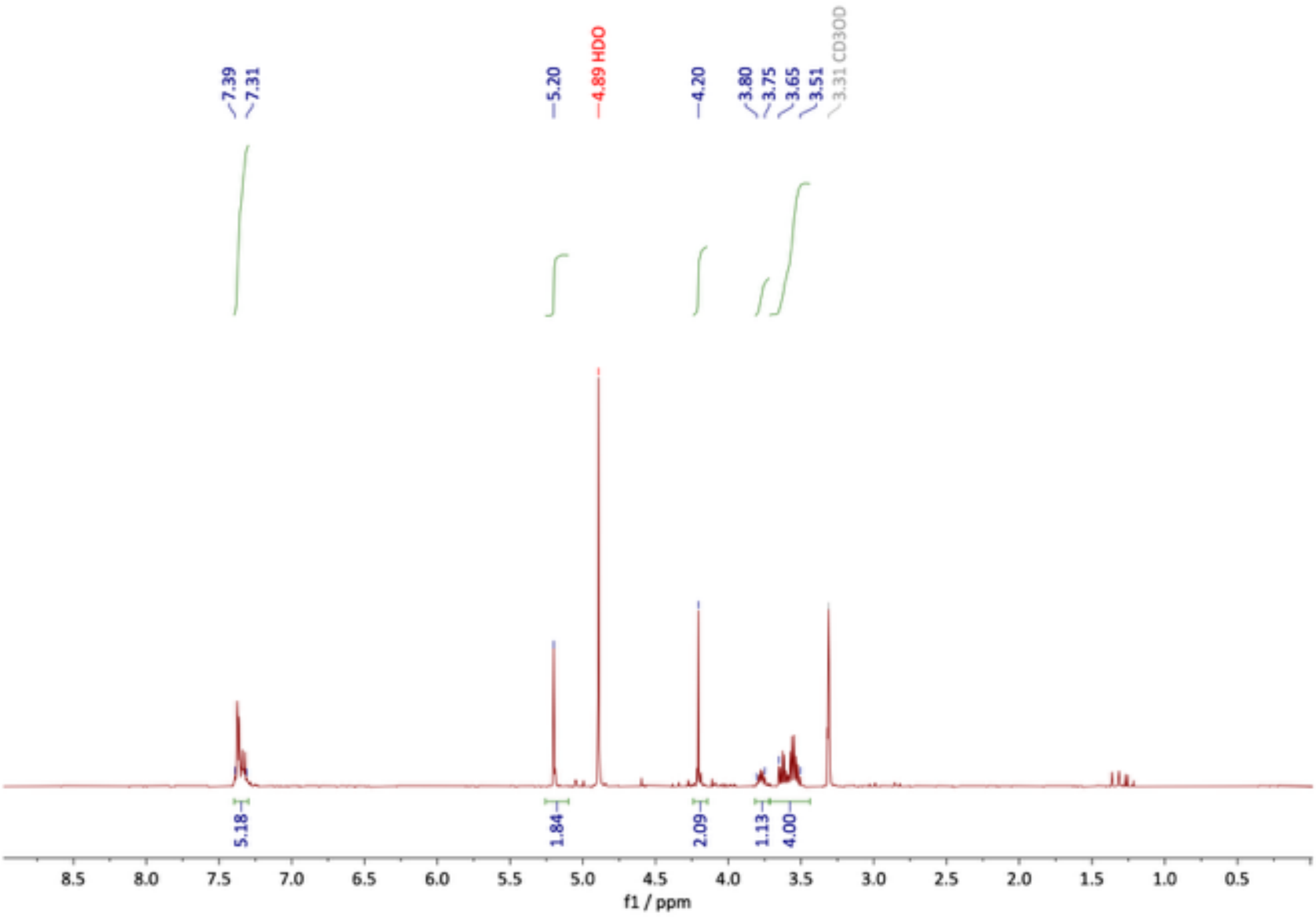
7.39
7.31
5.20
4.89 HDO
4.20
3.80
3.75
3.65
3.51
3.31 CD3OD
5.18
1.84
2.09
1.13
4.00
8.5
8.0
7.5
7.0
6.5
6.0
5.5
5.0
4.5
4.0
3.5
3.0
2.5
2.0
1.5
1.0
0.5
f1 / ppm

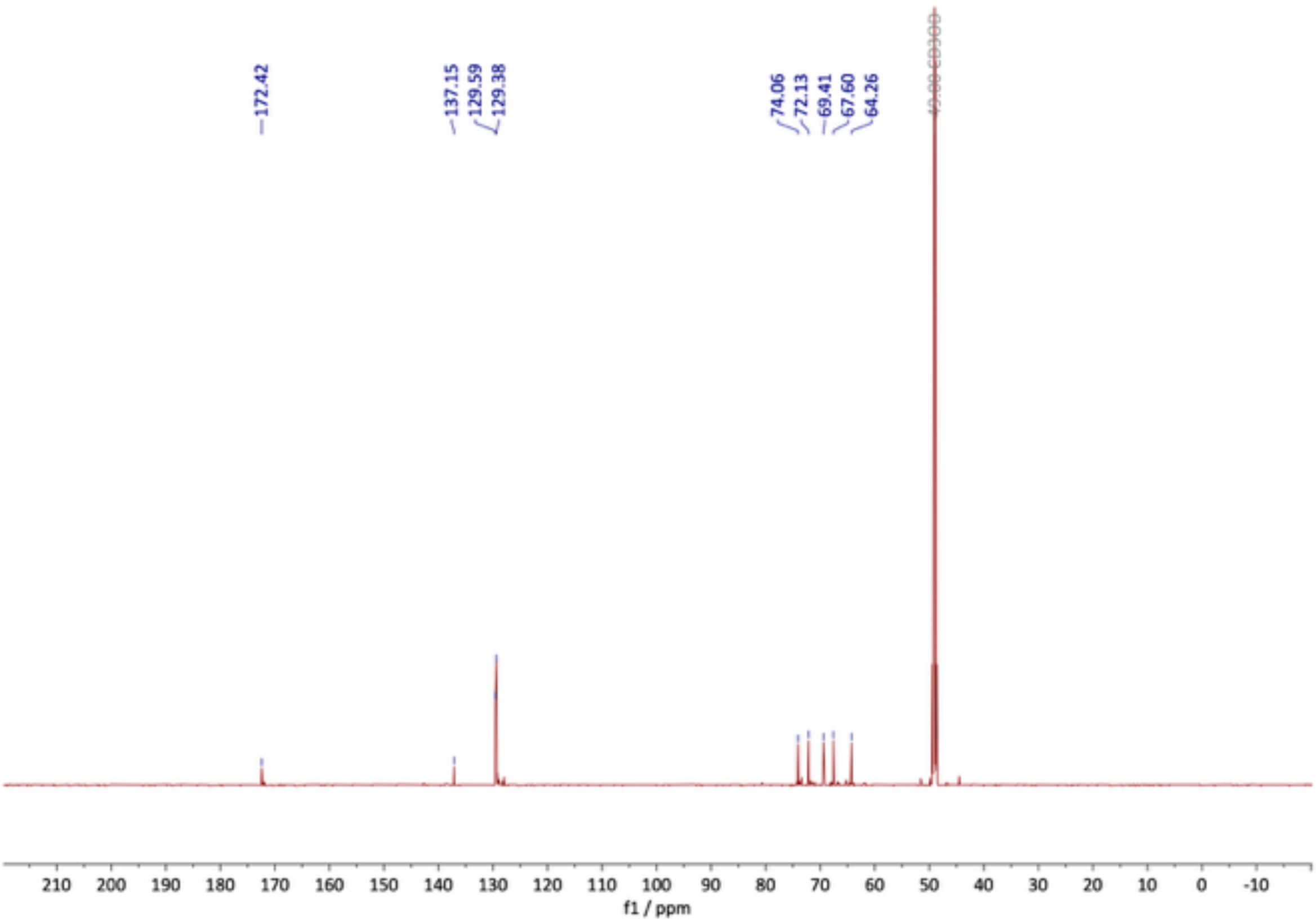
172.42
137.15
129.59
129.38
74.06
72.13
69.41
67.60
64.26
210
200
190
180
170
160
150
140
130
120
110
100
90
80
70
60
50
40
30
20
10
0
-10
f1 / ppm

- **Compound 3-HH**

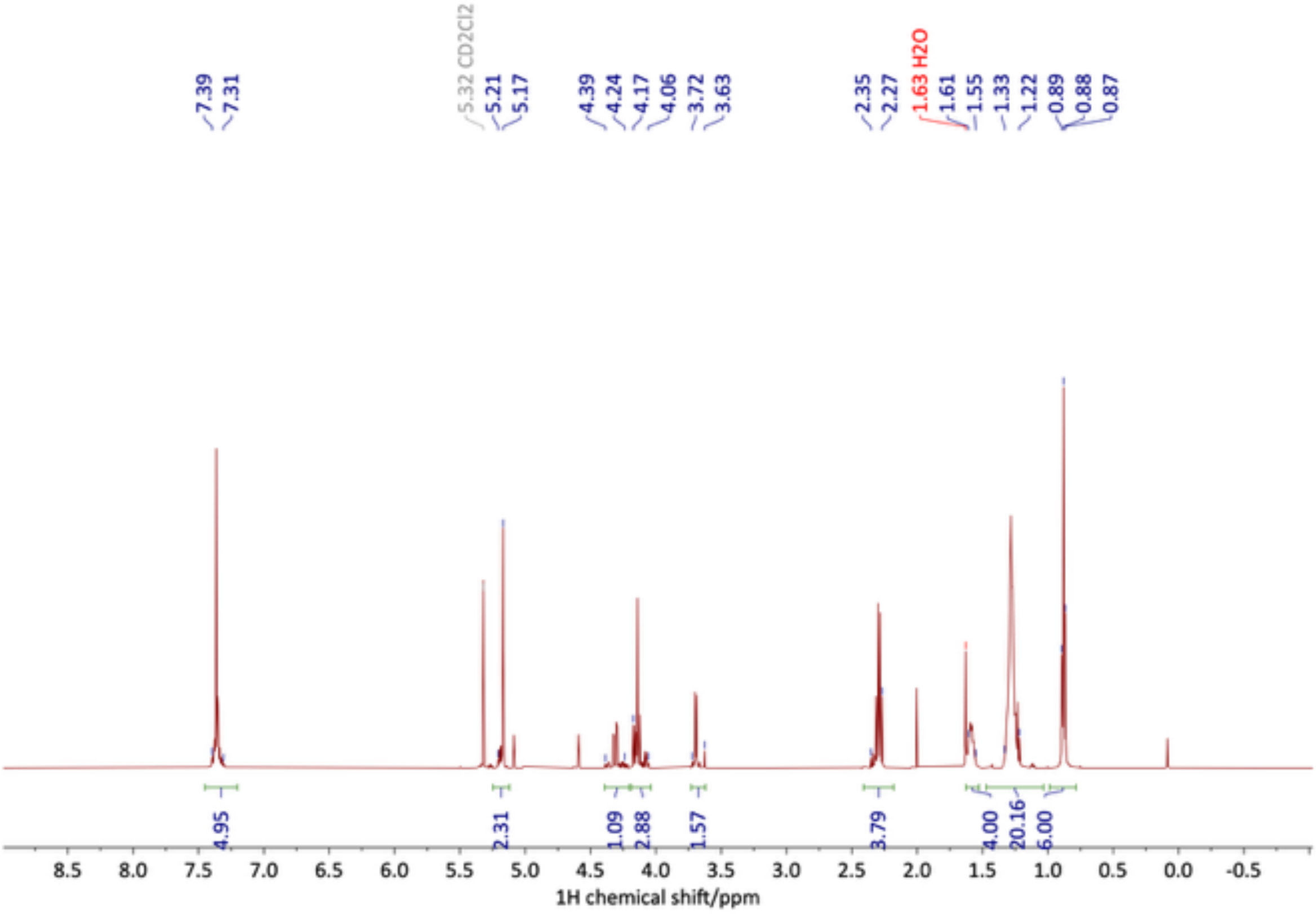

- **Compound 3-FF**

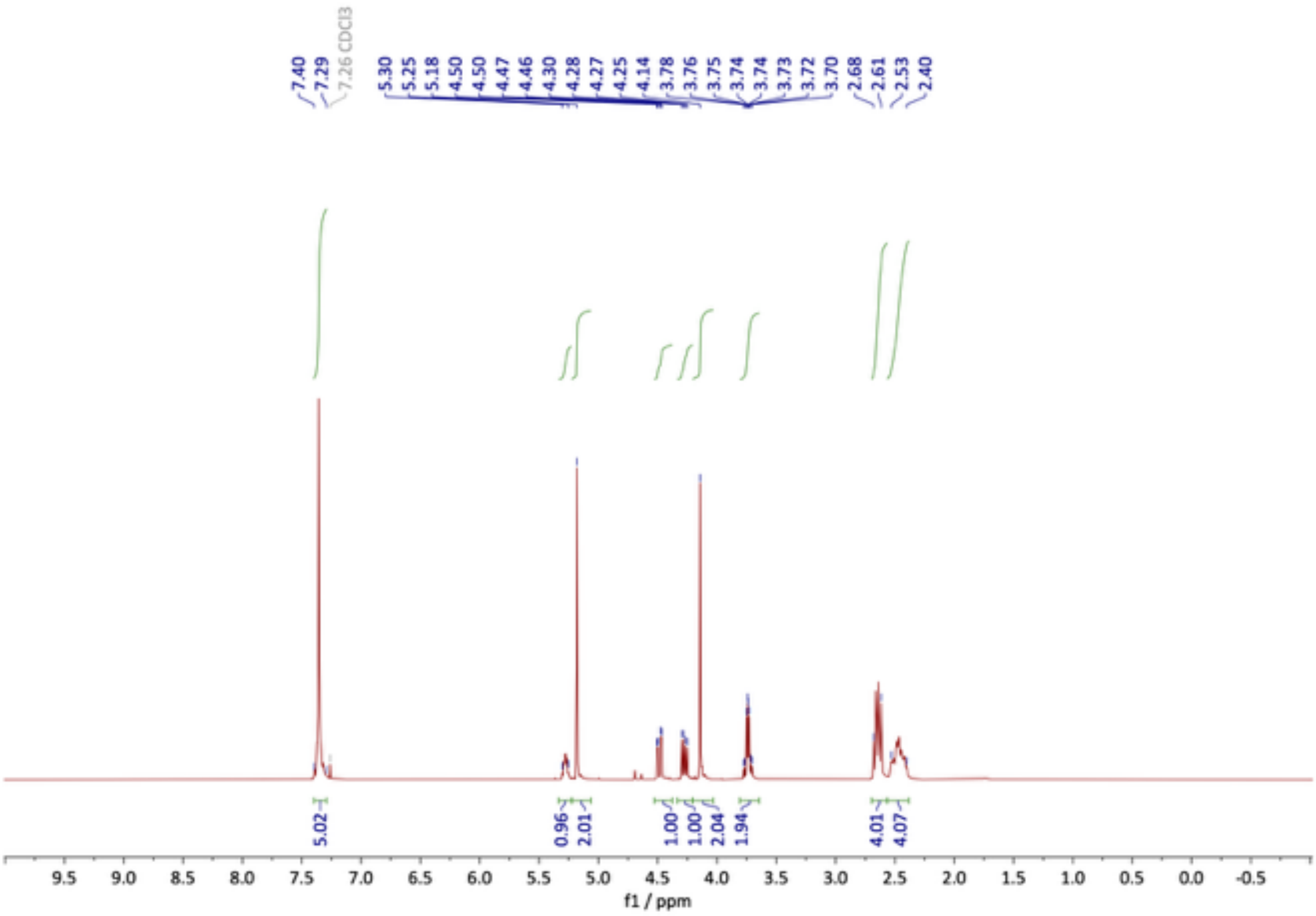

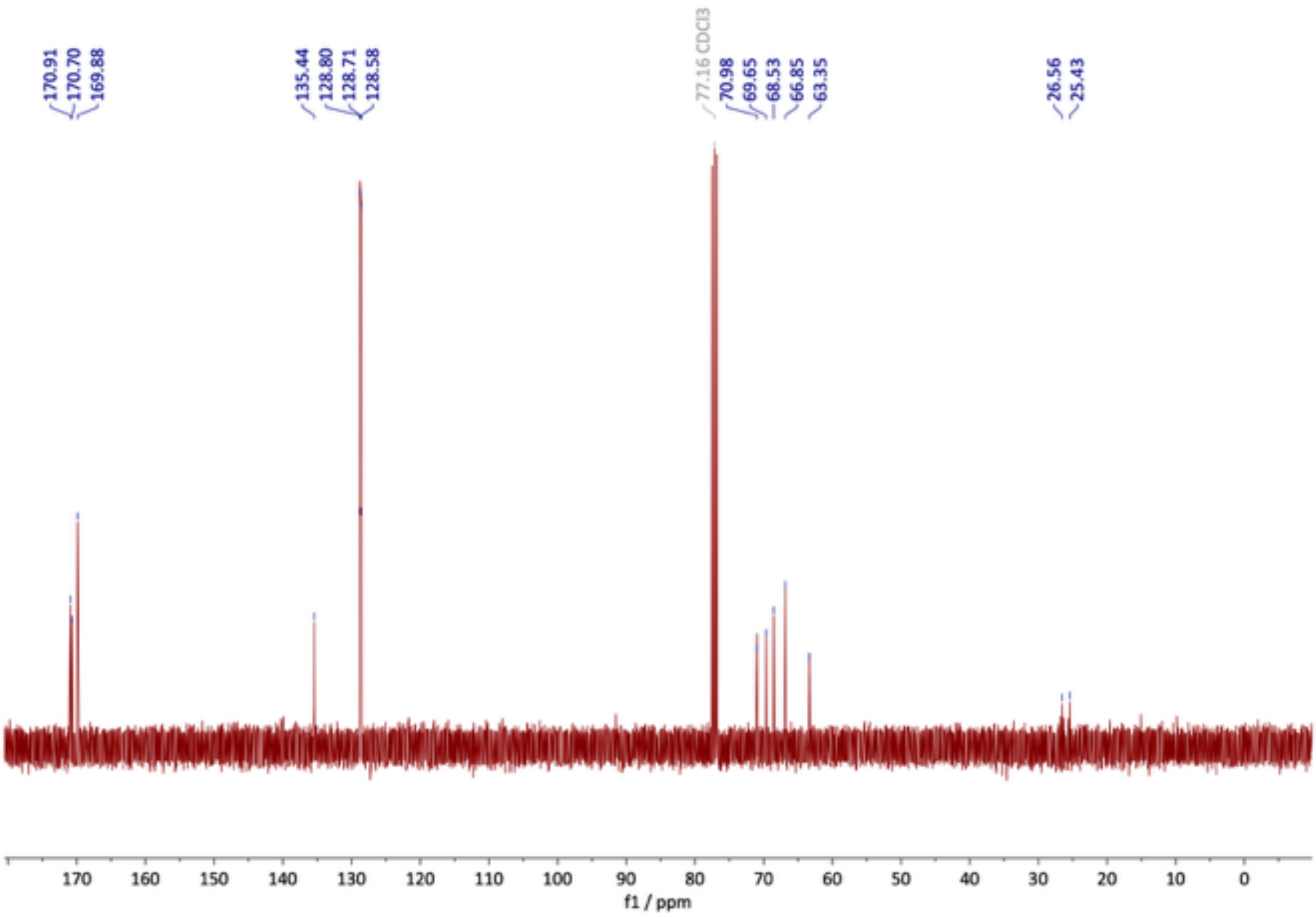

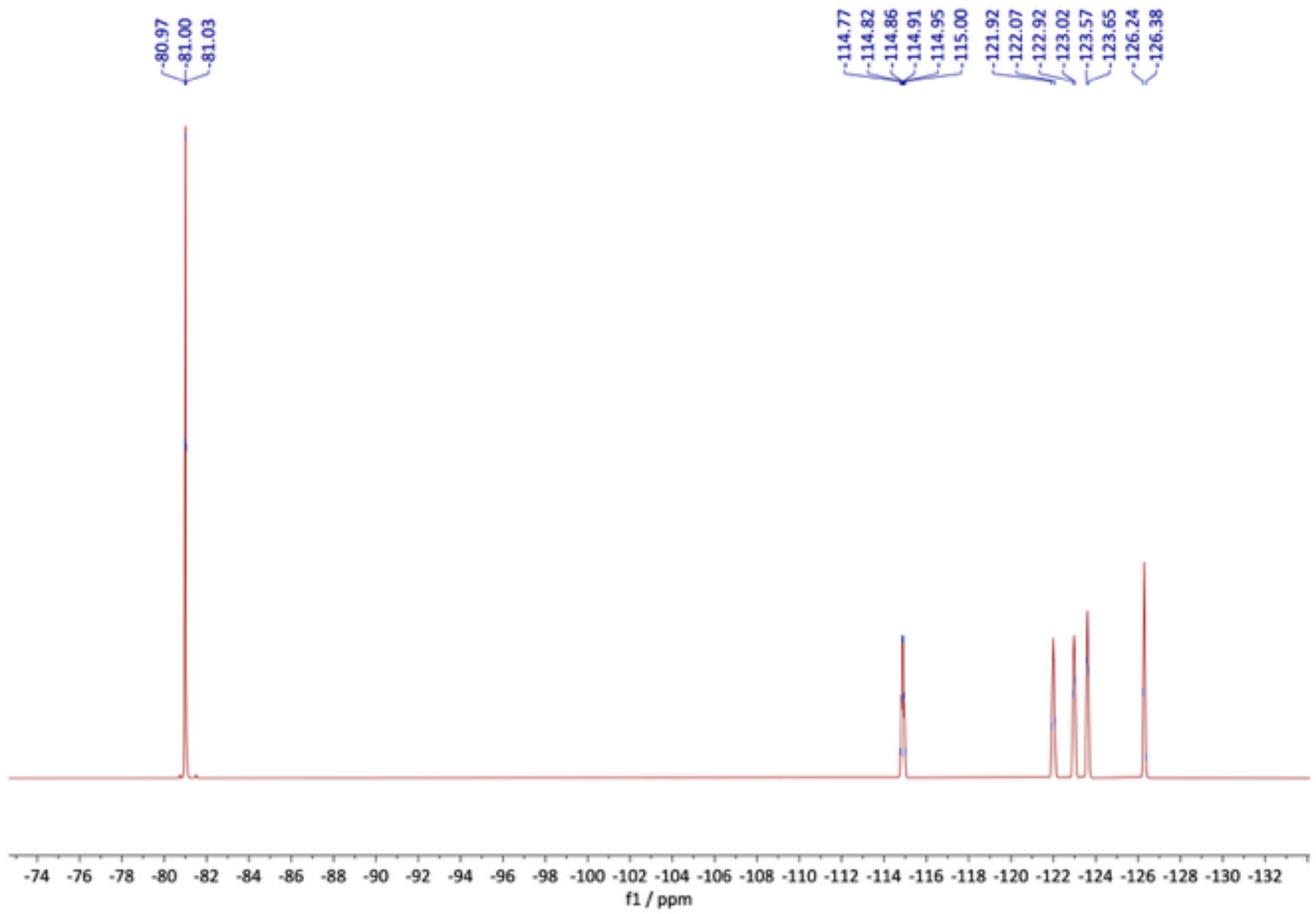
-80.97
-81.00
-81.03
-114.77
-114.82
-114.86
-114.91
-114.95
-115.00
-121.92
-122.07
-122.92
-123.02
-123.57
-123.65
-126.24
-126.38
-74 -76 -78 -80 -82 -84 -86 -88 -90 -92 -94 -96 -98 -100 -102 -104 -106 -108 -110 -112 -114 -116 -118 -120 -122 -124 -126 -128 -130 -132
f1 / ppm

- **Compound 4-HH**

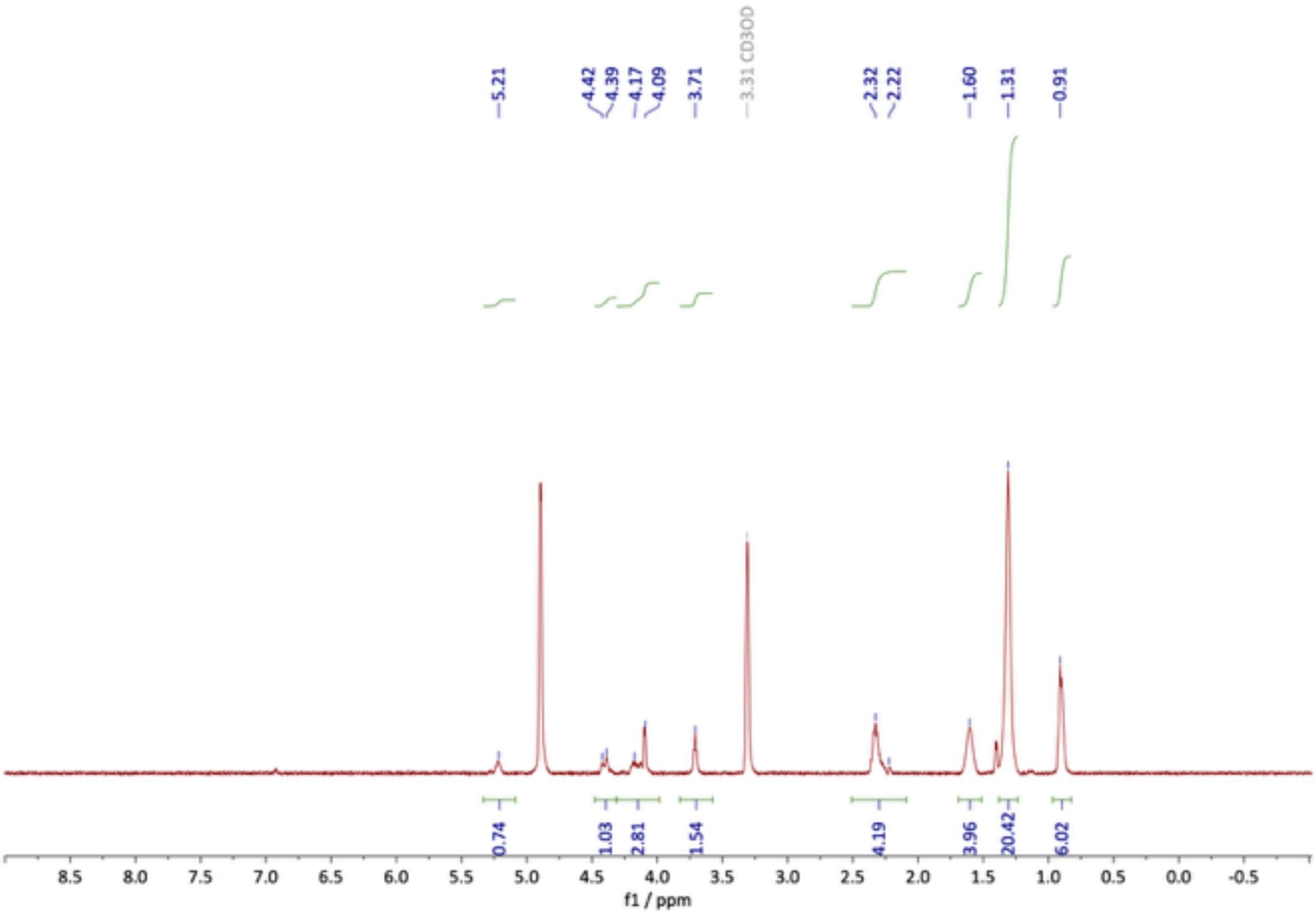

- **Compound 4-FF**

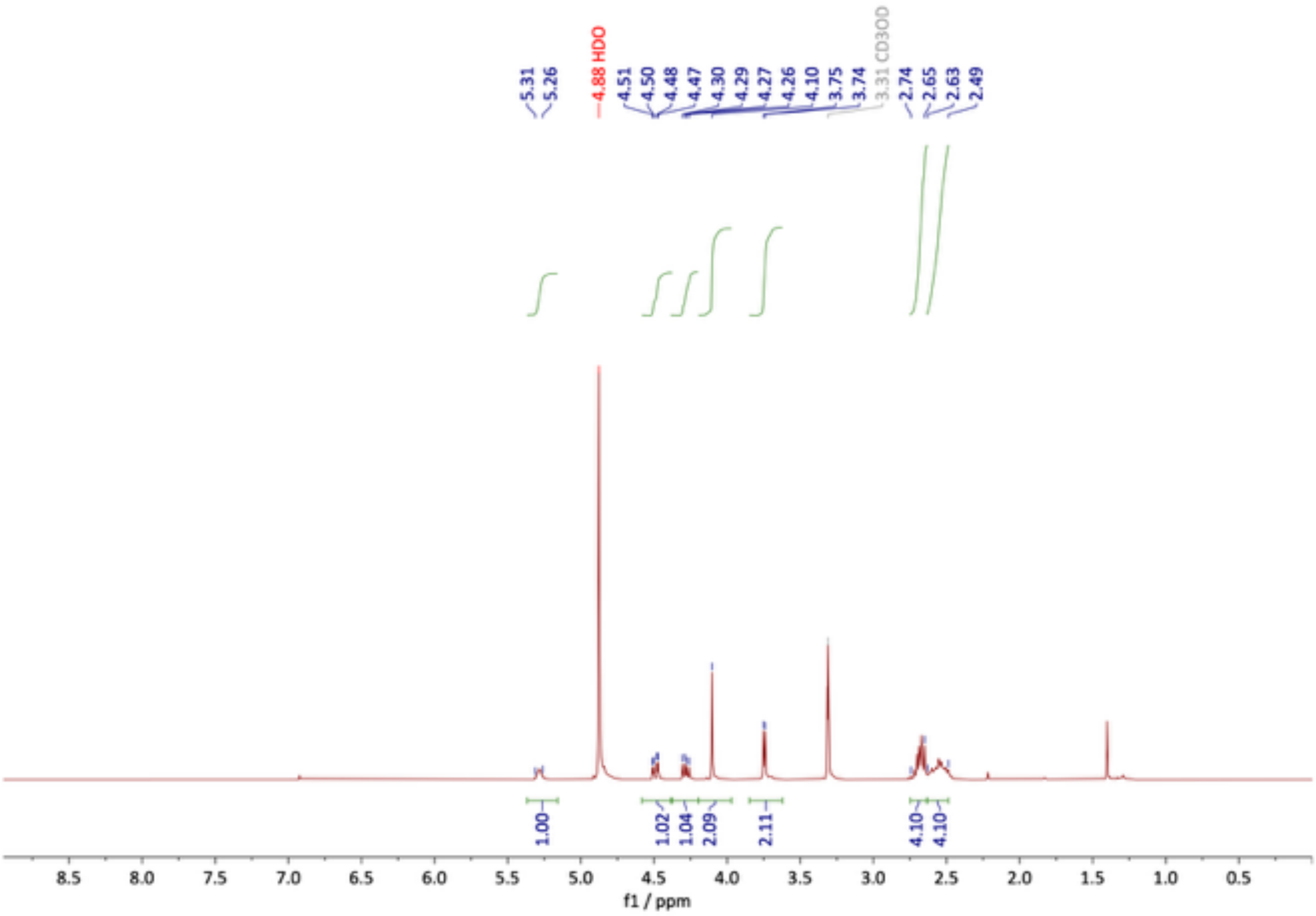

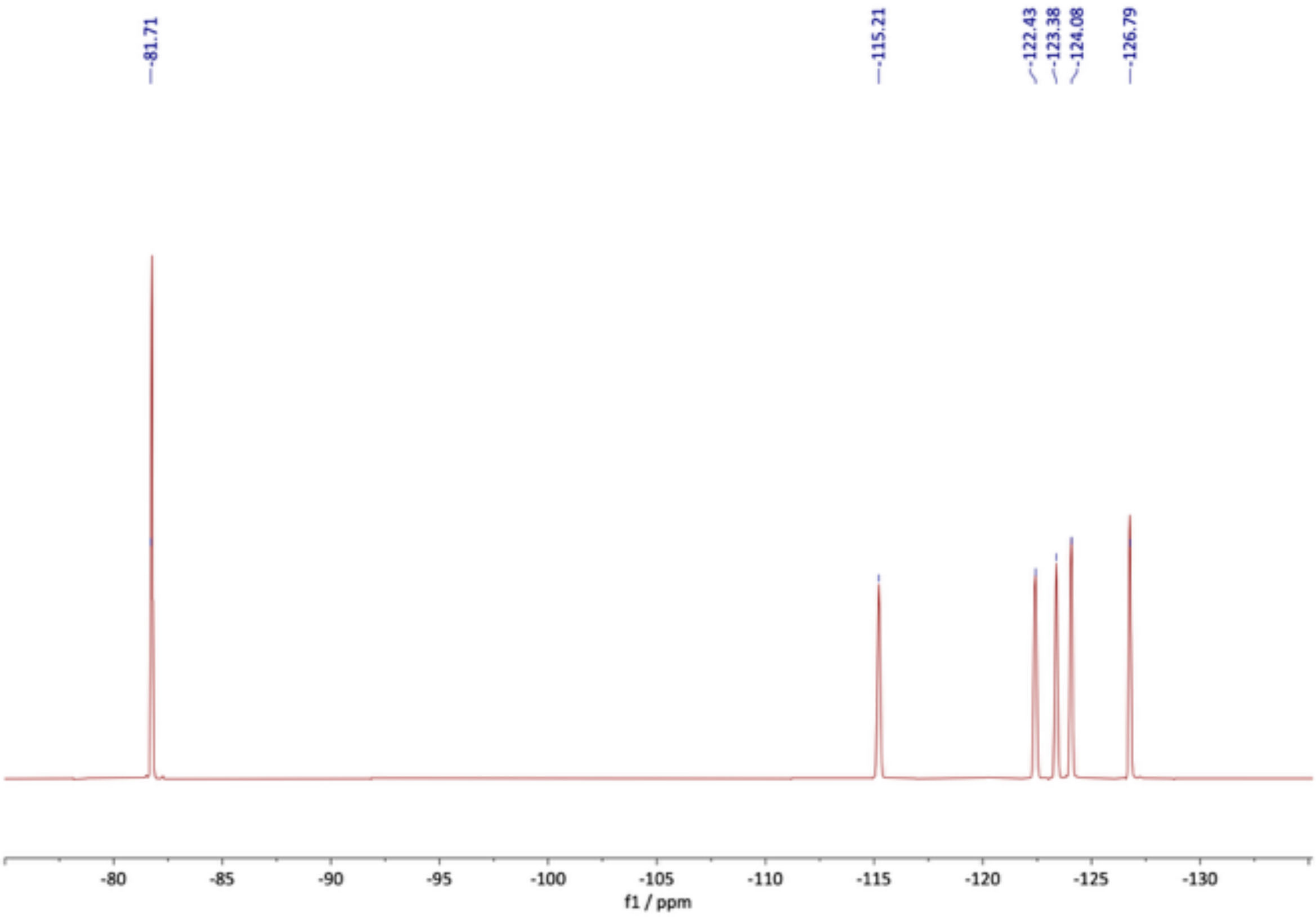

- **pG1HH**

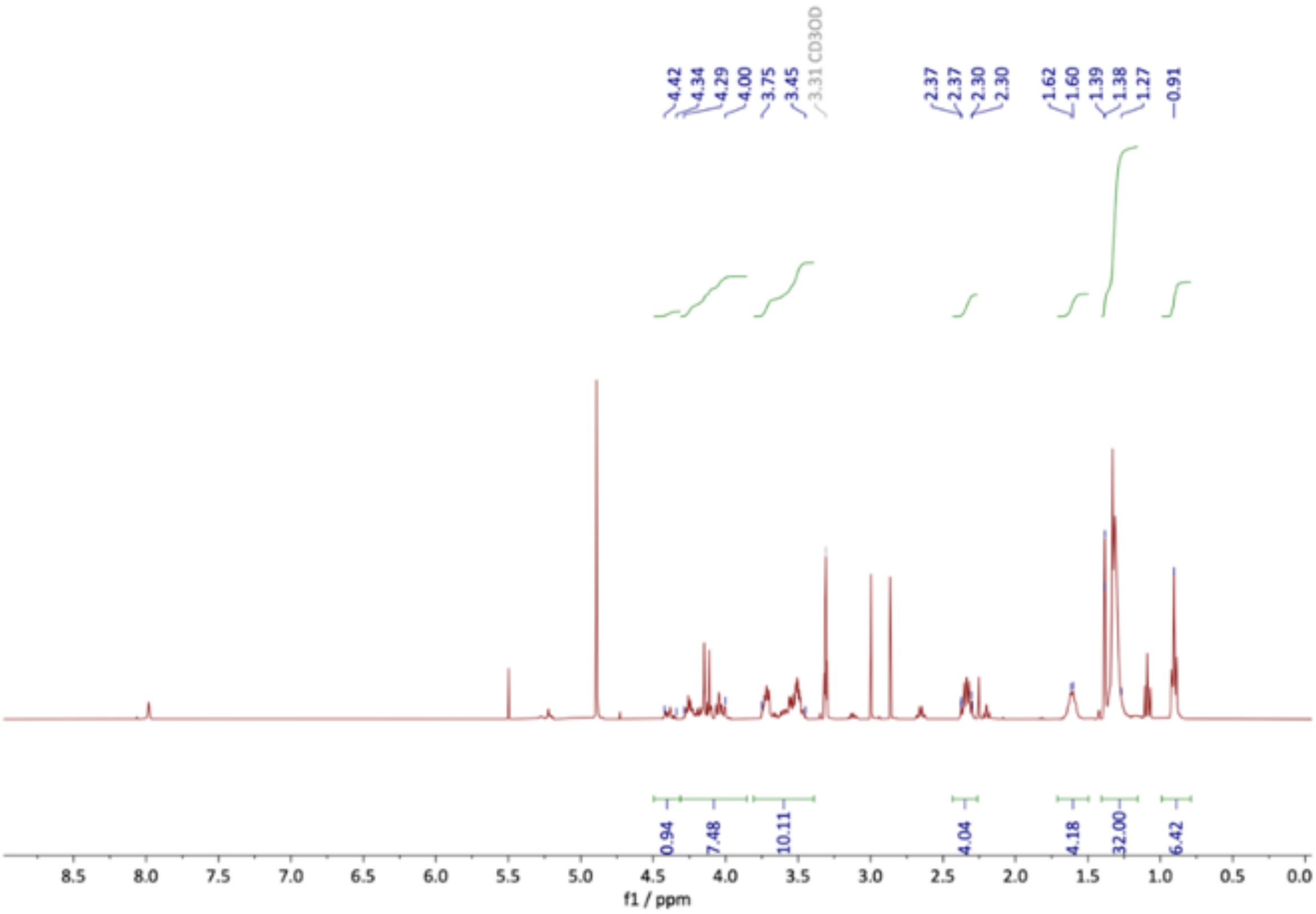

4.42
4.34
4.29
4.00
3.75
3.45
3.31 CD3OD
2.37
2.37
2.30
2.30
1.62
1.60
1.39
1.38
1.27
0.91
0.94
7.48
10.11
4.04
4.18
32.00
6.42
8.5
8.0
7.5
7.0
6.5
6.0
5.5
5.0
4.5
4.0
3.5
3.0
2.5
2.0
1.5
1.0
0.5
0.0
f1 / ppm

- **pG1FF**

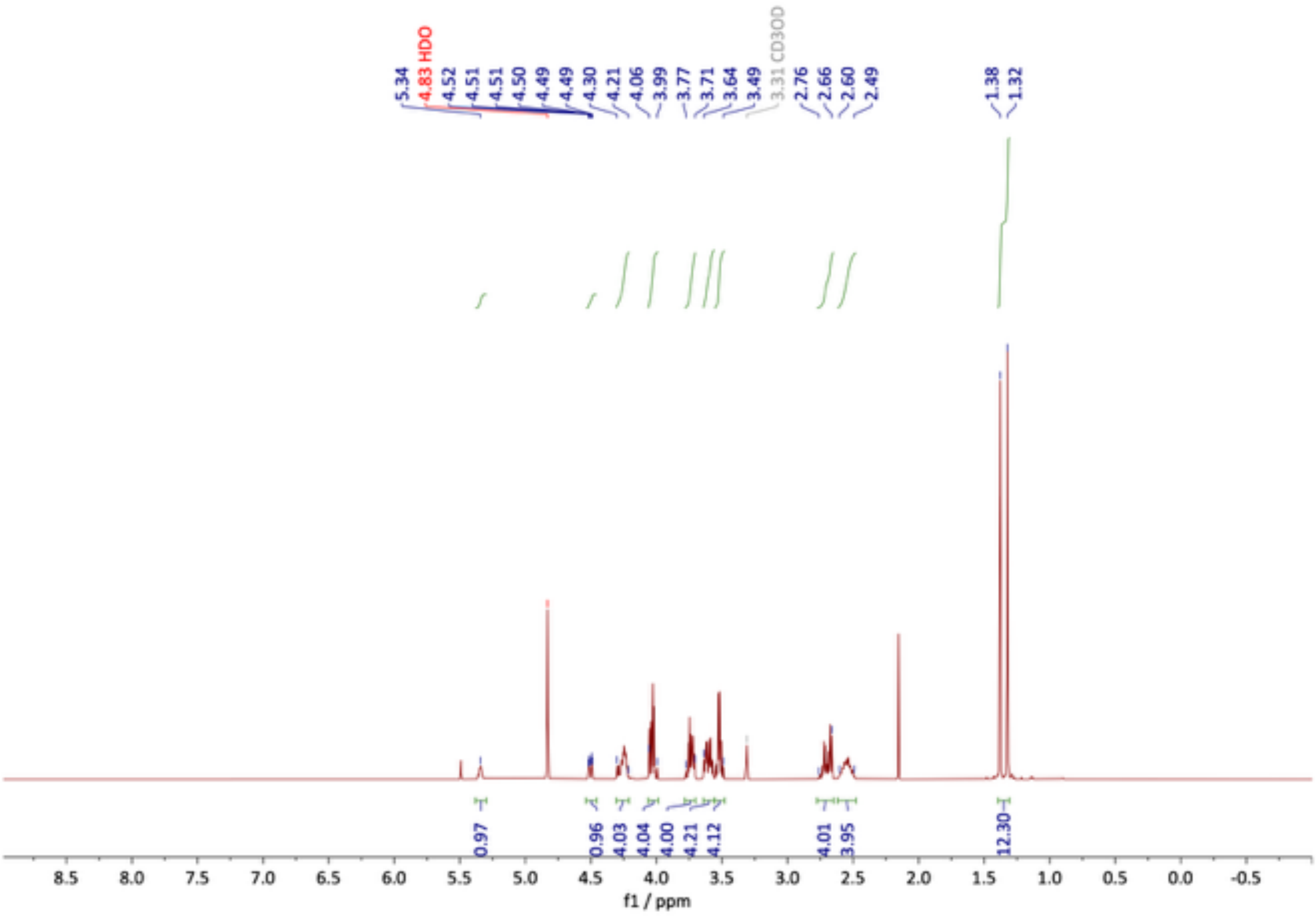
5.34
4.83 HDO
4.52
4.51
4.51
4.50
4.49
4.49
4.30
4.21
4.06
3.99
3.77
3.71
3.64
3.49
3.31 CD3OD
2.76
2.66
2.60
2.49
1.38
1.32
0.97
0.96
4.03
4.04
4.00
4.21
4.12
4.01
3.95
12.30
8.5
8.0
7.5
7.0
6.5
6.0
5.5
5.0
4.5
4.0
3.5
3.0
2.5
2.0
1.5
1.0
0.5
0.0
-0.5
f1 / ppm

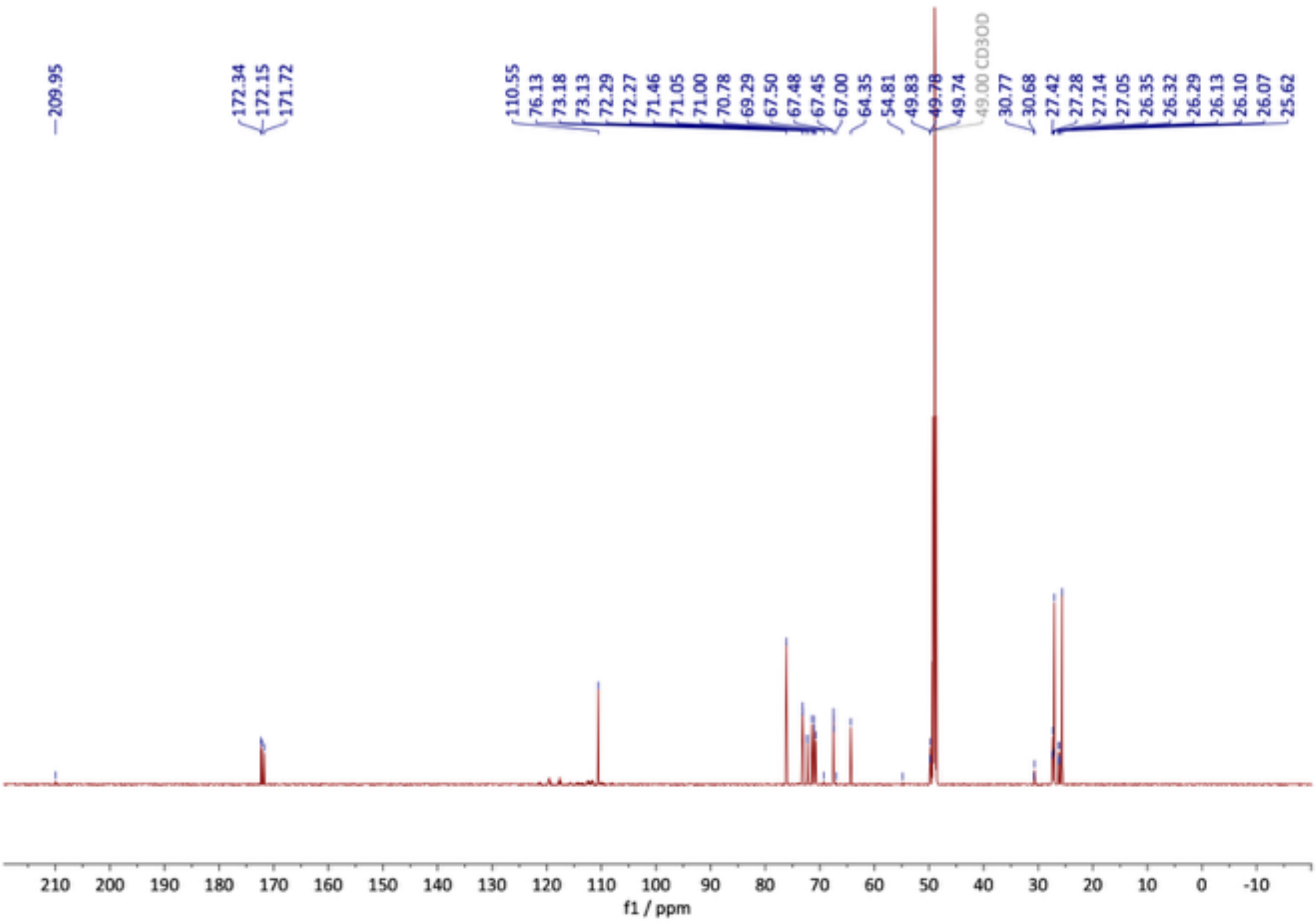
209.95
172.34
172.15
171.72
110.55
76.13
73.18
73.13
72.29
72.27
71.46
71.05
71.00
70.78
69.29
67.50
67.48
67.45
67.00
64.35
54.81
49.83
49.78
49.74
49.00 CD3OD
30.77
30.68
27.42
27.28
27.14
27.05
26.35
26.32
26.29
26.13
26.10
26.07
25.62
210
200
190
180
170
160
150
140
130
120
110
100
90
80
70
60
50
40
30
20
10
0
-10
f1 / ppm

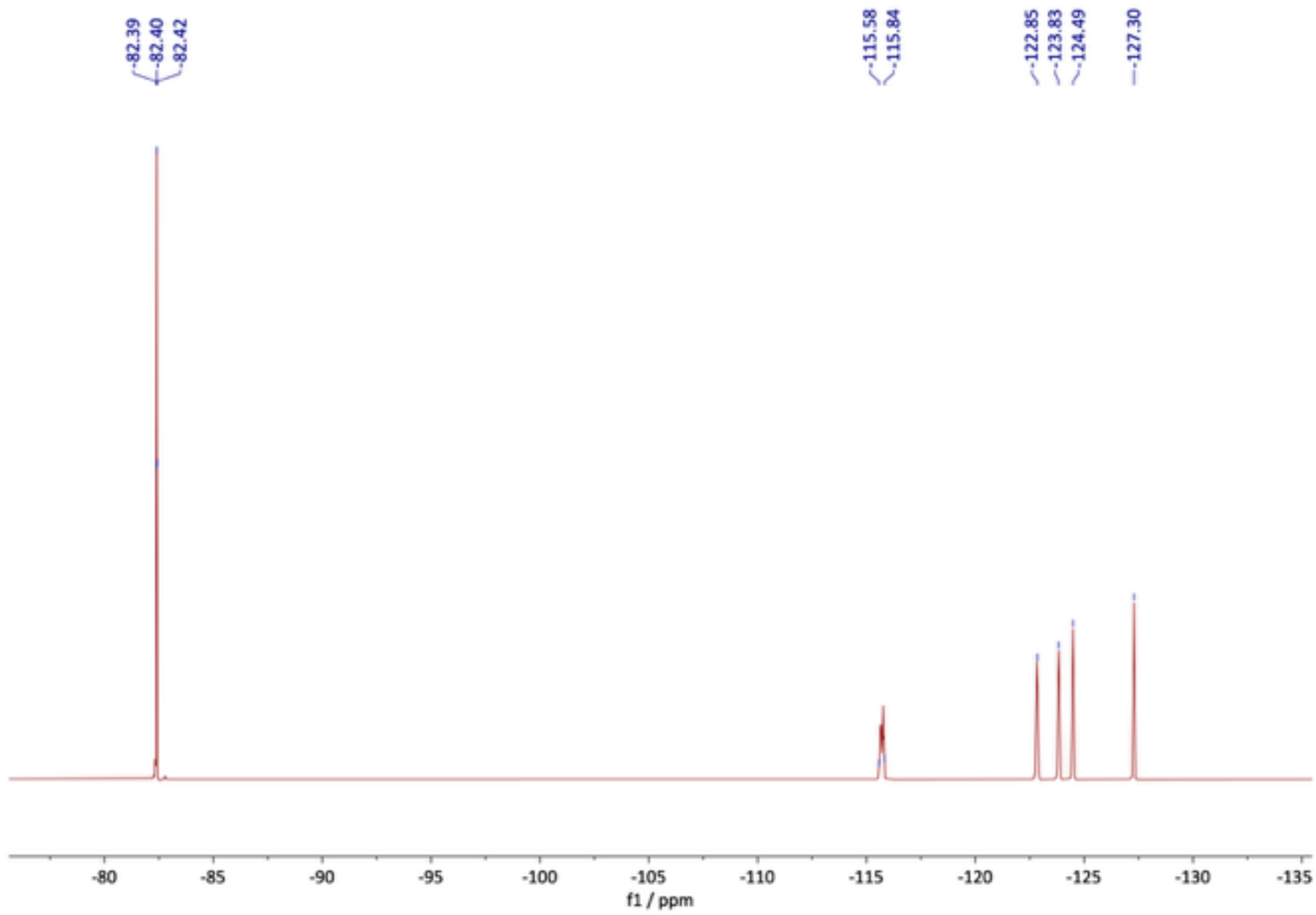

-82.39
-82.40
-82.42
-115.58
-115.84
-122.85
-123.83
-124.49
-127.30
-80
-85
-90
-95
-100
-105
-110
-115
-120
-125
-130
-135
f1 / ppm

- **G1HH**

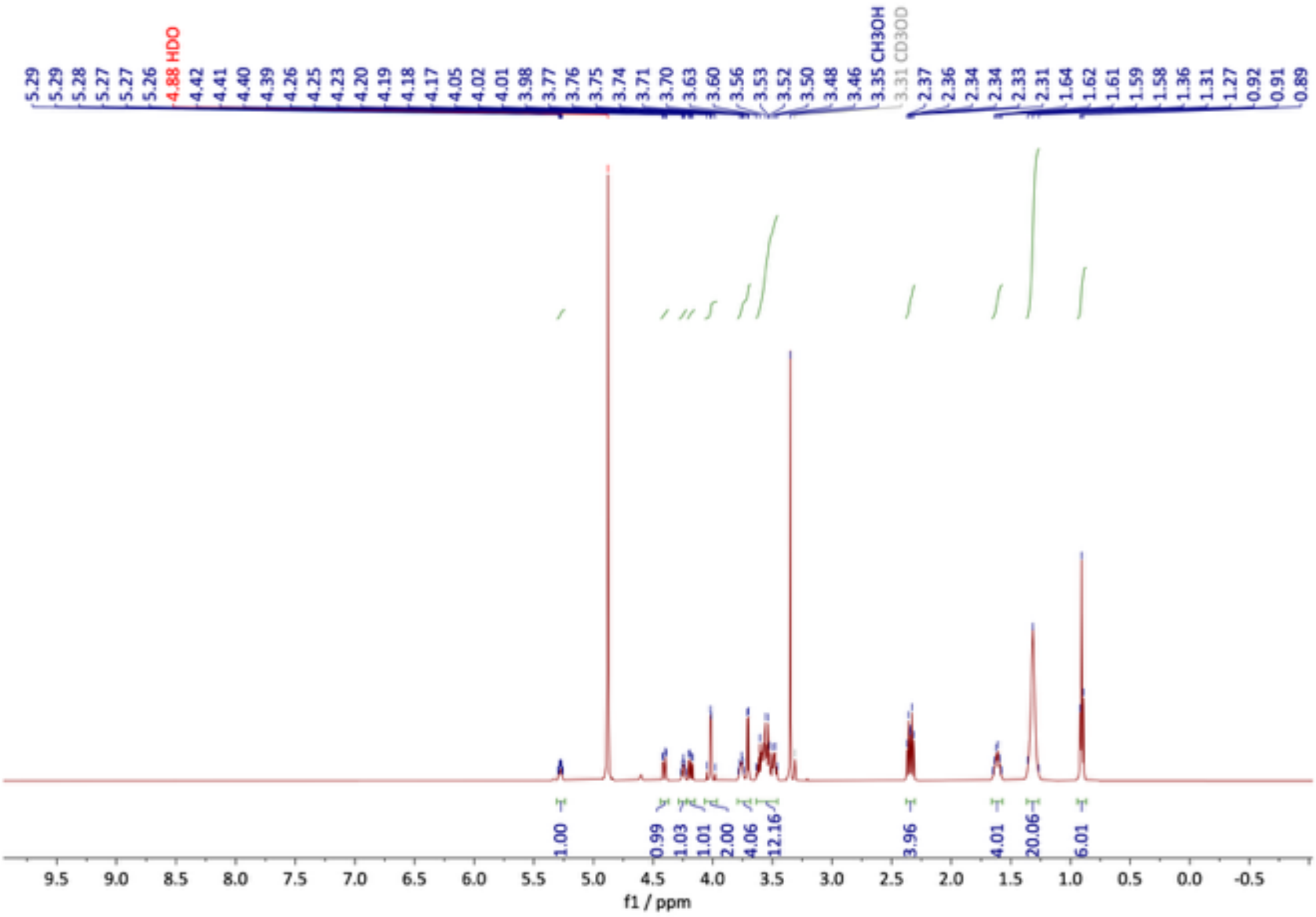
5.29
5.29
5.28
5.27
5.27
5.26
4.88 HDO
4.42
4.41
4.40
4.39
4.26
4.25
4.23
4.20
4.19
4.18
4.17
4.05
4.02
4.01
3.98
3.77
3.76
3.75
3.74
3.71
3.70
3.63
3.60
3.56
3.53
3.52
3.50
3.48
3.46
3.35 CH3OH
3.31 CD3OD
2.37
2.36
2.34
2.34
2.33
2.31
1.64
1.62
1.61
1.59
1.58
1.36
1.31
1.27
0.92
0.91
0.89
1.00
0.99
1.03
1.01
2.00
4.06
12.16
3.96
4.01
20.06
6.01
9.5
9.0
8.5
8.0
7.5
7.0
6.5
6.0
5.5
5.0
4.5
4.0
3.5
3.0
2.5
2.0
1.5
1.0
0.5
0.0
-0.5
f1 / ppm

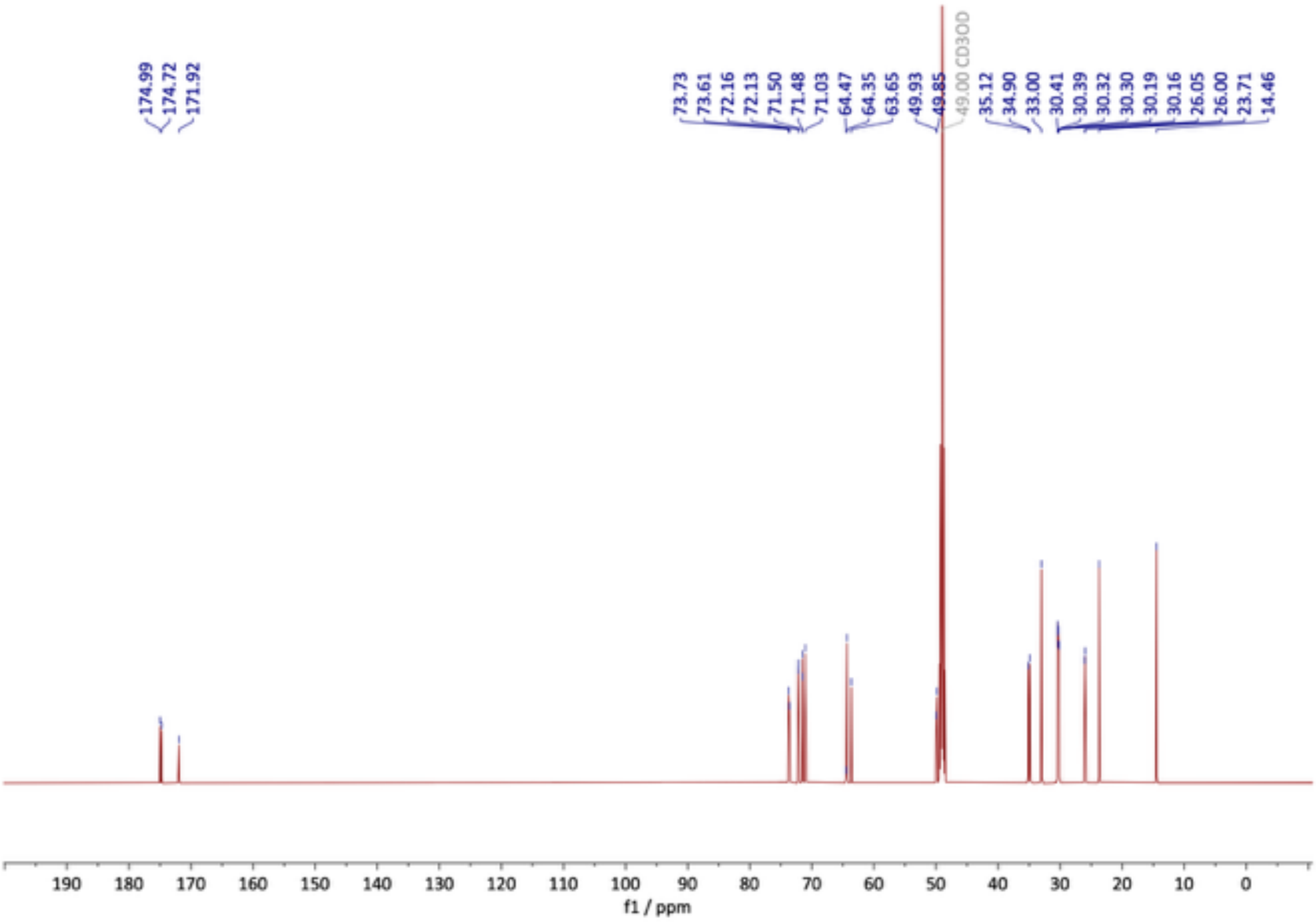
174.99
174.72
171.92
73.73
73.61
72.16
72.13
71.50
71.48
71.03
64.47
64.35
63.65
49.93
49.85
49.00 CD3OD
35.12
34.90
33.00
30.41
30.39
30.32
30.30
30.19
30.16
26.05
26.00
23.71
14.46
190
180
170
160
150
140
130
120
110
100
90
80
70
60
50
40
30
20
10
0
f1 / ppm

- **G1FF**

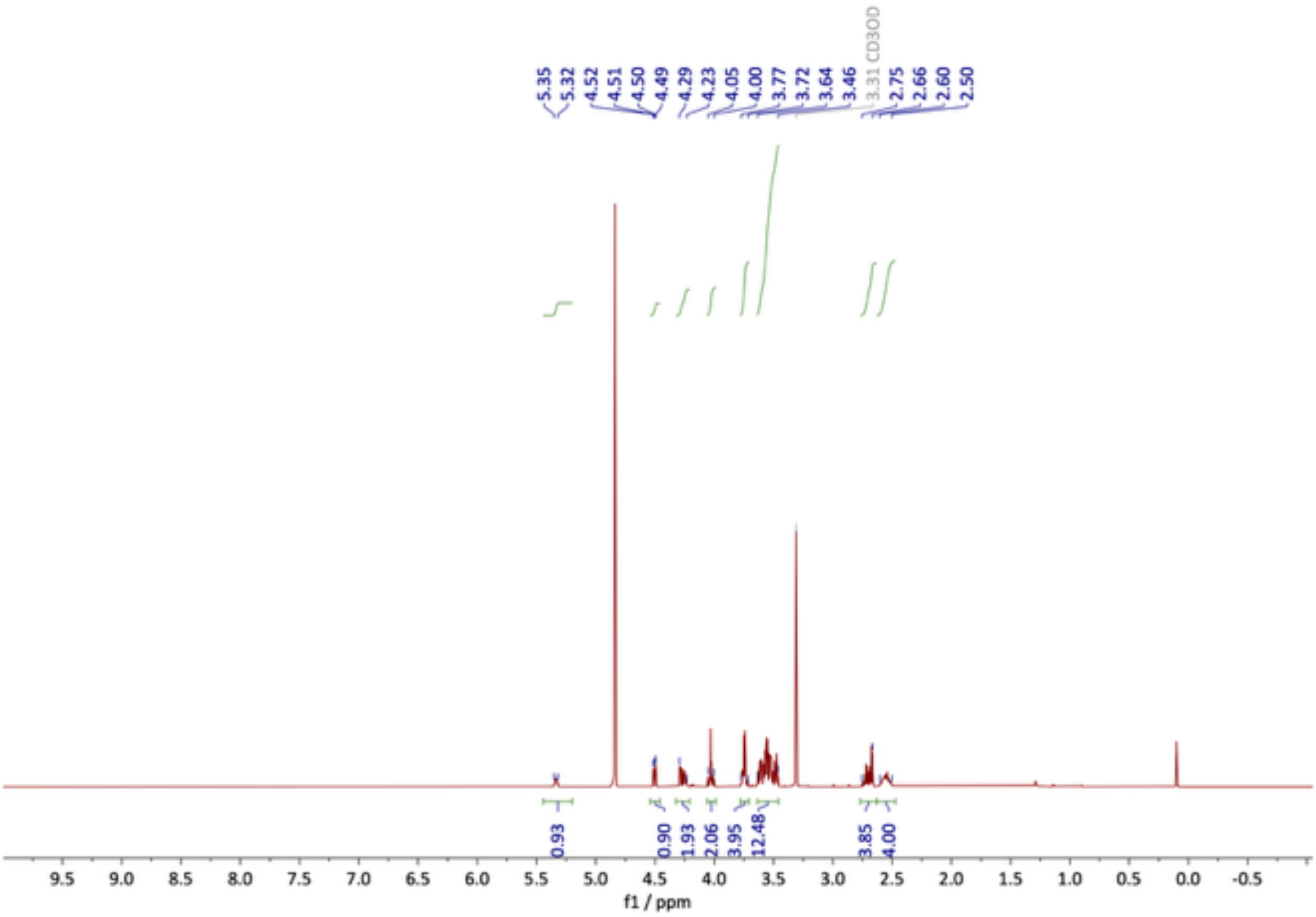
5.35
5.32
4.52
4.51
4.50
4.49
4.29
4.23
4.05
4.00
3.77
3.72
3.64
3.46
3.31 CD3OD
2.75
2.66
2.60
2.50
0.93
0.90
1.93
2.06
3.95
12.48
3.85
4.00
9.5 9.0 8.5 8.0 7.5 7.0 6.5 6.0 5.5 5.0 4.5 4.0 3.5 3.0 2.5 2.0 1.5 1.0 0.5 0.0 -0.5
f1 / ppm

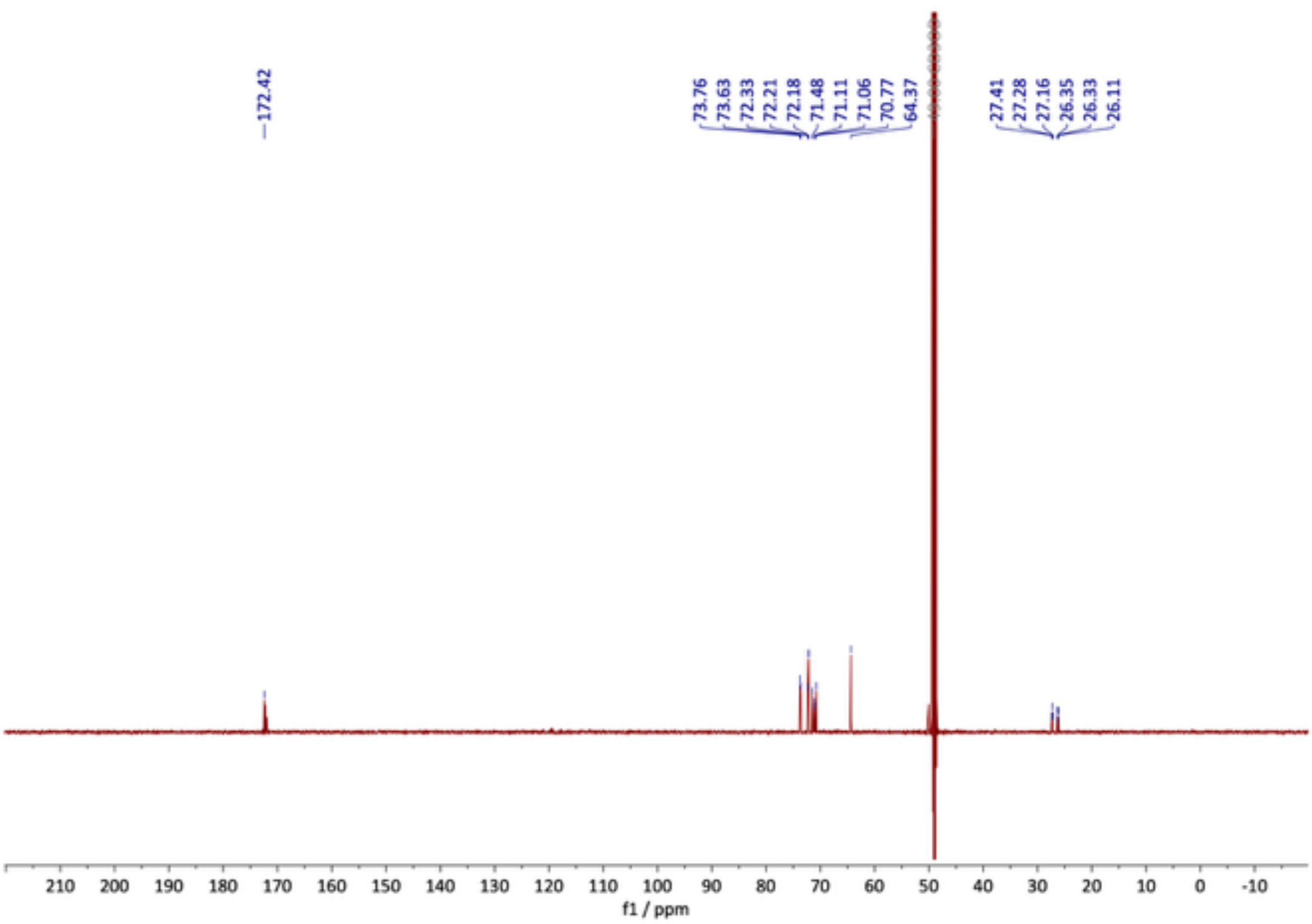
172.42
73.76
73.63
72.33
72.21
72.18
71.48
71.11
71.06
70.77
64.37
27.41
27.28
27.16
26.35
26.33
26.11
210 200 190 180 170 160 150 140 130 120 110 100 90 80 70 60 50 40 30 20 10 0 -10
f1 / ppm

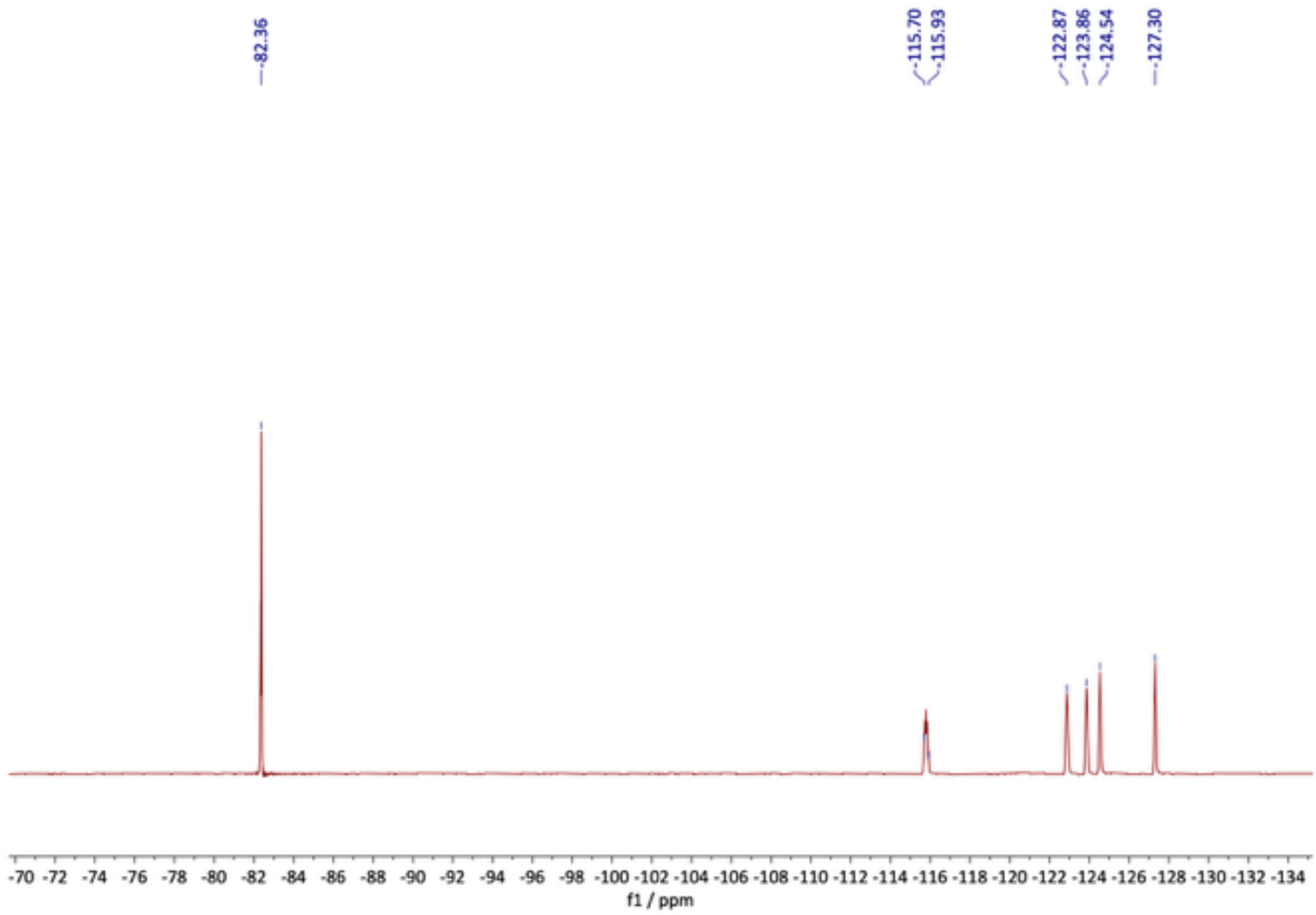

- **G1-HF**

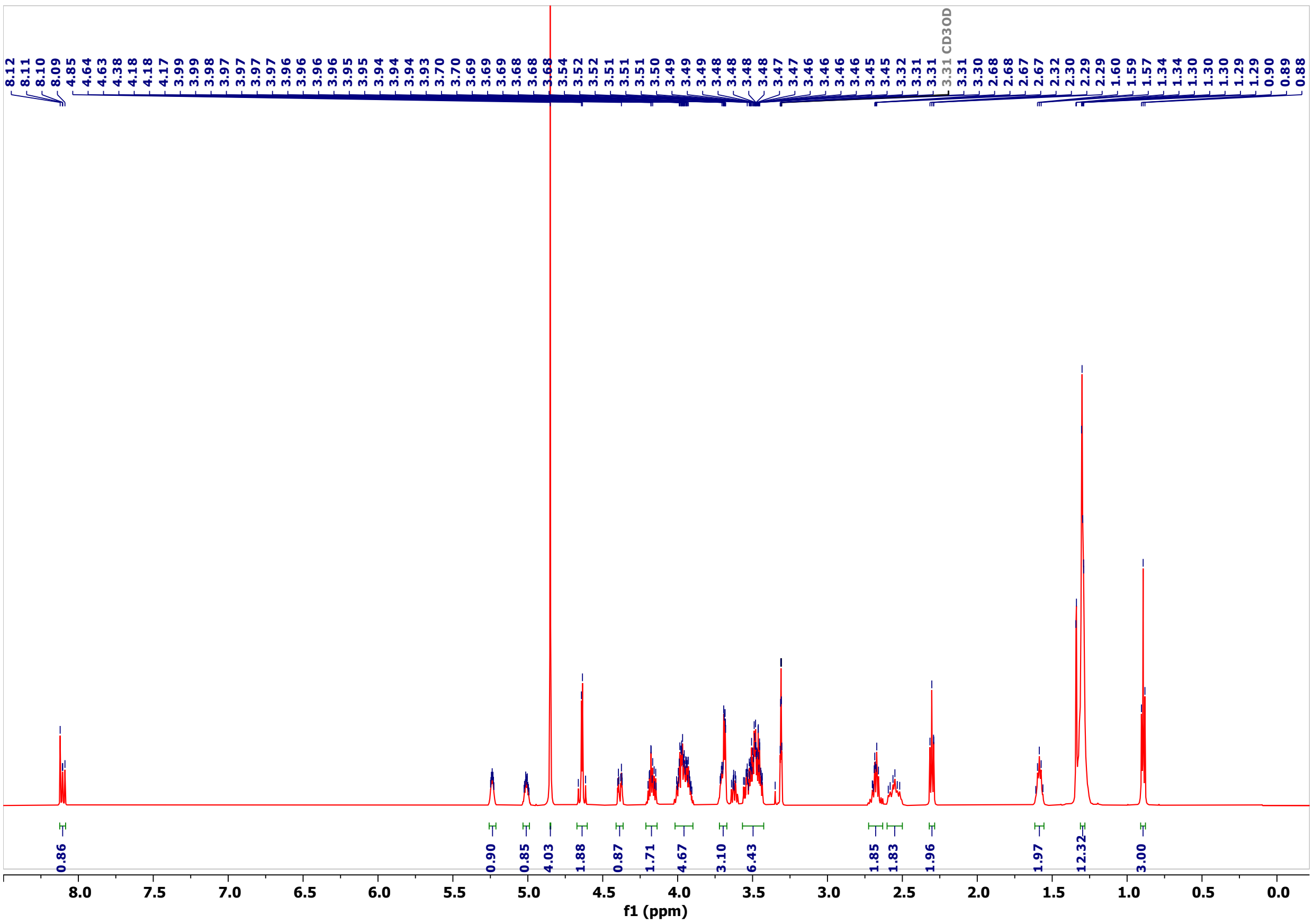

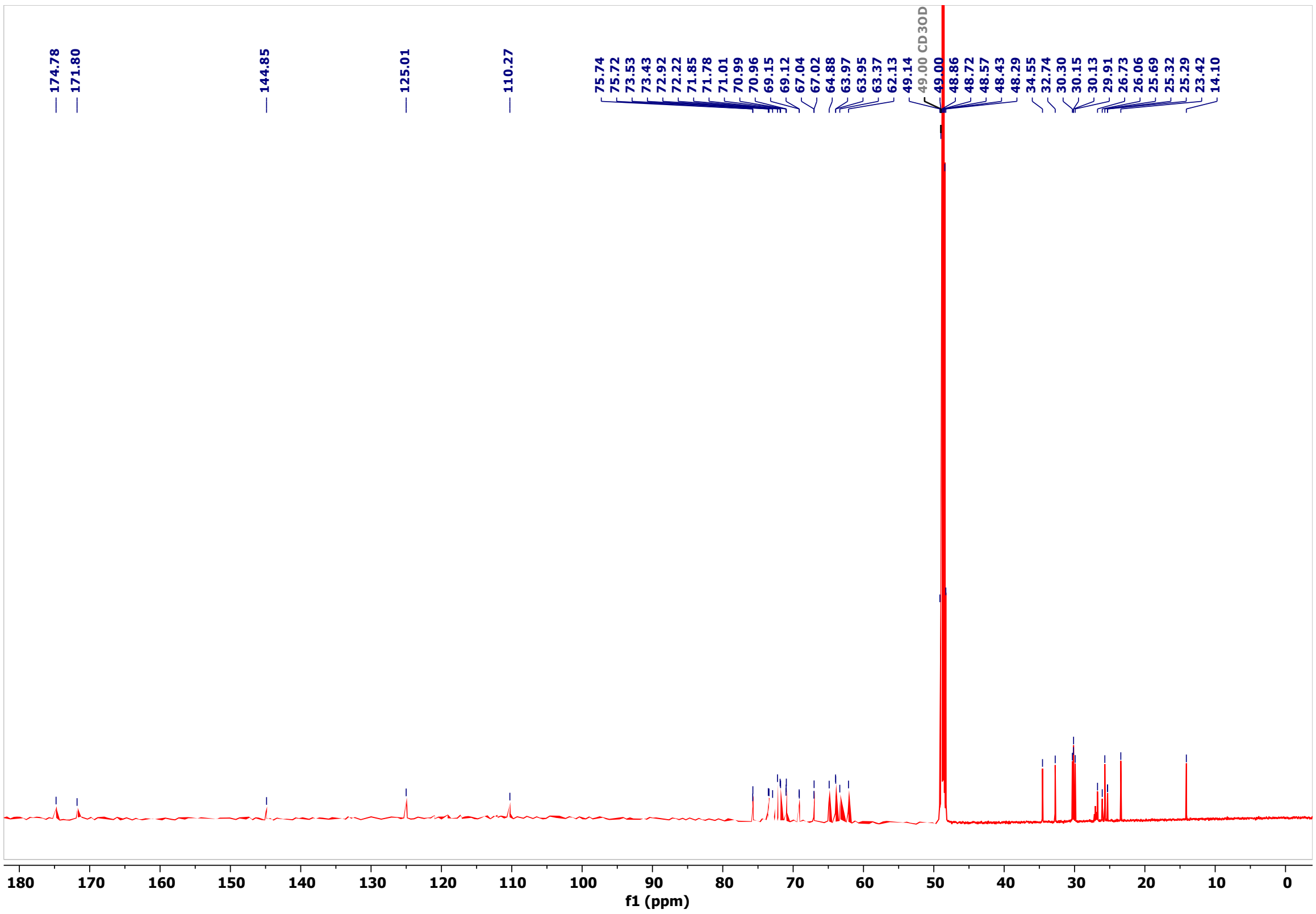

References

(1) Paul Bauer, B. H., & Erik Lindahl. *GROMACS 2022.6 Source code*. Zenodo, 2023. https://doi.org/10.5281/zenodo.8134375 (accessed).

(2) Humphrey, W.; Dalke, A.; Schulten, K. VMD: Visual molecular dynamics. *Journal of Molecular Graphics* **1996**, *14* (1), 33-38. DOI: 10.1016/0263-7855(96)00018-5.

(3) MacKerell Jr, A. D.; Bashford, D.; Bellott, M.; Dunbrack Jr, R. L.; Evanseck, J. D.; Field, M. J.; Fischer, S.; Gao, J.; Guo, H.; Ha, S. All-atom empirical potential for molecular modeling and dynamics studies of proteins. *The journal of physical chemistry B* **1998**, *102* (18), 3586-3616.

(4) Brooks, B. R.; Brooks III, C. L.; Mackerell Jr, A. D.; Nilsson, L.; Petrella, R. J.; Roux, B.; Won, Y.; Archontis, G.; Bartels, C.; Boresch, S. CHARMM: the biomolecular simulation program. *Journal of computational chemistry* **2009**, *30* (10), 1545-1614. DOI: 10.1002/jcc.21287.

(5) Lee, J.; Cheng, X.; Jo, S.; MacKerell, A. D.; Klauda, J. B.; Im, W. CHARMM-GUI input generator for NAMD, GROMACS, AMBER, OpenMM, and CHARMM/OpenMM simulations using the CHARMM36 additive force field. *Biophysical journal* **2016**, *110* (3), 641a. DOI: 10.1021/acs.jctc.5b00935.

(6) Choi, Y. K.; Park, S.-J.; Park, S.; Kim, S.; Kern, N. R.; Lee, J.; Im, W. CHARMM-GUI polymer builder for modeling and simulation of synthetic polymers. *Journal of chemical theory and computation* **2021**, *17* (4), 2431-2443. DOI: 10.1021/acs.jctc.1c00169.

(7) Jo, S.; Kim, T.; Iyer, V. G.; Im, W. CHARMM-GUI: a web-based graphical user interface for CHARMM. *Journal of computational chemistry* **2008**, *29* (11), 1859-1865. DOI: 10.1002/jcc.20945.

(8) Bussi, G.; Donadio, D.; Parrinello, M. Canonical sampling through velocity rescaling. *The Journal of chemical physics* **2007**, *126* (1). DOI: 10.1063/1.2408420.

(9) Bernetti, M.; Bussi, G. Pressure control using stochastic cell rescaling. *J Chem Phys* **2020**, *153* (11), 114107. DOI: 10.1063/5.0020514 From NLM.

(10) Parrinello, M.; Rahman, A. Polymorphic transitions in single crystals: A new molecular dynamics method. *Journal of Applied Physics* **1981**, *52* (12), 7182-7190. DOI: 10.1063/1.328693 (acccessed 10/23/2025).

(11) Marrink, S. J.; Risselada, H. J.; Yefimov, S.; Tieleman, D. P.; De Vries, A. H. The MARTINI force field: coarse grained model for biomolecular simulations. *The journal of physical chemistry B* **2007**, *111* (27), 7812-7824. DOI: 10.1021/jp071097f.

(12) Souza, P. C.; Alessandri, R.; Barnoud, J.; Thallmair, S.; Faustino, I.; Grünewald, F.; Patmanidis, I.; Abdizadeh, H.; Bruininks, B. M.; Wassenaar, T. A. Martini 3: a general purpose force field for coarse-grained molecular dynamics. *Nature methods* **2021**, *18* (4), 382-388. DOI: 10.1038/s41592-021-01098-3.

(13) Alessandri, R.; Barnoud, J.; Gertsen, A. S.; Patmanidis, I.; de Vries, A. H.; Souza, P. C. T.; Marrink, S. J. Martini 3 Coarse-Grained Force Field: Small Molecules. *Advanced Theory and Simulations* **2022**, *5* (1), 2100391. DOI: 10.1002/adts.202100391.

(14) Mirzaalipoor A, N. M., Sharif F, Ghermezcheshme H, Netz RN, Makki H. Parameter Optimization for Accurate Coarse-Grained Modeling of Perfluorocarbon Phase Behavior. *ChemRxiv* **2025**. DOI: 10.26434/chemrxiv-2025-8h3d3.

(15) Yan, B.; Alessandri, R.; Marrink, S. J.; Lee, L. S.; Liu, J. Insight into the Self-Assembly Behaviors of Per- and Polyfluoroalkyl Substances Using a "Computational Microscope". *Environmental Science & Technology Letters* **2025**, *12* (5), 626-631. DOI: 10.1021/acs.estlett.4c01081.

(16) Rashmi, R.; Hasheminejad, H.; Herziger, S.; Mirzaalipour, A.; Singh, A. K.; Netz, R. R.; Böttcher, C.; Makki, H.; Sharma, S. K.; Haag, R. Supramolecular Engineering of Alkylated, Fluorinated, and Mixed Amphiphiles. *Macromolecular rapid communications* **2022**, *43* (8), 2100914. DOI: 10.1002/marc.202100914.

(17) Jorgensen, W. L.; Maxwell, D. S.; Tirado-Rives, J. Development and Testing of the OPLS All-Atom Force Field on Conformational Energetics and Properties of Organic Liquids. *Journal of the American Chemical Society* **1996**, *118* (45), 11225-11236. DOI: 10.1021/ja9621760.

(18) Duarte, P.; Silva, M.; Rodrigues, D.; Morgado, P.; Martins, L. F. G.; Filipe, E. J. M. Liquid Mixtures Involving Hydrogenated and Fluorinated Chains: (p, ρ, T, x) Surface of (Ethanol + 2,2,2-Trifluoroethanol), Experimental and Simulation. *The Journal of Physical Chemistry B* **2013**, *117* (33), 9709-9717. DOI: 10.1021/jp3105387.

(19) Lepori, L.; Matteoli, E.; Spanedda, A.; Duce, C.; Tiné, M. R. Volume changes on mixing perfluoroalkanes with alkanes or ethers at 298.15 K. *Fluid Phase Equilibria* **2002**, *201* (1), 119-134. DOI: 10.1016/S0378-3812(02)00069-9.

(20) Borges-Araújo, L.; Borges-Araújo, A. C.; Ozturk, T. N.; Ramirez-Echemendia, D. P.; Fábián, B.; Carpenter, T. S.; Thallmair, S.; Barnoud, J.; Ingólfsson, H. I.; Hummer, G.; et al. Martini 3 Coarse-Grained Force Field for Cholesterol. *Journal of Chemical Theory and Computation* **2023**, *19* (20), 7387-7404. DOI: 10.1021/acs.jctc.3c00547.

(21) Jo, S.; Lim, J. B.; Klauda, J. B.; Im, W. CHARMM-GUI Membrane Builder for mixed bilayers and its application to yeast membranes. *Biophys J* **2009**, *97* (1), 50-58. DOI: 10.1016/j.bpj.2009.04.013 From NLM.

(22) Feng, S.; Park, S.; Choi, Y. K.; Im, W. CHARMM-GUI membrane builder: past, current, and future developments and applications. *Journal of chemical theory and computation* **2023**, *19* (8), 2161-2185. DOI: 10.1021/acs.jctc.2c01246.

(23) Gupta, R.; Badhe, Y.; Mitragotri, S.; Rai, B. Permeation of nanoparticles across the intestinal lipid membrane: dependence on shape and surface chemistry studied through molecular simulations. *Nanoscale* **2020**, *12* (11), 6318-6333, 10.1039/C9NR09947F. DOI: 10.1039/C9NR09947F.

(24) Nguyen, H. L.; Man, V. H.; Li, M. S.; Derreumaux, P.; Wang, J.; Nguyen, P. H. Elastic moduli of normal and cancer cell membranes revealed by molecular dynamics simulations. *Physical Chemistry Chemical Physics* **2022**, *24* (10), 6225-6237. DOI: 10.1039/D1CP04836H.

(25) Gorse, A.; Yordanova, V.; Bodosa, J.; Mathelié-Guinlet, M.; Walrant, A.; Taib-Mamaar, N.; Grélard, A.; François-Martin, C.; Baccouch, R.; Rascol, E. Membrane Partition and Structural Reorganization Induced by Antipsychotics with Distinct Clinical Profiles. *ACS Chemical Neuroscience* **2025**. DOI: 10.1021/acschemneuro.5c00339.

(26) Ingólfsson, H. I.; Melo, M. N.; Van Eerden, F. J.; Arnarez, C.; Lopez, C. A.; Wassenaar, T. A.; Periole, X.; De Vries, A. H.; Tieleman, D. P.; Marrink, S. J. Lipid organization of the plasma membrane. *Journal of the american chemical society* **2014**, *136* (41), 14554-14559. DOI: 10.1021/ja507832e.

(27) Jan Akhunzada, M.; D'Autilia, F.; Chandramouli, B.; Bhattacharjee, N.; Catte, A.; Di Rienzo, R.; Cardarelli, F.; Brancato, G. Interplay between lipid lateral diffusion, dye concentration and membrane permeability unveiled by a combined spectroscopic and computational study of a model lipid bilayer. *Scientific Reports* **2019**, *9* (1), 1508. DOI: 10.1038/s41598-018-37814-x.

(28) Buchoux, S. FATSLiM: a fast and robust software to analyze MD simulations of membranes. *Bioinformatics* **2016**, *33* (1), 133-134. DOI: 10.1093/bioinformatics/btw563 (acccessed 8/29/2025).

(29) Bartoš, L. *Order - Code for Coarse-Grained Lipid Order Calculations*. Zenodo, 2023. https://doi.org/10.5281/zenodo.8369479 (accessed).

(30) Wassenaar, T. A.; Pluhackova, K.; Böckmann, R. A.; Marrink, S. J.; Tieleman, D. P. Going Backward: A Flexible Geometric Approach to Reverse Transformation from Coarse Grained to Atomistic Models. *Journal of Chemical Theory and Computation* **2014**, *10* (2), 676-690. DOI: 10.1021/ct400617g.

(31) Willard, A. P.; Chandler, D. Instantaneous liquid interfaces. *The Journal of Physical Chemistry B* **2010**, *114* (5), 1954-1958. DOI: 10.1021/jp909219k.

(32) Duran, T.; Costa, A.; Gupta, A.; Xu, X.; Zhang, H.; Burgess, D.; Chaudhuri, B. Coarse-grained molecular dynamics simulations of paclitaxel-loaded polymeric micelles. *Molecular Pharmaceutics* **2022**, *19* (4), 1117-1134. DOI: 10.1021/acs.molpharmaceut.1c00800.

(33) Kumar, S.; Rosenberg, J. M.; Bouzida, D.; Swendsen, R. H.; Kollman, P. A. The weighted histogram analysis method for free-energy calculations on biomolecules. I. The method. *Journal of computational chemistry* **1992**, *13* (8), 1011-1021. DOI: 10.1002/jcc.540130812.

(34) Arenhoevel, J.; Schmitt, A.-C.; Kerkhoff, Y.; Ahmadi, V.; Quaas, E.; Ludwig, K.; Achazi, K.; Nie, C.; Bej, R.; Haag, R. Mucin-Inspired Polymeric Fibers for Herpes Simplex Virus Type 1 Inhibition. *Macromolecular Bioscience* **2024**, *24* (9), 2400120. DOI: 10.1002/mabi.202400120.

(35) Schindelin, J.; Arganda-Carreras, I.; Frise, E.; Kaynig, V.; Longair, M.; Pietzsch, T.; Preibisch, S.; Rueden, C.; Saalfeld, S.; Schmid, B.; et al. Fiji: an open-source platform for biological-image analysis. *Nature Methods* **2012**, *9* (7), 676-682. DOI: 10.1038/nmeth.2019.

(36) Schneider, C. A.; Rasband, W. S.; Eliceiri, K. W. NIH Image to ImageJ: 25 years of image analysis. *Nature Methods* **2012**, *9* (7), 671-675. DOI: 10.1038/nmeth.2089.

(37) Wang, Z.; Cui, Y.-T.; Xu, Z.-B.; Qu, J. Hot water-promoted ring-opening of epoxides and aziridines by water and other nucleopliles. *The Journal of organic chemistry* **2008**, *73* (6), 2270-2274. DOI: 10.1021/jo702401t.

(38) Sisson, A. L.; Steinhilber, D.; Rossow, T.; Welker, P.; Licha, K.; Haag, R. Biocompatible Functionalized Polyglycerol Microgels with Cell Penetrating Properties. *Angewandte Chemie International Edition* **2009**, *48* (41), 7540-7545. DOI: 10.1002/anie.200901583.

(39) Reichert, S.; Welker, P.; Calderón, M.; Khandare, J.; Mangoldt, D.; Licha, K.; Kainthan, R. K.; Brooks, D. E.; Haag, R. Size-Dependant Cellular Uptake of Dendritic Polyglycerol. *Small* **2011**, *7* (6), 820-829. DOI: 10.1002/smll.201002220.

(40) Gröger, D.; Paulus, F.; Licha, K.; Welker, P.; Weinhart, M.; Holzhausen, C.; Mundhenk, L.; Gruber, A. D.; Abram, U.; Haag, R. Synthesis and Biological Evaluation of Radio and Dye Labeled Amino Functionalized Dendritic Polyglycerol Sulfates as Multivalent Anti-Inflammatory Compounds. *Bioconjugate Chemistry* **2013**, *24* (9), 1507-1514. DOI: 10.1021/bc400047f.

(41) Sousa-Herves, A.; Würfel, P.; Wegner, N.; Khandare, J.; Licha, K.; Haag, R.; Welker, P.; Calderón, M. Dendritic polyglycerol sulfate as a novel platform for paclitaxel delivery: pitfalls of ester linkage. *Nanoscale* **2015**, *7* (9), 3923-3932, 10.1039/C4NR04428B. DOI: 10.1039/C4NR04428B.

(42) Dimde, M.; Sahle, F. F.; Wycisk, V.; Steinhilber, D.; Camacho, L. C.; Licha, K.; Lademann, J.; Haag, R. Synthesis and Validation of Functional Nanogels as pH-Sensors in the Hair Follicle. *Macromolecular Bioscience* **2017**, *17* (10), 1600505. DOI: 10.1002/mabi.201600505.
